# C9orf72-catalyzed GTP loading of Rab39A enables HOPS-mediated membrane tethering and fusion in mammalian autophagy

**DOI:** 10.1038/s41467-023-42003-0

**Published:** 2023-10-11

**Authors:** Shen Zhang, Mindan Tong, Denghao Zheng, Huiying Huang, Linsen Li, Christian Ungermann, Yi Pan, Hanyan Luo, Ming Lei, Zaiming Tang, Wan Fu, She Chen, Xiaoxia Liu, Qing Zhong

**Affiliations:** 1 Key Laboratory of Cell Differentiation and Apoptosis of Chinese Ministry of Education, Department of Pathophysiology, Shanghai Jiao Tong University School of Medicine, Shanghai, China; 2 Osnabrück University, Department of Biology/Chemistry, Biochemistry section, Osnabrück, Germany; 3 Center of Cellular Nanoanalytic Osnabrück (CellNanOs), Osnabrück University, Osnabrück, Germany; 4 State Key Laboratory of Oncogenes and Related Genes, Ninth People’s Hospital, Shanghai Jiao Tong University School of Medicine, 200011 Shanghai, China; 5 Shanghai Institute of Precision Medicine, 200125 Shanghai, China; 6 National Institute of Biological Sciences, 102206 Beijing, China

**Keywords:** Macroautophagy, GTP-binding protein regulators, Membrane fusion

## Abstract

The multi-subunit homotypic fusion and vacuole protein sorting (HOPS) membrane-tethering complex is required for autophagosome-lysosome fusion in mammals, yet reconstituting the mammalian HOPS complex remains a challenge. Here we propose a “hook-up” model for mammalian HOPS complex assembly, which requires two HOPS sub-complexes docking on membranes via membrane-associated Rabs. We identify Rab39A as a key small GTPase that recruits HOPS onto autophagic vesicles. Proper pairing with Rab2 and Rab39A enables HOPS complex assembly between proteoliposomes for its tethering function, facilitating efficient membrane fusion. GTP loading of Rab39A is important for the recruitment of HOPS to autophagic membranes. Activation of Rab39A is catalyzed by C9orf72, a guanine exchange factor associated with amyotrophic lateral sclerosis and familial frontotemporal dementia. Constitutive activation of Rab39A can rescue autophagy defects caused by C9orf72 depletion. These results therefore reveal a crucial role for the C9orf72-Rab39A-HOPS axis in autophagosome-lysosome fusion.

## Abstract (web summary)

The HOPS complex mediates membrane tethering and autophagosome-lysosome fusion. Here, the authors biochemically reconstitute the mammalian HOPS in protoliposomes and propose a model of complex assembly that depends on Rab2 and Rab39A.

## Introduction

Multi-subunit tethering complexes (MTCs) are a group of conserved protein complexes fundamental to achieve correct intracellular vesicular transport. They specifically recognize cargo vesicles and promote interaction between two compartment membranes to facilitate proper vesicle fusion at target destinations. One of the best characterized MTC is the yeast HOPS complex (Homotypic fusion and protein sorting). Yeast HOPS was first identified in gene screening for directing vacuolar morphogenesis^1^. Yeast HOPS was found to promote homotypic fusion of vacuoles^2,3^ and heterotypic fusion between vacuoles/lysosomes and late endosome/MVB (multi-vesicular bodies), autophagosome or AP3 vesicles^4^ by facilitating these organelles tethering^5^, SNAREs assembly^6^ and fusion pores formation^7^. Later, it was reported that HOPS function is conserved in mammalian cells. Deficiency of HOPS in mammalian cells showed inhibited endosome-lysosome fusion^8^ and autophagosome-lysosome fusion^9^.

It is well accepted Rab GTPases participate in the regulation of MTCs’ specificity in transport directionality and cargo vesicle recognition. The Rab proteins are a superfamily of Ras-like GTPase^10^. After synthesized, the C-terminal one or two cysteines of Rab GTPase will be prenylated by GGT (geranylgeranyl transferase) and delivered to the cognate membrane by regulation of Rab GDI (GDP dissociation inhibitor) and Rab GDF (GDI displacement factor)^11,12^. Then Rab GTPase switches from the inactive GDP-bound form to active GTP-bound form by specific GEF (GDP/GTP exchange factor), which enables effector proteins including MTCs to bind and complete vesicle trafficking function^13^. After this, GAP (GTPase-activating protein) facilitates hydrolysis of GTP of Rab and subsequent recruitment of GDI (GDP-dissociation inhibitor) to retrieve the GDP-bound Rab from intracellular membrane for re-association with membranes^14–16^. Specifically, the recruitment by distinct Rab GTPase defines the function of the HOPS complex in multiple vesicle transport pathways^17^.

In yeast, the HOPS complex binds two molecules of the vacuolar Rab GTPase Ypt7 (the yeast orthologue of Rab7) via its Vps39 and Vps41 subunits to facilitate membrane tethering and SNARE-mediated fusion. The function of Ypt7-HOPS-SNAREs in yeast vacuole fusion is recapitulated with reconstituted proteoliposomes^18^. In mammalian cells, Rab2, Rab7, and Arl8 were reported to participate in the recruitment of HOPS in autophagy or endocytic fusion^19–21^. For autophagosome-lysosome membrane fusion, Rab2 is able to recruit mammalian HOPS complex to autophagosome, while Rab7 likely participates through its mediator protein PLEKHM1 on lysosome in HOPS mediated fusion^22,23^. Whether these regulatory Rab proteins cooperate with six-subunit HOPS complex and SNAREs in a biochemically reconstituted system in mammals remains to be determined.

In this study, we find that an STX17/HOPS-associated, lysosome-resident Rab GTPase, Rab39A, mediates autophagosome and lysosome fusion through recruitment of HOPS. Using reconstituted proteoliposomes, we show that two mammalian HOPS subcomplexes, associated with membranes through Rab2 and Rab39A, respectively, assembled through a “hook-up” mode into a functional tethering complex to mediate membrane tethering and SNAREs mediated membrane fusion. GTP loading of Rab39A is important for its membrane localization and HOPS recruitment. Importantly, C9orf72 involved in ALS/FTD pathology directly promotes GTP loading of Rab39A, HOPS recruitment and autophagosome-lysosome fusion both in vivo and in vitro. In sum, we establish an in vitro reconstituted proteoliposome system bearing mammalian autophagic SNAREs, HOPS, and Rab GTPases to illustrate the cooperative function between human HOPS and Rab GTPases to promote autophagic membrane fusion and propose that the C9orf72-Rab39A-HOPS axis links autophagosome-lysosome fusion with ALS/FTD pathology.

## Results

### Futile reconstitution of Human HOPS complex with Rab2 and Rab7 in STX17-SNAP29-VAMP8-mediated lipid mixing in vitro

**Fig. 1:**
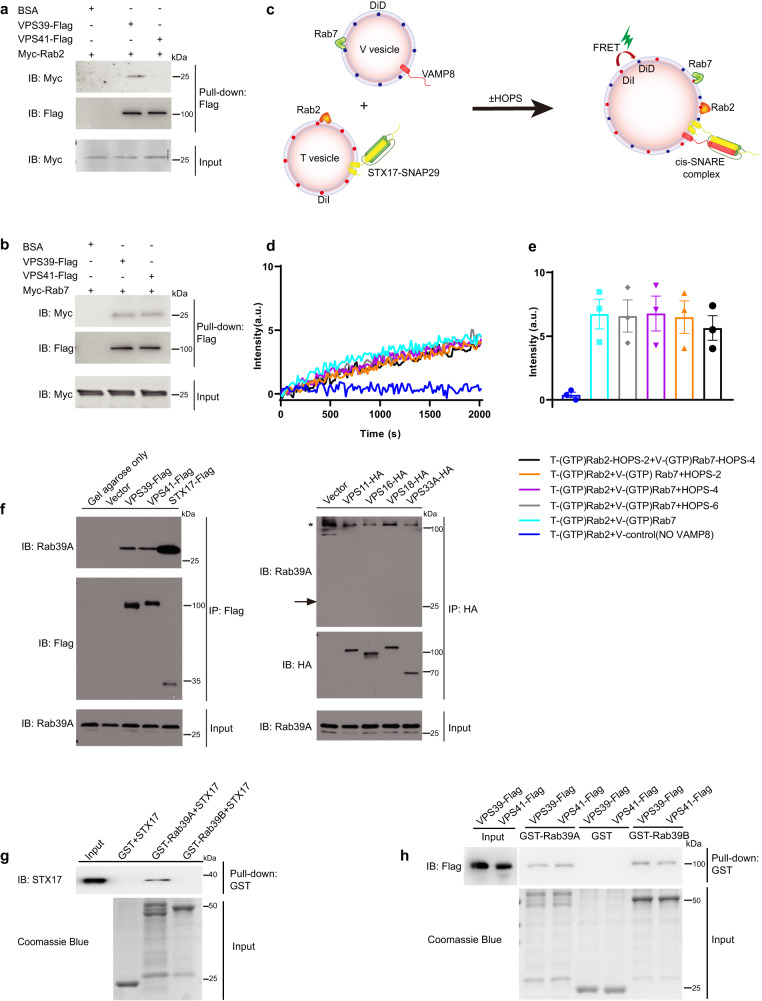
Reconstitution of Human HOPS complex with Rab2 and Rab7 in STX17-SNAP29-VAMP8 mediated lipid mixing in vitro. **a**, **b** VPS39-Flag or VPS41-Flag directly interacts with Myc-Rab2 or Myc-Rab7. Flag M2 agarose was blocked with BSA or conjugated with purified VPS39-Flag or VPS41-Flag from 293 S cells, then incubated with purified Myc-Rab2 (**a**) or Myc-Rab7 (**b**) from *E. coli* respectively, and then washed and eluted for immunoblotting by indicated antibodies. **c** Schematic drawing of lipid mixing assay with in vitro reconstituted proteoliposomes. **d** Futile reconstitution of Human HOPS complex with Rab2 and Rab7 in STX17-SNAP29-VAMP8 mediated lipid mixing. HOPS-2, VPS39/VPS11 subcomplex of HOPS complex; HOPS-4, VPS16/VPS18/VPS33A/ VPS41 subcomplex of HOPS complex; T, proteoliposomes incorporated with t-SNAREs (STX17-SNAP29); V, proteoliposomes incorporated with v-SNARE (VAMP8); a.u., arbitrary units. **e** Quantitative of results of (**d**) at around 2000 sec from three independent replicated experiments. a.u., arbitrary units. Data are presented as mean values ± SEM. **f** Endogenous Rab39A interacts with HOPS complex subunits, majorly VPS39 and VPS41, and STX17, but not interacting with VPS11, VPS16, VPS18, VPS33A. 293T cells were transfected with empty vector or tagged protein expression plasmids as indicated. 24 h after transfection, cells were lysed and immunoprecipitated with Flag M2 agarose (Left panel) or HA agarose (Right panel). Immunoblotting was performed with indicated antibodies. *, non-specific band. Black arrow indicates endogenous Rab39A band. **g** STX17 directly interacts with Rab39A, but not Rab39B. Glutathione Sepharose beads bound with GST, GST-Rab39A, or GST-Rab39B were incubated with purified STX17 for one hour, and then washed and boiled for immunoblotting. **h** Direct binding of GST-Rab39A or GST-Rab39B to VPS39-Flag or VPS41-Flag in vitro. Glutathione Sepharose beads bound with GST, GST-Rab39A, or GST-Rab39B were incubated with purified VPS39-Flag or VPS41-Flag for one hour, and then washed and boiled for immunoblotting. Source data are provided as a Source Data file.

Proper membrane tethering activity of the HOPS complex requires Rab GTPases for the initial membrane contact and subsequent recruitment through protein interaction. It was reported that the autophagosome-associated Rab2A (refer to Rab2 throughout the text) and lysosome-associated Rab7A (refer to Rab7 throughout the text) are involved in the autophagosome-lysosome fusion process^22,24,25^. We aimed to demonstrate biochemically that these two Rabs could support HOPS-mediated tethering and promote SNARE-mediated fusion. We first checked the direct interaction between the mammalian HOPS complex and these two Rab GTPases. The yeast HOPS complex is comprised of six subunits, Vps11, Vps16, Vps18, Vps33, Vps39, and Vps41 with a flexible elongated seahorse-like structure^26^. Both ends of the HOPS complex contain a Rab-binding subunit: Vps39 and Vps41. As yeast HOPS binds Ypt7 with its Vps39 and VPs41 subunits on two opposite ends of complex, we tested if purified human homolog VPS41 and VPS39 bind to Rab2 and Rab7 in in vitro pull-down assays. We found that Rab2 specifically binds to VPS39, but not VPS41 (Fig. 1a), and Rab7 binds to both VPS39 and VPS41 (Fig. 1b).

Next, we examined if the HOPS complex associated with Rab2 and Rab7 could promote autophagic SNAREs-mediated membrane fusion in a biochemical reconstitution system. Since the HOPS Core C complex consisting of VPS11, VPS16, VPS18, and VPS33A was also shared by the CORVET (class C core vacuole/endosome tethering) complex in endosome tethering^2,3,27,28^, we attempted to purify the human HOPS complex from 293S cells with overexpression of all six subunits via an affinity purification tag on the tip subunit VPS41 (Fig. S1a–c) or VPS39 (Fig. S1d–g). Interestingly, unlike in yeast^29^, tandem affinity purification via VPS41 or VPS39 failed to pull-down the HOPS six-subunit holo-complex, instead, VPS41 co-purified with VPS16, VPS18, VPS33A (Fig. S1b, c), and VPS39 co-purified with VPS11 (Fig. S1e–g), raising a possibility that the HOPS complex forms two stable subcomplexes in mammalian cells. This notion was supported by the observation that the endogenous HOPS complex also fell apart into two major fractions by Size-Exclusion Chromatography (SEC) with the protein complex sizes coincident with the four-subunit and two-subunit of the HOPS complex (Fig. S1h).

We examined whether the six-subunit HOPS complex could form by incubation of purified two-subunit VPS39-VPS11 subcomplex and four-subunit VPS41-VPS16-VPS18-VPS33A subcomplex in vitro. The SEC was applied to analyze the protein mixture after incubation and the elution fractions were subjected to SDS-PAGE analysis. Compared with four-subunit HOPS subcomplex or two-subunit HOPS subcomplex alone, the incubated sample showed an elution peak shift in both the SEC profile and SDS-PAGE, which indicated the assembly of the six-subunit HOPS complex after incubation (Fig. S1i). This was also validated by Western Blot using antibodies against each subunit (Fig. S1j and k). Furthermore, Mass Spectrometry was used to analyze the maximum elution fraction of the assembled complex, and the stoichiometry of the assembled HOPS complex was determined. The results verified the presence of all six subunits with roughly equal abundance (Fig. S1l). These results suggest that the assembly of the six-subunit HOPS complex is possible by incubating four-subunit HOPS subcomplex and two-subunit HOPS subcomplex in vitro.

Next, we co-expressed the mammalian Rab2 or Rab7 GTPases in 293 S cells with Rab geranylgeranyl transferase, RGGT2, containing Rab GGTA and Rab GGTB, and Rab escort protein, REP1 to generate prenylated Rabs (Fig. S2a). The membrane association ability of Rab GTPases was confirmed by liposomes co-flotation assay (Fig. S2b), as the Rab GTPases with C-terminal prenylation was able to be reconstituted on the liposomes and enriched on the top fraction of histodenz gradient after centrifugation (Fig. S2c), and the unmodified form of Rabs normally accumulated at lower fractions (Fig. S2d).

We aimed to establish an in vitro reconstituted biochemical system to recapitulate the fusogenic activity of autophagic membrane tethers and SNAREs in a lipid mixing assay (Fig. 1c). First, we reconstituted STX17-SNAP29 and prenylated Rab2 liposomes with 2% DiI (referred to as T-Rab2) and VAMP8 and prenylated Rab7 liposomes with 2% DiD (referred to as V-Rab7). Then, the Rab GTPases on proteoliposomes were activated by loading GTP via a chemical method^30^. We analyzed lipid mixing between T-Rab2-GTP liposomes and V-Rab7-GTP liposomes by monitoring the DiI-DiD FRET signal which is activated by lipid mixing. Positive FRET signal indicating the lipid mixing between T-Rab2-GTP and V-Rab7-GTP (Fig. 1d, e, cyan) suggesting that this lipid mixing is dependent on the assembly of the SNARE complex, as the lack of VAMP8 displayed no lipid mixing (Fig. 1d, e, blue). This experiment suggests that these Rabs incorporated proteoliposomes are capable of fusion in the presence of the functional SNARE complex. Next, we added HOPS into the lipid mixing system. Interestingly, the introduction of pre-assembled HOPS-6 didn’t speed up lipid mixing between T-Rab2 and V-Rab7 (Fig. 1d, e, gray VS cyan), neither in HOPS-2 (Fig. 1d, e, orange) or HOPS-4 (Fig. 1d, e, purple) added groups. Furthermore, we attempted to pre-incubate of HOPS-2 and HOPS-4, with T-Rab2 and V-Rab7 respectively. However, even in the HOPS containing samples, we did not observe an augmented FRET signal compared to samples without HOPS (Fig. 1d, e, black VS cyan). This implied that either HOPS-6 is not assembled correctly between T-Rab2 and V-Rab7, or Rab2 and Rab7 were unable to activate HOPS-6. It raised a possibility that, at least in the mammalian system, Rab2 and Rab7 we used might not be the right pair to directly recruit and activate HOPS complex to membrane and to couple with autophagic SNARE fusogenic activity, and only with appropriate Rabs, the mammalian HOPS could promote autophagic SNARE-mediated membrane fusion.

### Rab39A/B are HOPS complex associated Rab GTPases involved in autophagy

To search for Rabs associated with autophagic SNAREs and HOPS, we employed Tandem affinity purification/ Mass spectrometry (TAP-MS) for proteins associated with autophagic SNARE STX17 in 293T cells. We then noticed Rab39A, as the most enriched Rab GTPases associated with STX17. Meanwhile, we also found some known interacting proteins of STX17 in the MS results, like VAMP8, YKT6, VPS16, etc. (Supplementary Data 1). The immunoprecipitation assay confirmed the interaction between ectopic STX17 and endogenous Rab39A (Fig. 1f) or overexpressed Rab39A (Fig. S3a). Rab39A is a member of Ras-GTPase family, which is evolutional conserved in human and other mammalians. Since human Rab39A shares about 77% amino acid sequence identity with its isoform Rab39B (Fig. S3b), we also checked the binding between Rab39B and STX17. The IP results showed that the ectopically expressed STX17 immunoprecipitated with overexpressed Rab39B (Fig. S3a). However, the pull-down assay showed that only purified Rab39A has a direct interaction with STX17, but not its isoform Rab39B (Fig. 1g). Besides, we examined the interaction between both isoforms and other autophagic SNAREs. The pull-down results showed that none of SNAP29, VAMP8 or YKT6 exhibited direct interaction with Rab39A or Rab39B (Fig. S3c–e).

Next, we checked the interaction between HOPS and both Rab39 isoforms. We found all overexpressed HOPS subunits immunoprecipitated with ectopic Rab39A and Rab39B (Fig. S3a), while only overproduced VPS39 and VPS41 immunoprecipitated with endogenous Rab39A (Fig. 1f) and endogenous Rab39B (Fig. S4a). To further dissect the direct interaction between Rab39A/B and the HOPS complex, we carried out reciprocal pull-down assays and the results showed that both Rab39A and Rab39B directly bind with VPS39 and VPS41 to the same extent (Fig. 1h, Fig. S4b, c).

Moreover, we investigated the intracellular distribution of Rab39 isoforms and their co-localization with STX17 in autophagy-induced condition by Torin 1 treatment. Rab39A formed punctate structures and showed intensive colocalization with STX17 in autolysosome structures stained by both autophagosomal marker LC3 and lysosomal marker LAMP2 (Fig. S4d, e). Rab39B also showed punctate structure but with more cytosolic distribution. Besides, Rab39B puncta colocalized less with STX17 on autolysosomes, compared with Rab39A (Fig. S4f–h).

These results imply that both Rab39A and Rab39B are STX17-associated, HOPS complex-interacting proteins. Besides, Rab39A and Rab39B may own distinct roles in autophagy according to their distinct binding behaviors with STX17 and different distribution patterns in cells.

### Rab39A promotes autophagosome-lysosome fusion to regulate autophagy, while Rab39B has mild effect on autophagy flux

**Fig. 2:**
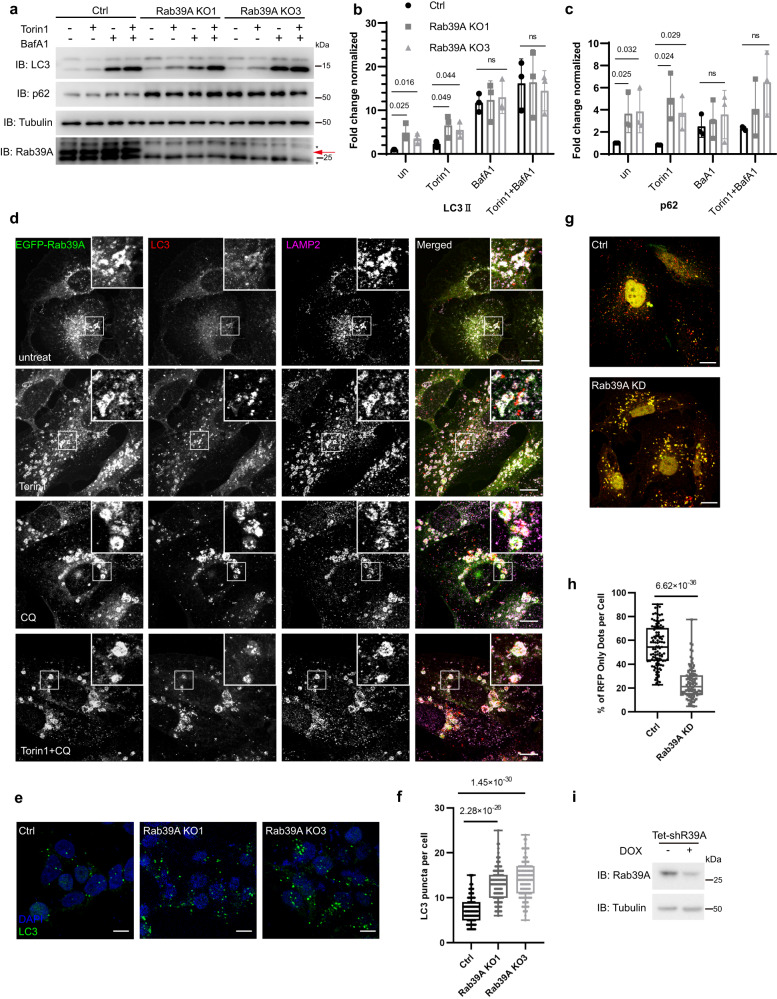
Rab39A promotes autophagosome-lysosome fusion. **a**–**c** Autophagy flux was inhibited in Rab39A KO 293 T cells compared with ctrl cells. Ctrl cells, Rab39A KO1 and Rab39A KO3 cells were treated with 50 nM Torin 1 with or without 100 nM Bafilomycin A1 for 3 h and then immunoblotting was performed with indicated antibodies. Red arrow indicates Rab39A band. *, non-specific band. **b**, **c** Quantitative results of (**a**). LC3II and p62 value of each sample is first divided by the corresponding Tubulin value of the sample to obtain the LC3II/Tubulin ratio and p62/Tubulin ratio, which are then normalized to control cell untreated sample. Data are mean ± SD from three independent repeats of experiments. Significance was determined by two-tailed *T* test between KO cells and control cells. *P* values are listed. ns, no significance. **d** EGFP-Rab39A residents on lysosomes and translocates to autolysosome upon autophagy induction. U_2_OS cells with inducible expression of EGFP-Rab39A were treated with 50 nM Torin 1 with or without 50 µM CQ for 3 h. Then cells were fixed and immune stained with antibodies against LAMP2 and LC3. Images were collected by confocal microscopy. Scale bar, 10 µm. White-framed squares show zoomed area. **e** LC3-positive autophagic vacuoles were accumulated in Rab39A KO cells compared with ctrl cells. Rab39A KO cells and ctrl cells were treated with 50 nM Torin 1 for 3 h. Then cells were fixed and immune stained with LC3 antibody. DAPI staining indicated nuclei. Images were obtained by fluorescence microscopy. Scale bar, 10 µm. **f** Quantification result of (**e**). Data are mean ± SD. *n* = 108 cells per group. Significance was determined by two-tailed *T* test between ctrl cells and KO cells. *P* values are listed. **g** Acidification of autophagosomes is inhibited in Rab39A KD cells. U_2_OS cells with inducible knockdown of Rab39A and stable expression of mRFP-GFP-LC3 were divided into ctrl or Rab39A KD group, by treated with or without Doxycycline. Both groups of cells were treated with 50 nM Torin 1 for 3 h before imaging GFP-RFP-LC3 fluorescence. Scale bar, 10 µm. **h** Quantification of the results in (**g**). *n* = 106 cells per group. Percentage of RFP^+^GFP^-^ dots in total RFP dots. Significance was determined by two-tailed *T* test between ctrl cells and KD cells. *P* value is listed. **i** Immunoblotting result of knockdown efficiency of Rab39A. DOX, doxycycline. The box extends from the 25th to 75th percentiles, and the whiskers go down to the smallest value and up to the largest. Source data are provided as a Source Data file.

To precisely dissect the function of Rab39A or Rab39B in autophagy, we knocked out Rab39A or Rab39B by CRISPR/Cas9 in 293T cells since 293T cells has relatively high expression level of these proteins, while U_2_OS cells which we used in this paper to observe the distribution of proteins express much lower levels of endogenous Rab39A and Rab39B. Knocking out Rab39A or Rab39B did not affect the expression levels of the other Rab39 isoform (Fig. S5a). The autophagy flux of two independent Rab39A KO cell lines is significantly reduced compared with control cells (Fig. 2a–c), indicated by accumulation of both LC3II and p62 in two independent KO cell lines no matter autophagy induction is employed or not. Regarding the cellular distribution, Rab39A majorly localizes on lysosomes under untreated condition (Fig. 2d). Once autophagy is induced, Rab39A is largely enriched on autophagosomes/autolysosomes (Fig. 2d and Fig. S5b). Rab39A did not localize on newly synthesized phagophore structures labeled by WIPI2 protein (Fig. S5c, d). Further, more LC3-positive puncta were accumulated in Rab39A KO cells (Fig. 2e, f), and the electronic microscopy images confirmed that double-membrane autophagosomes accumulated in Rab39A KO cells (Fig. S5e, f), compared with control cells. Besides, mRFP-GFP-LC3 conversion assay is used to examine autophagosome maturation since ectopic LC3 protein is tagged with acid-resistant red fluorescent protein (mRFP) and acid-sensitive green fluorescent protein (GFP). Through mRFP-GFP-LC3 conversion assay, we found much more autophagosomes kept neutral in Rab39A KD cells compared with control cells (Fig. 2g–i). These results indicate that Rab39A deficient cells exhibited impaired autophagosome-lysosome fusion phenotype. To exclude the possibility that lysosomes are defective in Rab39A KO cells, we analyzed lysosome quantity by electronic microscopy imaging (Fig. S5e, g), LysoTracker staining (Fig. S5h, i) and lysosome function by EGFR degradation assay (Fig. S5j, k). The number of lysosomes shown in TEM images or acidic dye LysoTracker staining puncta in confocal images of Rab39A KO cell lines was similar to those in control cells. Also, the degradation rate of EGF receptor by lysosome in Rab39A KO cells is similar to those in control cells. Conclusively, the above results suggest that Rab39A regulates autophagy by affecting autophagosome-lysosome fusion. Meanwhile, we explored the role of Rab39B in autophagy in parallel experiments. Rab39B KO cells exhibited slightly impaired autophagy flux, including decreased LC3 level upon autophagy induction and unchanged p62 level (Fig. S6a–c). Partial Rab39B colocalized with LC3- or LAMP2- positive vesicles (Fig. S6d). Unlike Rab39A, Rab39B was found on the WIPI2-labeled phagophore structures (Fig. S5d and S6e). Besides, quantity of autophagosomes and autolysosome/lysosomes decreased in Rab39B KO cells upon autophagy induction (Fig. S5e–g, Fig. S6f–i). These data suggested that Rab39B may affect autophagosome or lysosome quantity, and has a weak effect on autophagy flux^31^.

In summary, although Rab39A and Rab39B share more than 77% identity of amino acid sequence and similar binding behavior with HOPS complex subunits, their function in autophagy is obviously different. Rab39A mediates autophagosome-lysosome fusion, while Rab39B may be involved in autophagosome formation and has a mild effect on autophagy flux. We postulate that Rab39A likely participates in autophagosome-lysosome fusion through its direct interaction with the HOPS complex.

### Rab39A promote the assembly of autophagic tethering and fusion machinery

**Fig. 3:**
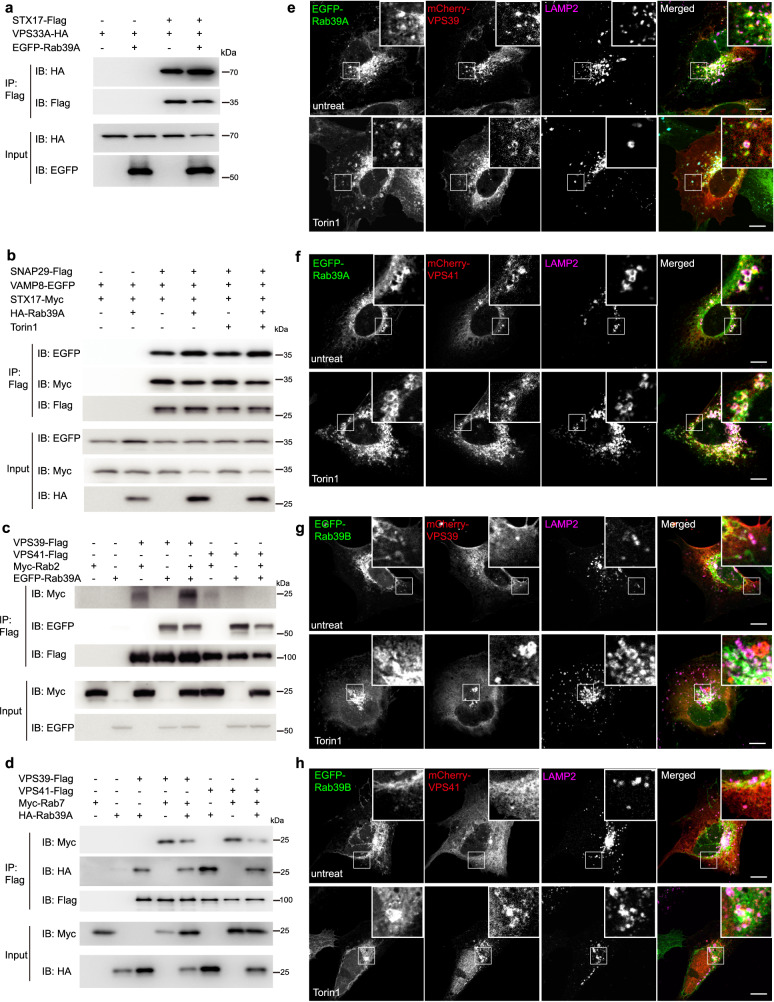
Rab39A enhances assembly of autophagic fusion machinery. **a** Rab39A promotes interaction between STX17 and HOPS complex subunits. 293T cells were transfected with vector or tagged protein expression plasmid as indicated. 24 h after transfection, cells were lysed and immunoprecipitated with Flag M2 agarose. Immunoblotting was performed with indicated antibodies. **b** Rab39A promotes assembly of autophagic SNAREs. 293T cells were transfected with vector or tagged protein expression plasmids as indicated. Before harvest, indicated groups of cells were treated with 50 nM Torin 1 for 3 h. All transfected cells were harvested 24 h after transfection, then lysed and immunoprecipitated with Flag M2 agarose. Immunoblotting was performed with indicated antibodies. **c** Rab39A enhances Rab2 binding with VPS39. 293T cells were transfected with vector or tagged protein expression plasmids as indicated. 24 h after transfection, cells were lysed and immunoprecipitated with Flag M2 agarose. Immunoblotting was performed with indicated antibodies. **d** Rab39A displaced Rab7 from binding to HOPS complex. 293T cells were transfected with vector or tagged protein expression plasmids as indicated. 24 h after transfection, cells were lysed and immunoprecipitated with Flag M2 agarose. Immunoblotting was performed with indicated antibodies. **e**, **f** Rab39A co-localized with HOPS complex subunits VPS39 (**e**) and VPS41 (**f**). Stable U_2_OS cell line with inducible expression of EGFP-Rab39A was transfected with mCherry-VPS39 or mCherry-VPS41 expression plasmids. 24 h after infection, cells were untreated or treated with 50 nM Torin 1 for 3 h. Then cells were fixed and immunostained with anti-LAMP2 antibody and observed under confocal microscopy. Scale bar, 10 µm. White-framed squares show the zoomed area. **g**, **h** Rab39B partially co-localized with HOPS complex subunits VPS39 (**g**) and VPS41 (**h**). Stable U_2_OS cell lines inducibly expressing EGFP-Rab39B were infected with lenti-virus expressing mCherry-VPS39 or mCherry-VPS41. 24 h after infection, cells were untreated or treated with 50 nM Torin 1 for 3 h. Then cells were fixed and immunostained with anti-LAMP2 antibody and observed under confocal microscopy. Scale bar, 10 µm. White-framed squares show the zoomed area. Source data are provided as a Source Data file.

The Vps33 subunit of yeast HOPS complex has been shown to template vacuolar SNAREs assembly^6^. Mammalian homolog VPS33A has been shown to interact with STX17 directly^32^ and stabilize autophagic SNARE bundle^32^. Thus, we detected whether the overexpression of Rab39A would affect the interaction between STX17 and VPS33A, as well as the mammalian autophagic SNAREs assembly. In 293T cells with overexpressed Rab39A, STX17 immunoprecipitated with more VPS33A (Fig. 3a and Fig. S7a), which suggested Rab39A promoted interaction between VPS33A and STX17. In addition, with overexpression of Rab39A, SNAP29 immunoprecipitated with more VAMP8, but not with more STX17 (Fig. 3b and Fig. S7b), indicating overproduced Rab39A didn’t promote STX17–SNAP29 binary t-SNARE complex assembly, but it stabilized STX17–SNAP29–VAMP8 ternary complex zippering.

Since we observed that Rab39A directly interacts with VPS39 and VPS41 (Fig. 1h and Fig. S4b), we wonder whether these interactions would affect the binding of other Rab GTPases with HOPS subunits. Intriguingly, we found that Rab2, which exhibits a direct binding to VPS39 but not VPS41 (Fig. 1a), interacted with more VPS39 when Rab39A was overproduced (Fig. 3c and Fig. S7c). As for Rab7, which showed interaction with both VPS39 and VPS41 (Fig. 1b), co-expression of HA-Rab39A displaced Myc-Rab7 from binding with both VPS39 and VPS41 (Fig. 3d and Fig. S7d). These data suggested the existence of Rab39A promoted the interaction between Rab2 and VPS39, but not Rab7 to VPS39. On the other hand, Rab39A might compete with and prevent Rab7 from binding with VPS41 in the presence of overproduced Rab39A.

Furthermore, we found that upon autophagy induction, VPS39 and VPS41 resided on lysosomes or autolysosomes stained by LAMP2, and co-localized well with Rab39A, but less with Rab39B (Fig. 3e–h). These results are consistent with our previous finding (Fig. 2) that Rab39A is more likely to mediate autophagosome-lysosome fusion.

Taken together, Rab39A directly binds to the tip subunit VPS41 of the HOPS complex, and this interaction prevented Rab7 from binding with VPS41 and enhances binding between VPS39 and Rab2, therefore promotes the assembly of autophagic the Rabs-HOPS complex. Since Rab2-HOPS-Rab7 failed to promote autophagic SNAREs driven lipid mixing in vitro (Fig. 1d, e), it is possible that Rab39A plays a crucial role in HOPS recruitment and autophagosome-lysosome fusion.

### Rab2-HOPS-Rab39A promotes autophagic SNAREs driven membrane fusion in vitro

We then set up the reconstituted fusion system containing human HOPS complex, Rab2 incorporated STX17-SNAP29-T liposome and Rab39A incorporated VAMP8-V liposome recapitulating autophagosome-lysosome fusion in vivo. Rab2 was incorporated into T liposome mimicking autophagosomes given the localization of Rab2 on autophagosomes and its direct binding to the tip subunit VPS39 of the HOPS complex (Fig. 1a). Rab39A was incorporated into V liposome mimicking lysosomes given the localization of Rab39A on lysosomes and its direct binding to the tip subunit VPS41 of the HOPS complex (Fig. 1h and Fig. S4b). In this in vitro system, we aimed to investigate if the membrane-anchored Rab2-HOPS-Rab39A complex could promote the fusogenic activity of autophagic SNAREs using proteoliposomes ensemble lipid- and content-mixing assays.

**Fig. 4:**
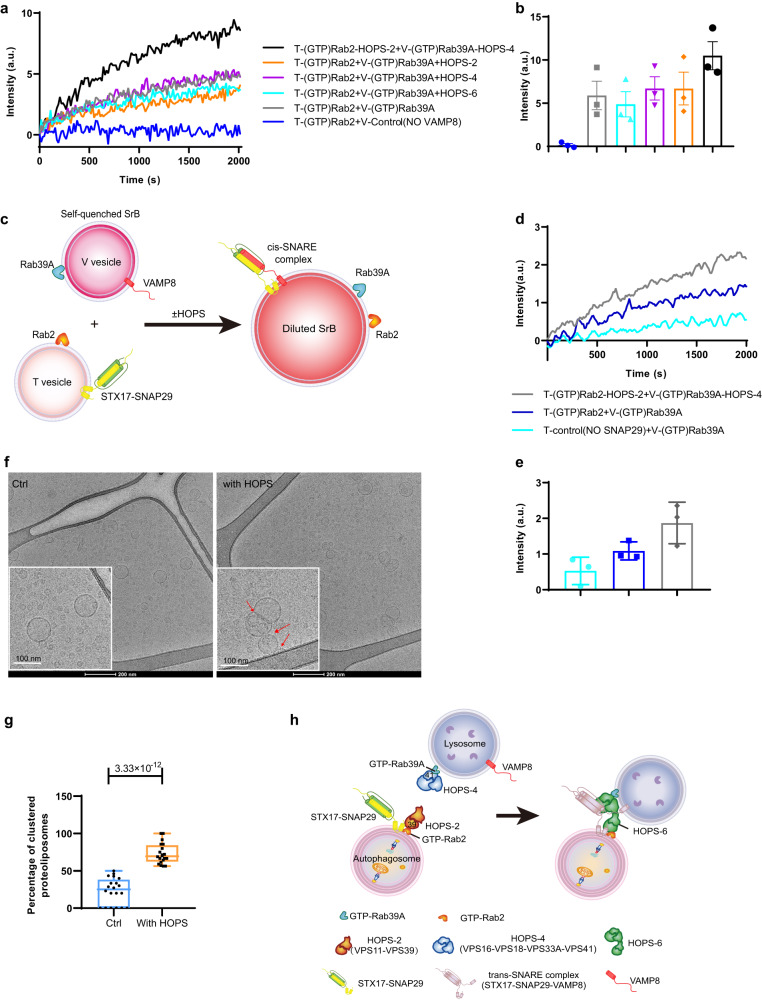
Rab39A-HOPS-Rab2 bridges autophagic vesicles and enhances fusion in vitro. **a** Rab39A-HOPS-Rab2 promotes lipid mixing of proteoliposomes reconstituted with autophagic SNAREs. HOPS-2, VPS39/VPS11 subcomplex of HOPS complex; HOPS-4, VPS16/VPS18/VPS33A/VPS41 subcomplex of HOPS complex; HOPS-6, pre-incubated VPS39/VPS11 subcomplex and VPS16/VPS18/VPS33A/VPS41 subcomplex of HOPS complex. T, proteoliposomes incorporated with t-SNAREs (STX17-SNAP29); V, proteoliposomes incorporated with v-SNARE (VAMP8); a.u., arbitrary units. **b** Quantitative of results of (**a**) at around 2000 sec from three independent replicated experiments. a.u., arbitrary units. Data are presented as mean values ± SEM. **c** Schematic drawing of content mixing assay. **d** Rab39A-HOPS-Rab2 promotes content-mixing of proteoliposomes reconstituted with autophagic SNAREs. HOPS-2, two-subunit HOPS subcomplex containing VPS39/VPS11; HOPS-4, four-subunit HOPS subcomplex containing VPS16/VPS18/VPS33A/VPS41 T, proteoliposomes incorporated with t-SNAREs (STX17-SNAP29); V, proteoliposomes incorporated with v-SNARE (VAMP8); a.u., arbitrary units. **e** Quantitative of results of (**d**) at around 2000 sec from three independent replicated experiments. a.u., arbitrary units. Data are presented as mean values ± SEM. **f** Representative Cryo-EM images of mixed proteoliposomes incorporated with Rab2 or Rab39A separately (Ctrl); or proteoliposomes incorporated with Rab2 preincubated with HOPS-2 and proteoliposomes incorporated with Rab39A preincubated with HOPS-4 (with HOPS). Enlarged images were shown in the white-framed squares. Red arrows indicate tethered proteoliposomes. Scale bars for large images, 200 nm. Scale bars for enlarged images, 100 nm. **g** Percentage of tethered proteoliposomes per 1.69 μm² observed area as captured by electron microscopy (*n* = 20). Data are analyzed by two-sided *T* test and are presented as mean values ± SD. The box extends from the 25th to 75th percentiles, and the whiskers go down to the smallest value and up to the largest. **h** Schematic model illustrates how the HOPS complex bridges autophagosome and lysosomes by a “hook-up” way. GTP-loaded Rab39A is enriched on the lysosomes, and subsequently recruits the HOPS-4 subcomplex. On the other hand, autophagosomal localized GTP-Rab2 recruits the HOPS-2 subcomplex. HOPS-2 and HOPS-4 “hook-up” to form HOPS-6 to tether autophagosome and lysosome, which leads to promote autophagosome-lysosome fusion driven by STX17-SNAP29-VAMP8. HOPS-6, HOPS complex containing VPS11, VPS16, VPS18, VPS33A, VPS39 and VPS41; V39, VPS39; V41, VPS41. Source data are provided as a Source Data file.

For lipid mixing assay, the proteoliposomes were reconstituted and activated as previously described, except that the VAMP8 liposomes were incorporated with Rab39A instead of Rab7, hereinafter referred to as V-Rab39A. The lipid mixing measured by increased DiI-DiD FRET signals was observed between T-Rab2-GTP and V-Rab39A-GTP in the presence of autophagic SNAREs (Fig. 4a, b, gray). Upon removal of VAMP8 from the V-Rab39A liposomes, the FRET was not increased (Fig. 4a, b, blue). This suggests that the lipid mixing observed between T-Rab2-GTP and V-Rab39A-GTP depends on SNARE complex formation. Next, we added HOPS sub-complexes into the reconstitution system to check whether assembly of HOPS can promote lipid mixing. We found if T-Rab2-GTP and V-Rab39A-GTP were pre-incubated with two-subunit HOPS (VPS11/VPS39) subcomplex and four-subunit HOPS (VPS18/VPS16/VPS33A/VPS41) subcomplex respectively, the lipid mixing was enhanced (Fig. 4a, b, black). Besides, upon omitting two-subunit HOPS subcomplex (Fig. 4a, b, orange) or four-subunit HOPS subcomplex (Fig. 4a, b, purple), this enhanced lipid mixing was reversed. These results suggest that both membrane-associated two-subunit HOPS subcomplex and four-subunit HOPS subcomplex are required for forming a functional HOPS complex to promote lipid mixing. Interestingly, we observed that pre-assembled six-subunit HOPS complex in solution is not able to promote the lipid mixing between T-Rab2-GTP and V-Rab39A-GTP liposomes (Fig. 4a, b, cyan). This suggest assembling between vesicles incorporated with particular Rab GTPase pairs might be necessary for two membrane-associated HOPS subcomplexes to form a right conformation to be “hooked up” to assemble into a functional protein complex to promote membrane tethering.

Meanwhile, the ensemble content mixing assay with sulphorhodamine B (SrB) used in our previous studies^33^ was applied to confirm HOPS complex assembled between T-Rab2 and V-Rab39A can promote their membrane fusion. The proteoliposomes were reconstituted similarly as in lipid mixing assay, except that the DiI and DiD lipids were removed from the liposomes and replaced by the content marker SrB with high concentration, encapsulated in the V-Rab39A liposomes. The content mixing was measured from the fluorescence de-quenching of SrB (Fig. 4c). Consistent with the lipid mixing assay, we observed content mixing between T-Rab2-GTP and V-Rab39A-GTP (Fig. 4d, e, blue) and this content mixing depended on the SNARE complex formation (Fig. 4d, e, cyan). Besides, the pre-incubation of T-Rab2-GTP and V-Rab39A-GTP with two-subunit HOPS subcomplex and four-subunit HOPS subcomplex respectively can promote content mixing between T-Rab2-GTP and V-Rab39A-GTP (Fig. 4d, e, gray). All these results further support the “hooking up” model for the HOPS complex assembly between T-Rab2-GTP and V-Rab39A-GTP to form a functional tethering complex for SNARE mediated membrane fusion.

Next, we used cryo-EM to further confirm whether the addition of both two-subunit HOPS subcomplex and four-subunit HOPS subcomplex is able to tether Rab2-incorporated liposomes and Rab39A-incorporated liposomes. The liposomes were reconstituted as in the lipid mixing assay except no SNAREs and no fluorescence dye are included. Without the addition of the HOPS sub-complexes, Rab2-liposomes and Rab39A-liposomes distributed homogeneously in the ice layer. On the contrary, pre-incubation of Rab2-liposomes and Rab39A-liposomes with two-subunit HOPS subcomplex and four-subunit HOPS subcomplex respectively, led to the clustering of liposomes (Fig. 4f, g).

All these results suggest that the assembly of the HOPS complex between Rab2-incorporated proteoliposomes and Rab39A-incorporated proteoliposomes can form a functional complex to tether vesicles and promote the membrane fusion driven by autophagic SNAREs (Fig. 4h).

### GTP loading and C-terminal prenylation are crucial for Rab39A to recruit the HOPS complex onto autolysosomes

It is well accepted that Rab GTPases localized to intracellular membranes via post-translationally attached geranylgeranyl lipids on their C-terminals. Switching from GDP loading form to GTP loading form is critical for Rabs to bind with effector proteins. Rabs are stabilized on membranes after activation and effector binding^13,34–36^. We then tested whether the activity change and lipidation modification of Rab39A would affect its intracellular distribution and its function in autophagy. First, we found the constitutively active mutant Rab39A-Q72L localized on autolysosomes stained by both LC3 and LAMP2 in cells upon Torin 1 treatment, similar to Rab39A-WT (Fig. S8a). On the contrary, the constitutively inactive mutant Rab39A-S22N exhibited cytosolic distribution in cells even under autophagy induction condition, similar to non-prenylatable mutant Rab39A-C215A/C217A (mentioned as CC/AA hereafter), where both carboxyl-terminal cysteines were replaced by alanine residues (Fig. S8a). These suggest both prenylation and GTP loading are important for Rab39A to target on membranes.

**Fig. 5:**
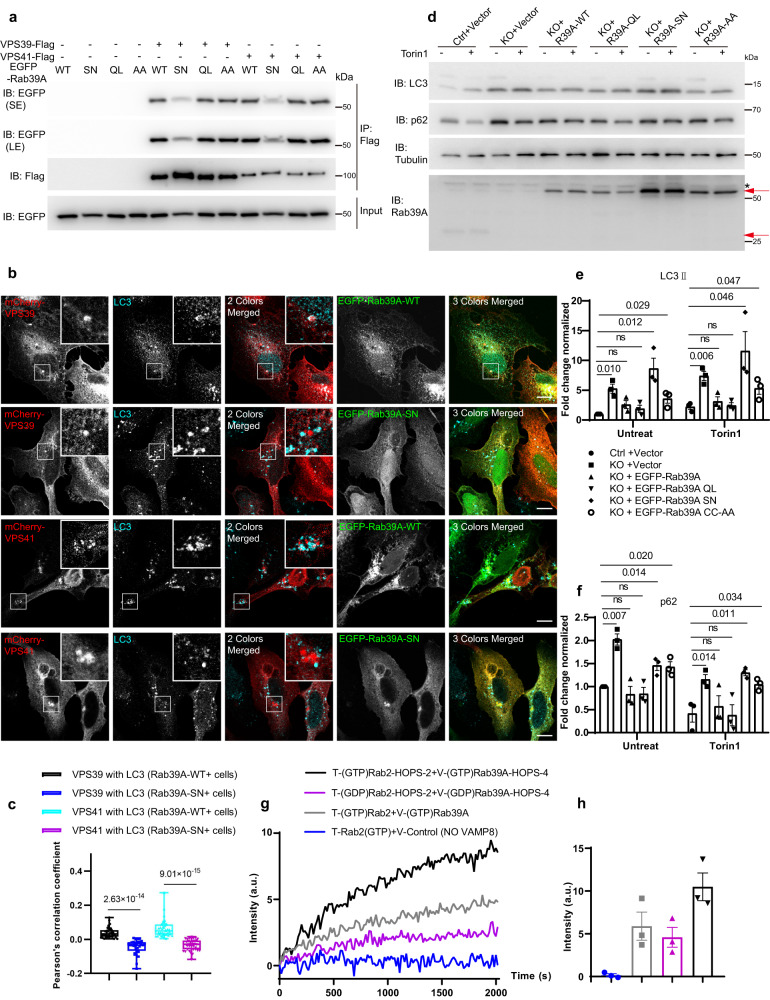
Rab39A is required for autophagy depends on its GTP loading and C-terminal prenylation. **a** Rab39A binding with HOPS complex depends on its GTP loading, but not C-terminal prenylation. 293T cells were transfected with indicated plasmids for 24 h. Cells were lysed and immunoprecipitated with Flag M2 agarose and then Immunoblotted with indicated antibodies. SE, short exposure; LE, long exposure. **b** Activation of Rab39A enables HOPS complex subunits recruited onto autophagic vesicles. U_2_OS cells were transfected with indicated plasmids. After treated with 50 nM Torin 1 for 3 h, cells were fixed and immune stained with indicated antibody. White-framed squares show the zoomed area. Scale bar, 10 µm. **c** Quantification of results of (**b**). Each point represents an image used for colocalization analysis, *n* = 37, 33, 39, 37, containing 101, 103, 102, 105 cells respectively. Significance was determined by two-tailed *T* test. *P* value is listed. The box extends from the 25th to 75th percentiles, and the whiskers go down to the smallest value and up to the largest. **d** Rab39A regulates autophagy flux depending on its GTP loading activation and C-terminal prenylation. Cells were transfected with indicated plasmids respectively for 24 h. After treated with or without 50 nM Torin 1 for 3 h, cell lysate was immunoblotted with indicated antibodies. *, non-specific band; red arrows indicated endogenous Rab39A (lower) or EGFP-Rab39A (upper). **e**, **f** Quantification of results of (**d**) LC3II levels (**e**) or p62 levels (**f**) from three independent experiments. LC3II and p62 value are first divided by the corresponding Tubulin value of the sample to obtain the LC3II/Tubulin ratio and p62/Tubulin ratio, which are then normalized to lane 1 sample. Data are mean ± SEM. Significance is determined by two-tailed T test P value is listed. ns, not significant. **g** Rab2-HOPS-Rab39A promoting lipid mixing of proteoliposomes requires GTP loading activation. HOPS-2, VPS39/VPS11 subcomplex of HOPS complex; HOPS-4, VPS16/VPS18/VPS33A/VPS41 subcomplex of HOPS complex. T, proteoliposomes incorporated with t-SNAREs (STX17-SNAP29); V, proteoliposomes incorporated with v-SNARE (VAMP8); a.u., arbitrary units. **h** Quantitative results of (**g**) at around 2000 sec from three independent replicated experiments. a.u., arbitrary units. Data are presented as mean ± SEM. Source data are provided as a Source Data file.

Second, we investigated whether prenylation and activation would affect Rab39A binding to HOPS. As expected, both VPS39 and VPS41 immunoprecipitated with much less Rab39A-SN mutant compared to Rab39A-WT (Fig. 5a and Fig. S8b). This indicates that the GTP loading is critical for Rab39A to interact with its effector HOPS. Both VPS39 and VPS41 still bound to the Rab39A non-prenylatable CC/AA mutant (Fig. 5a and Fig. S8b), probably because the Rab39A CC/AA mutant only affects its membrane association but not the GTP loading of Rab39A. Furthermore, we tested whether the recruitment of HOPS subunits to autophagic membrane structures under autophagy- induced condition is dependent on the activation of Rab39A. The results showed that once ectopic Rab39A-SN was expressed, both VPS39 and VPS41 showed declined autolysosome distribution (Fig. 5b, c). These data further proved that HOPS is the effector of Rab39A. Besides, it is worth noting that overexpressing the SN form of Rab39B did not affect HOPS subunits localization on autophagic vesicles (Fig. S8c, d), which is consistent with previous observation that Rab39B doesn’t function in autophagosome-lysosome fusion.

Next, we investigated the effect of GTP loading or prenylation of Rab39A in autophagy by examining the autophagy flux in Rab39A KO cells complemented with different Rab39A mutants. As expected, Rab39A-SN and Rab39A-CC/AA cannot rescue the autophagy defect in Rab39A KO cells (Fig. 5d–f), while WT or QL form of Rab39A could restore the autophagy flux in Rab39A depleted cells (Fig. 5d–f).

Finally, we tested whether the promoted membrane fusion by HOPS and Rab GTPases is dependent on the activation of Rab39A. The lipid mixing assay showed that only GTP-loaded Rab2 and Rab39A could enable HOPS to enhance the autophagic SNAREs mediated lipid mixing in vitro, while the GDP-loading of Rab GTPases failed to do so (Fig. 5g, h).

Taken together, these findings revealed that both prenylation and GTP loading of Rab39A are important for its subcellular localization, recruitment of HOPS to autophagic vesicles, and its function in regulating autophagy flux.

### C9orf72 functions as Guanine Nucleotide Exchange Factor (GEF) of Rab39A to play a role in autophagosome-lysosome fusion

**Fig. 6:**
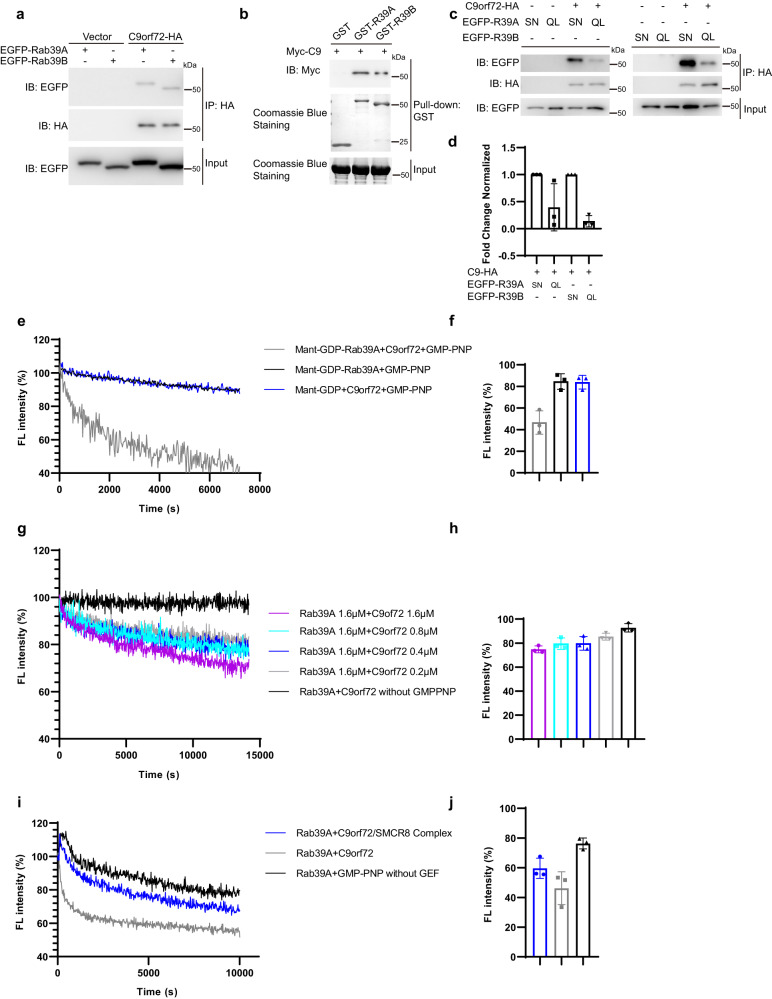
C9orf72 is a guanine nucleotide exchange factor (GEF) of Rab39A in autophagy. **a** Rab39A and Rab39B both interact with C9orf72 in vivo. 293T cells were transfected with vector or tagged protein expression plasmids as indicated. Cells were harvested 24 h after transfection, then lysed and immunoprecipitated with HA agarose. Immunoblotting was performed with indicated antibodies. **b** C9orf72 directly interacts with Rab39A and Rab39B in vitro. Glutathione beads were blocked with BSA and conjugated with GST or GST-Rab39A (GST-R39A) or GST-Rab39B (GST-R39B), then conjugated Glutathione beads were incubated with purified Myc-C9orf72 (Myc-C9) respectively, and then washed and boiled for immunoblotting or Coomassie blue staining. **c** GDP-loading form of Rab39A or Rab39B increases binding between C9orf72 and Rab39A or Rab39B. 293T cells were transfected with vector or tagged protein expression plasmids as indicated. Cells were harvest 24 h after transfection, then lysed and immunoprecipitated with HA agarose. Immunoblotting was performed with indicated antibodies. R39A, Rab39A; R39B, Rab39B. **d** Quantification results of (**c**) from three independent repeats. Fold change normalized represents the ratio of immunoprecipitated EGFP-Rab39A/B SN or QL band intensity to C9orf72-HA band intensity. The QL Lane was normalized to SN lane. Data are mean ± SD. C9, C9orf72. **e** In vitro GEF assays showed GEF activity of C9orf72 on Rab39A using an equimolar protein ratio between the two. Nucleotide exchange activity of C9orf72 was monitored by measuring the fluorescent signal of released Mant-GDP from Rab39A. One representative plot from three independent experiments is shown for each sample. **f** Quantitative results of (**e**) at 7200 s from three independent replicated experiments. Data are mean ± SD. **g** Nucleotide exchange was monitored by titration with different concentrations of C9orF72 (0.2 µM-1.6 µM) to 1.6 µM Rab39A and 100 µM GMP-PNP. One representative plot from three independent experiments is shown for each sample. **h** Quantitative of results of (**g**) at 14184.5 s from three independent experiments. Data are mean ± SD. **i** C9orf72 protein alone, not C9orf72/SMCR8 complex catalyzed GTP loading of Rab39A. One representative plot from three independent experiments is shown for each sample. **j** Quantitative results of (**i**) at 10002.4 s from three independent experiments. Data are mean ± SD. Source data are provided as a Source Data file.

It is well known that GDP/GTP exchange of small GTPase is catalyzed by guanine nucleotide exchange factor (GEF)^37^. To better understand the upstream regulating mechanism of Rab39A in promoting autophagosome-lysosome fusion, we then investigated the potential GEF stimulating GTP loading of Rab39A to activate this GTPase. As the high identity of amino acid sequence between Rab39A and Rab39B, we hypothesized that the reported GEF of Rab39B, C9orf72^38^ could be a promising candidate. On contrast to the previous study^38^, we found C9orf72 protein binds to Rab39A and Rab39B in cells (Fig. 6a). Besides, in vitro pull-down assay showed that C9orf72 protein could directly interact with both Rab39A and Rab39B (Fig. 6b), and GDP-bound forms of Rab39A and Rab39B enhanced their binding with C9orf72 (Fig. 6c, d). To verify the in vitro GEF activity of C9orf72 for Rab39A, we employed N-Methylanthraniloyl (Mant) labeled guanine nucleotide to monitor nucleotide exchange of Rab39A. The emitted fluorescent signal of Mant-labeled guanine nucleotide increases upon binding to a GTPase (typically twice as high as the signal of unbound MANT-guanine nucleotide)^39^, enabling direct monitoring of the association and dissociation of guanine nucleotides from GTPases. Upon GEF activation, the Rab GTPase-bound Mant-GDP will be exchanged to non-hydrolysable GTP analog GMP-PNP, resulting in the release of Mant-GDP. Thus the GDP/GTP exchange rate catalyzed by GEF can be kinetically monitored by the decreasing fluorescent signal of Mant-GDP. By this Mant-GDP releasing assay, we found that C9orf72 strongly facilitated the release of Mant-GDP from Rab39A when the ratio of these two proteins is 1:1 (Fig. 6e, f), and the GEF efficiency of C9orf72 for Rab39A is dependent on its concentration (Fig. 6g, h). In the previous study, C9orf72 together with SMCR8 and WDR41 could form a C9orf72-SMCR8-WDR41 complex function as a GAP for Rab8A and Rab11A^40^, or as GEF complex for Rab8A and Rab39B^41^. Inside the C9orf72-SMCR8-WDR41 complex, both C9orf72 and SMCR8 harbor DENN (Differentially Expressed in Normal and Neoplastic cells) domains, which confer the GDP/GTP exchange function to these two proteins, while WDR41 has no such catalytic domain and may play an unknown role in this complex^42,43^. Thus, we wondered if the C9orf72-SMCR8 complex would increase the GEF activity toward Rab39A comparing with C9orf72 protein alone. To our surprise, the result showed that C9orf72 protein alone was more efficient than the C9orf72-SMCR8 complex (Fig. 6i, j). These data indicated Rab39A may have a GTP loading mechanism via C9orf72 distinct from Rab39B.

**Fig. 7:**
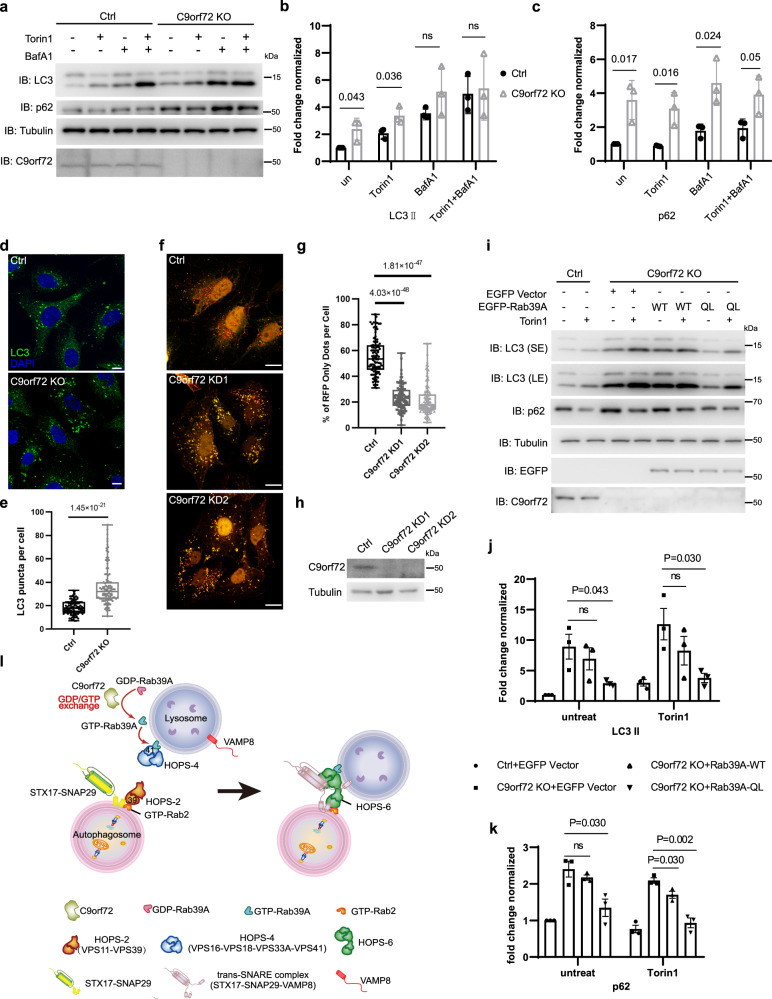
C9orf72 activates Rab39A to support autophagosome-lysosome fusion. **a** Autophagy flux was inhibited in C9orf72 KO U_2_OS cells. Cells were treated with 50 nM Torin 1 with or without 100 nM Bafilomycin A1 for 3 h and then immunoblotted with indicated antibodies. **b**, **c** Statistic results of (**a**) indicating LC3II (**b**) and p62 (**c**) levels from three independent experiments. Data are mean ± SD. The LC3II/Tubulin ratio and p62/Tubulin ratio are normalized to lane 1 sample. *P* values are obtained from two-tailed *T* test. ns, not significant. **d** LC3-positive autophagic vesicles were accumulated in C9orf72 KO cells. Cells were treated with 50 nM Torin 1 for 3 h, then fixed and immune stained with LC3 antibody. DAPI staining indicated nuclei. Scale bar, 10 µm. **e** Quantification result of (**d**). Data are mean ± SD. *n* = 108 cells per group. *P* values are obtained from two-tailed *T* test. **f** Acidification of autophagosomes was inhibited in C9orf72 KD cells. U_2_OS cells stably expressing RFP-GFP-LC3 were transfected with indicated SiRNA for 48 h and then treated with 50 nM Torin 1 for 3 h before imaging Scale bar, 10 µm. **g** Quantification of results in (**f**). *n* = 106 cells per group. Percentage of RFP^+^GFP^-^ dots in total RFP dots. *P* values are obtained from two-tailed T test. **h** Immunoblotting results show the efficiency of C9orf72 knockdown in (**f**). **i** Autophagy defect is rescued by Rab39A-QL expression in C9orf72 KO cells. Cells were infected with indicated expression lenti-virus. Cells were treated with or without 50 nM Torin 1 for 3 h and then immunoblotted with indicated antibodies. **j**, **k** Statistic results of (**i**) indicating LC3II (**j**) and p62 (**k**) levels from three independent experiments. Data are mean ± SEM. The LC3II/Tubulin ratio and p62/Tubulin ratio are normalized to lane 1 sample. *P* values are obtained from two-tailed *T* test ns, not significant. **l** Schematic model illustrates how C9orf72-Rab39A-HOPS promotes autophagosome-lysosome fusion. The box extends from the 25th to 75th percentiles, and the whiskers go down to the smallest value and up to the largest. Source data are provided as a Source Data file.

Next, we asked if C9orf72 played a role in autophagosome-lysosome fusion through Rab39A. There are variant studies of C9orf72 function in autophagy and the conclusions are inconsistent^41,44–49^. Most of the studies discussed functions of C9orf72 in neuron system^41,44,45,49^, and the underlying mechanism is elusive. Here, we established C9orf72 knockout U_2_OS cell lines by CRISPR/Cas9 genome-editing method to precisely examine the function of C9orf72 in autophagic membrane fusion (Fig. 7a). C9orf72 KO cells exhibited inhibited autophagy flux (Fig.7a–c) and accumulated LC3-positive autophagic vesicles (Fig. 7d, e). Knocking down of C9orf72 in U_2_OS cells also led to impaired autophagy flux shown by mRFP-GFP-LC3 conversion assay (Fig. 7f–h). As for lysosome activity, C9orf72 KO cells showed unchanged EGFR-degradation activity (Fig. S9a and b) and unaffected lysosome morphology and quantity (Fig. S9c–g). Moreover, upon autophagy induction, a fair portion of C9orf72 was gathered on autolysosome structures, whereas C9orf72 mainly diffused in cytosolic without autophagy induction or degradation blockage (Fig. S9h, i). These data support a role of C9orf72 in autophagosome-lysosome fusion without affecting lysosome activities, and the phenotypes were well copied in Rab39A KO or KD cells. With C9orf72 depletion, the GTP loading activation of Rab39A is abolished, indicated by WT Rab39A lost its distribution on autolysosome, but not the constitutively activated form Rab39A-QL (Fig. S10, b). Also, HOPS complex subunits recruitment onto autophagic vesicles is largely impaired in C9orf72 KO cells (Fig. S10c, d). Finally, when we complemented Rab39A-QL into C9orf72 KO cells, the autophagy defect is reversed (Fig. 7i–k), but this alteration cannot be observed in Rab39A-WT or empty vector expressed groups (Fig. 7i–k). These data revealed that C9orf72 depletion caused defect in Rab39A activation, subsequent HOPS complex recruitment on autolysosome and autophagosome-lysosome fusion and uncovered the function axis of C9orf72-Rab39A-HOPS complex in autophagosome-lysosome fusion.

## Discussion

In this study, we reconstituted autophagosome-lysosome fusion in vitro with autophagic SNAREs, assembled HOPS complex, Rab2 and Rab39A. We revealed Rab39A as a critical Rab GTPase in regulating autophagosome-lysosome fusion via recruitment of the HOPS complex onto lysosomes. Assembling HOPS between T-Rab2 and V-Rab39A led to a functional complex, which was able to promote membrane fusion driven by STX17-SNAP29-VAMP8. We recapitulated the vesicle tethering function of HOPS, where the two opposite subunits of HOPS, VPS39, and VPS41, bound to activated Rab2 and Rab39A from independent vesicles to cluster the liposomes. We also identified C9orf72 as a GEF for Rab39A activation and its role in mediating autophagosome-lysosome fusion via Rab39A. Taken together, we describe a functional axis, C9orf72-Rab39A-HOPS, in autophagosome-lysosome fusion (Fig. 7l).

The yeast HOPS complex is reported to operate as a hexamer. We found that mammalian HOPS complex functions differently. Biochemical reconstitution of human HOPS function was not previously reported, partially due to the difficulty to purify the functional human HOPS complex in vitro. We found that purification of human HOPS from 293 S cells with overexpression of all six subunits via affinity purification tags on Vps41 or VPS39 resulted in four-subunit HOPS or two-subunit HOPS (Fig. S1a–g). This is consistent with previous co-IP studies by another group, where the pulldown of the HOPS six-subunit holo-complex was barely detected (van der Kant et al., 2015). Our SEC analysis of HOPS from cell lysate also suggested that the six-subunit HOPS complex is not assembled spontaneously in cells (Fig. S1h). We were able to assemble the “six-subunit HOPS” complex by incubating two-subunit HOPS and four-subunit HOPS in solution (Fig. S1i–l), however, this pre-assembled HOPS in solution failed to promote membrane fusion driven by autophagic SNAREs (Fig. 4a, b). Instead, we found assembling HOPS complex between two proteoliposomes with proper Rab GTPases pair (Rab2 and Rab39A) were able to promote the membrane fusion (Fig. 4a–e). This indicates that the functional HOPS tethering complex could only be successfully assembled with appropriate Rabs and membranes. This notion was further supported by our cryo EM results, where assembled HOPS can cluster liposomes decorated with activated Rab2 and Rab39A. We propose a “hook-up” model for the membrane tethering activity of the HOPS complex, in which two HOPS subcomplexes (VPS39-VPS11, and VPS18-VPS16-VPS33A-VPS41) are recruited to different membranes via Rab2 or Rab39A respectively, and the membrane anchored HOPS subcomplexes (likely undergo conformational change) hook up together to be assembled into a functional complex to tether vesicles and promote membrane fusion. The “hook-up” HOPS-6 assembled between liposomes might adopt a distinct conformation compared to the randomly assembled HOPS-6 in solution. The liposome-associated conformation is a fusion favorable conformation with exposed binding sties/surfaces for autophagic SNAREs to dock and zipper properly, facilitating membrane fusion. Future cryo-EM/crystal structure analysis might help to resolve the topology of the six-subunit HOPS complex and the structure of Rab-binding HOPS between membranes.

Yeast HOPS uses its two subunits at opposite sites, Vps41 and Vps39, to bind with the vacuolar Rab GTPase Ypt7 to facilitate membrane tethering and fusion. In mammalian cells, the HOPS complex bridges membranes in a more complicated way. In human cells HOPS was recruited to autophagosome by Rab2 through VPS39 binding with Rab2^22,50^. It was reported that HOPS can be recruited to Rab7 attached lysosomes through the binding between Vps41 and Rab7 effector protein PLEKHM1 (Pleckstrin homology domain-containing protein) for autophagic fusion^24^ or the binding between Vps39 and another Rab7 effector protein RILP (Rab Interacting Lysosomal Protein) for endocytic trafficking^22^. Additionally, Arf-like GTPase 8b (Arl8b) is also found to recruit HOPS complex to lysosomes in mammalian cells, which has been implied to function in late endosome/lysosome transportation and lysosome fusion events^51,52^. In our study, we found both mammalian VPS39 and VPS41 can directly bind to Rab7 in pull-down assay (Fig. 1b). This is also supported by a recent published paper, where GST-Rab7 can pull-down with mammalian VPS41^53^. However, assembling the HOPS complex between GTP-loaded T-Rab2 and V-Rab7 is not able to promote the lipid mixing between proteoliposomes (Fig.1d, e). It is likely Rab7 is involved in autophagosome-lysosome fusion indirectly, probably through its effectors or functions in endolysosomes. In this study, we propose that, Rab39A instead of Rab7, is the appropriate small GTPase to recruit HOPS and promote autophagosome and lysosome fusion. We found that overexpressed Rab39A could displace Rab7 from direct binding of VPS41 (Fig. 3d and Fig. S7d) and promote interaction between Rab2 and VPS39 (Fig. 3c and Fig. S7c). In addition, we were able to recapitulate the tethering function of the assembled HOPS complex between GTP-Rab2 decorated liposomes and GTP-Rab39A decorated liposomes (Fig. 4f, g). Moreover, the assembled HOPS between T-Rab2 and V-Rab39A can promote membrane fusion between them (Fig. 4a–e). Compared with yeast, mammalian cells harbor more complexity in regulating HOPS by Rab GTPases, probably because they must cope with more complicated environment. Further study is required to uncover the mechanism how different Rab GTPases recognize the HOPS complex under physiological and pathological conditions.

The gain-of-function mechanisms in C9orf72-related disease were extensively studied recently, includes RNA toxicity from *C9orf72* hexanucleotide repeat expansions^54–57^ and the dipeptide repeat protein aggregates produced by repeat-associated non-ATG translation^54,58–64^. Despite the GOF mechanisms, reduced C9orf72 mRNA or protein levels in series of patient tissue samples and patient-derived cell lines is also a loss of function mechanism which could not be ignored in C9orf72 ALS/FTD pathogenesis^65–67^. To answer the question how C9orf72 protein contributes to ALS/FTD pathogenesis, it would be necessary to precisely dissect what function wild-type C9orf72 protein plays in cellular processes, especially in autophagy, which is critical for protein aggregates degradation. Although the role of C9orf72 in autophagy is extensively discussed in plenty of studies, the conclusions vary case by case. C9orf72-SMCR8-containning complex were reported to act as the effector of Rab1 to affect the ULK1 complex trafficking to phagophore to initiate autophagy, partially explained why there is reduced basal autophagy level and p62 accumulation in ALS/FTD patient-derived iNeurons^48,68^. While in a recent paper, Cleveland et al. found LC3B or p62 level was not changed in C9orf72^-/-^ mice cortical tissues^49^. Reduced autophagy initiation was also found in C9orf72 depleted or SMCR8 knocking down cells^41^, and they claimed that C9orf72-SMCR8-WDR41 complex is the GEF of Rab39B. These studies are somehow consistent with our conclusion, that C9orf72 participates autophagy initiation in a fashion of CSW complex subunits rather than by itself, and the substrate of CSW complex Rab39B is involved in autophagy initiation. Apart from the function of autophagosome formation, C9orf72 was also found to affect the lysosome generation, by regulating mTORC1 activity through Rag GTPases^69^. Moreover, an animal-based research showed that *C9orf72* or *Smcr8* deletion caused impaired lysosomal degradation and exocytosis in macrophages, with defected lysosomal acidifications^70^. While in another study, C9orf72 loss-of-function exhibited down-regulated mTORC1 activity and up-regulated TFEB level, leading to accelerated autophagy level^71^. In summary, at early or late stage of autophagy, C9orf72 all seems to participate, that in the autophagy initiation step, C9orf72 functions as a positive regulator, with SMCR8 and WDR41 as a complex; but in the lysosome formation step, the regulating direction is not clear. Besides, in autophagic fusion step, whether and how C9orf72 participate is still unknown. Based on our analysis in C9orf72 CRISPR/Cas9 knockout cell line and the rescuing experiment, our data strongly support that C9orf72 regulates autophagosome-lysosome fusion, functioning as a GEF for Rab39A by itself, which is required for HOPS complex recruitment onto autophagic vesicle. Further, considering the reported phenomenon that p62-positive inclusion accumulation in C9orf72 ALS/FTD patients’ cerebellum and hippocampus^72–74^, we raise a potential explanation to uncover the autophagy defect mechanism in C9orf72 ALS/FTD patient. That is, by reduced C9orf72 expression and subsequent lack of catalytic activity towards Rab39A, HOPS complex and autophagic SNAREs would be less recruited or assembled as functional complex to mediate normal autophagic fusion. Without effective clearance of aggregates by autophagy, aggravating DPR-associated aggregates accumulation would appear in neuron system and worsen the progression of ALS/FTD disease. Consequently, more neuron system-based C9orf72 GEF activity and autophagy fusion analysis should be conducted to confirm this hypothesis.

Overall, our data not only provided a model of autophagosome-lysosome fusion machinery consisting of tethering factor Rab39A-HOPS-Rab2 complex and membrane fusion executor autophagic SNARE complex in mammal, but also identified C9orf72 as a positive regulator of this fusion machine conferring its function as a GEF for Rab39A. This study therefore illustrates a biochemical function of ALS/FTD-associated protein C9orf72 in Rab39A-HOPS-mediated autophagosome-lysosome fusion.

## Methods

### Antibodies and reagents

Antibodies used in this study included anti-Rab39A (13355, Proteintech), anti-Rab39B (12162, Proteintech), anti-Rab2 (15420, Proteintech), anti-Rab7 (55469, Proteintech), anti-Flag M2 (F3165, Sigma), anti-HA (H3663, Sigma), anti-EGFP (ab6556, Abcam), anti-LC3 (for WB, 7543, Sigma; for IF, PM036 or M152-3, MBL), anti-p62 (H00008878-M01, Abnova), anti-VPS39 (16219, Proteintech), ani-VPS41 (13869, Proteintech), anti-VPS11 (ab189920, Abcam), anti-VPS16 (17776, Proteintech), anti-VPS33A (16896, Proteintech), anti-VPS18 (ab178416, Abcam), anti-C9orf72 (22637, Proteintech), anti-STX17 (HPA001204, Sigma), anti-SNAP29 antibody(sc-135564, Santa Cruz), anti-LAMP2 (sc-18822, Santa Cruz or L0668, Sigma), anti-Myc (2276, Cell Signaling), anti-α -Tubulin (E7, DSHB), anti-VAMP8 (ab76021, Abcam), anti-WIPI2 (Abcam, Ab105459).

Reagents used in this study included LysoTacker (C1046, Beyotime), Torin 1 (F6101, UBP Bio), CQ (C6628, Sigma), Bafilomycin A1 (S1413, Selleck), human EGF (E9644, Sigma), Doxycycline (D9891, Sigma), GST beads (17-0756-05, Cytivia), IgG beads, (17-0969-01, Cytivia), Flag agarose (A2220, Sigma), HA agarose (A2095, Sigma), DiI (D3911, Invitrogen), DiD (D307, Invitrogen), POPE (850757, Avanti), POPC (850457, Avanti), biotin (60338ES10, Yeasen), StrepTactin XT beads (2-5030-025, IBA), Histodenz (D2158, Sigma).

### Cell culture, cell transfection

293T (CRL-1573, ATCC) and U_2_OS (HTB-96, ATCC) cells were cultured in DMEM (D6429, Sigma) supplemented with 10% FBS (10991148, Gibco) and 1% Penicillin-Streptomycin Solution (15-140-122, Gibco). 293S suspension cells were cultured in chemically defined medium (UP1000, Union-biotech). Cell transfection was performed using Lipofectamine 3000 (L3000015, Invitrogen) or PEI (23966-1, Polysciences) according to protocols provided by the manufacturers.

### Oligonucleotides

For the establishment of CRISPR/Cas9 knockout 293T cell lines of Rab39A, sgRNA1 GTTCCGCCTCATCGTGATCGG and sgRNA3 CCTCATCGTGATCGG GGACTC were used. For the establishment of CRISPR/Cas9 knockout 293T cell lines of Rab39B, sgRNA1 CTGATCCGCCGCTTCACCGA and sgRNA2 AGCGACCC TCGGTGAAGCGG were used. For the establishment of CRISPR/Cas9 knockout U_2_OS cell lines of C9orf72, sgRNA AATGGGGATCGCAGCACATA was used. For knockdown of Rab39A, the shRNA oligos CTAGCGACTGTGGAATGAAGTA TATATACTAGTTATATACTTCATTCCACAGTCTTTTTG was cloned into EZ-Tet-PLKO-Hygro vector (Addgene 85972). For the knockdown of C9orf72 in U_2_OS cells by siRNA, the oligos GCACAUAUGGACUAUCAAUTT and GCCACCCU GUCAUGAACAUTT were used.

### Plasmid constructs

STX17, SNAP29, VAMP8 expression plasmids were mentioned in the previous study^33^. The expression plasmids of Rab2, Rab7, Rab39A, Rab39B, C9orf72, VPS11, VPS16, VPS18, VPS33A, VPS39, VPS41 were constructed by cloning these genes from human cDNA (home prepared from 293T cells) into pcDNA5 or pcDNA4 or pLenti-puro or pGEX-4T-1 with tags for eukaryotic or prokaryotic expression.

### Immunofluorescence staining

Cells were grown on coverslips and transfected according to protocols provided by manufacturers with noted plasmids. After 24 h, cells were fixed using ice-cold 4% paraformaldehyde for 10 min. Cells were then washed 3 times with PBS and blocked with blocking buffer (4% BSA + 0.1% Saponin in PBS) at 25 °C for 30 min. Cells were incubated with primary antibodies at 4 °C overnight, washed 3 times with PBS buffer and then incubated with appropriate secondary antibodies for 1 h at 25 °C. Slides were examined under a laser scanning confocal microscope (Olympus IX83).

### Transmission electron microscope analysis

To observe cells under electron microscopy, cells were fixed with 2% glutaraldehyde in 0.1 M sodium cacodylate buffer (pH 7.2) for 2 h followed by 1% osmium tetroxide in 0.1 M sodium cacodylate buffer (pH 7.2) for 2 h. Samples were blocked with 0.5% aqueous uranyl acetate overnight and treated by low-temperature dehydration and infiltration with a graded series of Epon/Araldite, which was followed by the embedment in 100% Epon/Araldite. Thin sections (60 nM) were cut and stained with Reynalds lead citrate and analyzed with a FEI Tecnai 12 Transmission electron microscope. To observe proteoliposomes under electron microscopy, proteoliposomes were decorated with prenylated Rab2 or Rab39A. Next, incubate Rab2-decorated proteoliposomes with two-subunit HOPS subcomplex and Rab39A-decorated proteoliposomes with four-subunit HOPS subcomplex to create the +HOPS group. A control group without HOPS subcomplexes incubation was also prepared. For each group, all the proteoliposomes were mixed together at 4 °C for 30 min and prepared for cryo-EM using a Vitrobot (FEI) with 100% relative humidity. Five microliters of vesicle solutions were incubated on a glow discharged lacey Formavar/carbon 300 mesh copper grid before blotting and plunging into liquid ethane. The frozen-hydrated specimens were subsequently observed at liquid nitrogen temperature in a TF20 electron microscope (FEI) operated at 200 kV, under low dose conditions. Images were collected at a nominal magnification of ×45,000 and a defocus of 2.5–3.5 μm.

### Tandem affinity purification

The Tandem affinity purification (TAP) experiment was performed as described before^75^ with modifications. Briefly, the stable 293T^TetR^ cell line capable of expressing ZZ–3 × Flag-STX17 upon DOX induction was constructed by transfected with ZZ–3 × Flag-STX17, selected by hygromycin and then single clone was picked. The lowest dose of DOX to induce expression of exogenous STX17 close to endogenous level was chosen to apply for TAP. Thus, the cells were grown in DMEM with 10% FBS plus 1% P/S and harvested near confluence. The cell pellet was washed with chilled PBS three times and then suspended in TAP buffer. The resuspended cell pellets were gently vortexed for 1 min after a 30-min incubation on ice. The homogenate was centrifuged for 20 min at 10,000 × *g*. The supernatant was transferred to a fresh tube. Then 0.8 mL of packed IgG beads was added to the supernatant, followed by gentle rotation overnight at 4 °C (12–16 h). The bound protein was eluted by TEV protease cleavage and further purified by anti-FLAG antibody-conjugated beads. The final eluates from the FLAG beads with FLAG peptide were resolved by SDS/PAGE on a 4–12% gradient gel and visualized by silver staining. Specific bands were cut off and subjected to mass spectrometry analysis.

### Recombinant protein purification

All recombinant proteins were expressed and purified according to the detailed protocol previously reported^33,76^ with modifications. For prokaryotic expressed recombinant soluble proteins, including unprenylated Rab GTPases and C9orf72, proteins were expressed individually in BL21 (DE3) at 25 °C. After homogenization in resuspension buffer (20 mM Tris-HCl, pH7.5, 300 mM NaCl, 1 mM TCEP and protease inhibitor) and centrifugation (186,000 × *g*, 1 h at 4 °C), the Glutathione Sepharose 4 Fast Flow beads were used to bind proteins in supernatant. After washing, the proteins were then eluted by cleavage with TEV protease or GSH competitive binding. For prokaryotic expressed recombinant transmembrane proteins, including STX17 and VAMP8 were expressed individually as N-terminal GST-fused proteins from pGEX-4T-1 in BL21 at 25 °C. Cell pellets from 6 L of culture were suspended in buffer (20 mM Tris-HCl, pH7.5, 300 mM NaCl, 1 mM TCEP and protease inhibitor). After homogenization, the inclusion bodies were removed by 30 min spins at 15,340 ×  g in a JA-14 rotor (Beckman Coulter), and the membrane fraction collected by centrifugation at 186,000 *×* *g* for 2 h in a Ti-45 (Beckman Coulter) rotor. Membrane pellets were resuspended in buffer (20 mM HEPES, pH 7.5, 500 mM NaCl, 1 mM TCEP, and 10% glycerol (w/v)). Then dodecylmaltoside was added to 2%, and after solubilization at 4 °C for 1 h, the sample was centrifuged for 1 h at 186,000 *×* *g* in a Ti-45 rotor. Collect the supernatant and binding to Glutathione Sepharose 4 Fast Flow beads. After washing with buffer (20 mM HEPES, pH7.5, 300 mM NaCl, 1 mM TECP, 10% glycerol (w/v), and 1% octyl glucoside), the proteins were then eluted by cleavage with TEV protease. In a separate purification scheme, WT His-tagged SNAP29 was co-expressed with STX17 from pETDuet-1 in BL21 at 25 °C. The extracted membrane fraction in the presence of 2% dodecylmaltoside binds to Ni^2+^-NTA agarose for 2 h and then elute with 350 mM imidazole. For eukaryotic expressed proteins, including prenylated Rab GTPases, HOPS subunits and HOPS subcomplexes, the indicating protein expression plasmids were transfected or co-transfected into 293 S cells and expressed for 2 days. After homogenization in resuspension buffer (20 mM Tris-HCl, pH 7.5, 150 mM NaCl, 1 mM TECP, 0.5% NP-40 and protease inhibitor) and centrifugation (48,380 *×* g, 45 min at 4 °C), the Flag affinity gel (Sigma) or Strep beads (IBA) were used to bind proteins in supernatant. After washing with high salt buffer (20 mM Tris-HCl, pH 7.5, 500 mM NaCl, 1 mM TECP, 0.5% octyl glucoside), the proteins were then eluted by elution buffer containing 2.5 µg/ml Flag peptide or 50 mM biotin. The HOPS subcomplexes were eluted without octyl glucoside.

### Liposome floatation assay

Liposome floatation assay was performed as described previously with some modifications^77^. Liposomes consisting of 2% DiI, 80% POPC and 20% POPE were reconstituted with Rab GTPases if required (lipid:rab = 100:1). Then, the Rab GTPases on proteoliposomes were activated by loading GTP. 165 μl liposomes (500 μM) were gently mixed with 70% (w/v) histodenz in Floatation Buffer (20 mM HEPES pH 7.5, 150 mM NaCl, 2% glycerol) to achieve a final histodenz concentration of 40% (w/v), and placed at the bottom of an Ultra-Clear^TM^ tubes (Beckman Coulter, cat. 344090). The liposomes were then overlaid with 165 μl Floatation Buffer with 35% (w/v) histodenz, 165 μl Floatation Buffer with 30% (w/v) histodenz, and 50 μl buffer (20 mM HEPES pH7.5, 150 mM NaCl). Samples were centrifuged at 280,000 *×* g using an SW55 rotor (Beckman) for 4 h at 4 °C, and the gradient samples (100 µl per fraction) were collected and analyzed with SDS-PAGE and Coomassie blue staining or immunoblotting by noted antibodies. Here the Rab GTPases were purified from 293 S cells.

### In vitro lipid/content mixing assay

The lipid mixing assay was performed as described before^33^ with modifications. Briefly, liposomes consisting of (78% POPC, 20% POPE, 2% DiD or 2% DiI) were incubated with designated SNAREs and prenylated Rab GTPases purified from 293 S cells at desired lipid to membrane-anchored protein (L/P) ratio of approximately 100–200 for 1 h at 4 °C, and dialyzed against 2 L buffer (20 mM HEPES, pH7.5, 150 mM NaCl, 1 mM TCEP, 0.5 mM MgCl_2_) twice at 4 °C for 4 h and then for overnight. For activation (or inactivation) of Rab GTPase, 200 mM GTP (or GDP) was added to proteoliposomes solution in the presence of 2 mM EDTA for 10 min at room temperature. Afterwards, the EDTA was quenched by the addition of 5 mM MgCl_2_.Then different groups of proteoliposomes were mixed with or without HOPS subcomplexes or pre-incubated HOPS complexes. Briefly, donor dyes were excited with 530 nm laser light and emission fluorescence intensity was monitored by fluorescence spectrophotometer (Cary Eclipse fluorescence spectrophotometer, Agilent Technologies) at 670 nm. Lipid mixing was measured as the fluorescence emission (670 nm) of DiD acceptor dyes arising from FRET upon excitation of DiI dyes with 530 nm light. For content-mixing assay, self-quenched sulphorhodamine B (50 mM) molecules encapsulated in v-SNARE proteoliposomes were used as content indicator. Content mixing was measured by an increase of fluorescence emission at 570 nm of the sulphorhodamine B dyes upon excitation with 530 nm laser light that results as the initially self-quenched dye is diluted upon complete fusion between labeled v-SNARE and unlabeled t-SNARE proteoliposomes. Each assay was repeated for at least 3 times.

### Immunoprecipitation assay

Cells were lysed using tandem affinity purification buffer as described above. Whole-cell lysates (input) were collected after pelleting cellular debris using centrifugation. Lysate was then incubated with a 20:1 ratio of lysate to anti-FlagM2 Affinity Gel (Sigma) for 16 h at 4 °C. Beads were washed three times with tandem affinity purification buffer and then eluted with a 1:10 ratio of 200 µg/ml Flag peptides. Samples were subjected to SDS–PAGE. Immunoblotting was performed following standard procedures.

### In vitro GEF activity assay

The Mant-GDP release assay was performed as described before^78^ with modifications. Briefly, the purified Rab GTPases were preloaded by MANT-GDP (M12414, Invitrogen) at 25 °C. The nucleotide exchange reaction was recorded using a fluorescence spectrophotometer (Victor Nivo, PerkinElmer) at an excitation wavelength of 366 nm and an emission wavelength of 443 nm for noted time. The proteins used in this experiment were purified from *E. coli*, except for C9orf72/SMCR8 complex, which is a gift from Shiqian Qi (Sichuan University).

### Quantification of imaging data

For quantification of colocalization, separate channels of images were defined by threshold, and then Pearson’s correlation coefficient was quantified by “Measuring colocalization” plugin in Olympus Cellsense software with region of interest (ROI). To quantify puncta number, images were thresholded in Fiji, and then the positive puncta were counted using the “analyze particles” menu from Fiji. To quantify the mean area of LAMP2 puncta, images were thresholded in Fiji, and then the area was analyzed using the “Measure-Area” menu from Fiji.

### Statistics and reproducibility

In general, data shown in column graphs represent the mean ± SD or mean ± SEM, or plot all as box and whiskers if the sample size is more than 10, indicated in the figure legends. The box always extends from the 25th to 75th percentiles, and the whiskers go down to the smallest value and up to the largest. Experiments in Figs. 1a, b, d, f, g, h; 2a, d, e, g, i, 3a-h; 4a; 5a, b, d, g; 6a, b, c, e, g, i; 7a, d, f, h, i are independently repeated three times. No data were excluded. Statistical analysis used GraphPad Prism8 or Excel.

### Reporting summary

Further information on research design is available in the Nature Portfolio Reporting Summary linked to this article.

### Supplementary information

Supplementary Information

Description of additional supplementary files

Supplementary Data 1

Reporting Summary

### Source data

Source Data

## Data Availability

The Mass Spectrometry data generated in this study have been deposited in the PRIDE server under accession code PXD043048. Source data are provided with this paper.
