# Supplementary material for: C9orf72-catalyzed GTP loading of Rab39A enables HOPS-mediated membrane tethering and fusion in mammalian autophagy
Source: Nat Commun. 2023 Oct 11;14:6360. doi: 10.1038/s41467-023-42003-0 (PMC10567733; doi:10.1038/s41467-023-42003-0)

Supplementary Figures

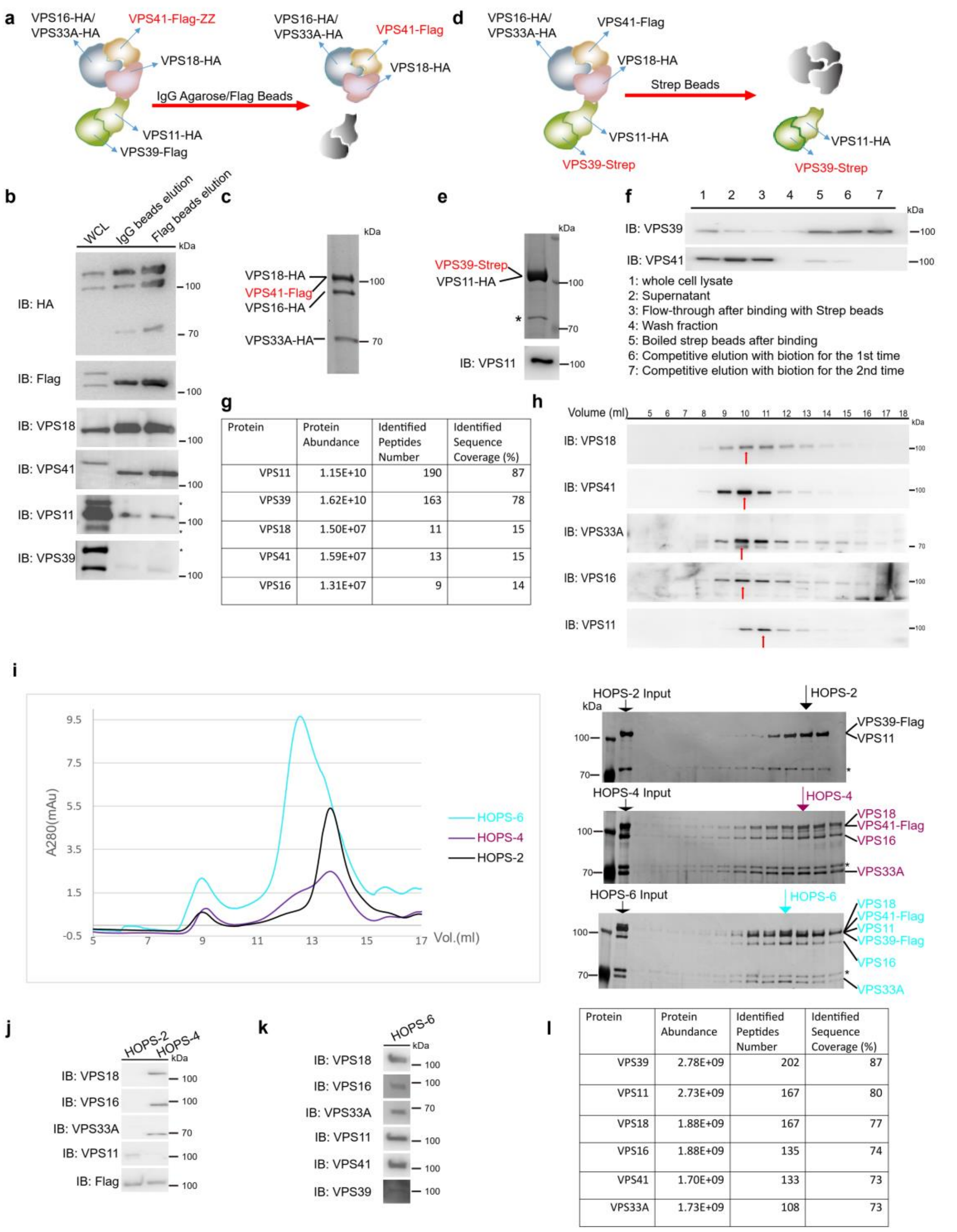

Figure S1. Mammalian HOPS complex purification and assembly by "hooking up" two HOPS subcomplexes.

(a) Schematic drawing of tandem affinity-purified incomplete HOPS complex. Red-colored VPS41-Flag-ZZ was used as bait. **(b)**

Immunoblotting results of tandem affinity-purified incomplete HOPS complex from 293s cells transfected with HOPS subunits expression plasmids shown in **(a)** by indicated antibodies. Asterisk indicates nonspecific band. WCL, whole cell lysate. **(c)** Coomassie blue staining gel of tandem affinity-purified incomplete HOPS complex from 293s cells transfected with HOPS subunits plasmids as in **(a)**. **(d)** Schematic drawing of Strep affinity-purified incomplete HOPS complex. Red-colored VPS39-Strep was used as bait. **(e)** Gel analysis of Strep affinity-purified incomplete HOPS complex from 293s cells transfected with HOPS complex subunits expression plasmids as shown in **(d)** by Coomassie blue staining or immunoblotting with VPS11 antibody. Asterisk in Coomassie blue staining indicates impurities. **(f)** Immunoblotting results of Strep affinity-purified incomplete HOPS complex by indicated antibodies purified as described in **(d)**. **(g)** Mass Spectrometry results of Strep affinity-purified incomplete HOPS complex as described in **(d)**. **(h)** 293T cell lysate was applied to Superdex ™ 200 Increase 10/300 GL column (Cytiva) and the fractions were analyzed by immunoblotting with indicated antibodies. Red arrows indicate the most protein-enriched lanes among the same SDS-PAGE gel. **(i)** Assembly of human HOPS complex *in vitro*. HOPS-2 subcomplex: VPS39-Flag/ VPS11; HOPS-4 subcomplex: VPS41-Flag/ VPS16/ VPS18/ VPS33A. 293S cells were co-transfected with indicated expression plasmids, then collected and lysed for Flag-tag

affinity purification of HOPS-2 or HOPS-4 subcomplex. Left panel showed stacked UV profiles analyzed by SEC (Superose ™6 Increase 10/300 GL, Cytiva). Right panels showed the SDS-PAGE gel of protein fractions, stained by silver staining. The arrows indicated the most protein-enriched lanes in relevant subcomplexes or assembled complex of HOPS. **(j)** Immunoblotting results of Flag affinity purified HOPS-2 subcomplex and HOPS-4 subcomplex as described in **(i)**. **(k)** Immunoblotting results of assembled six-subunit HOPS complex peak fraction indicated in **(i)**. **(l)** Mass Spectrometry results of six-subunit HOPS complex peak fraction (Fig. **S1i**, cyan peak). Source data are provided.

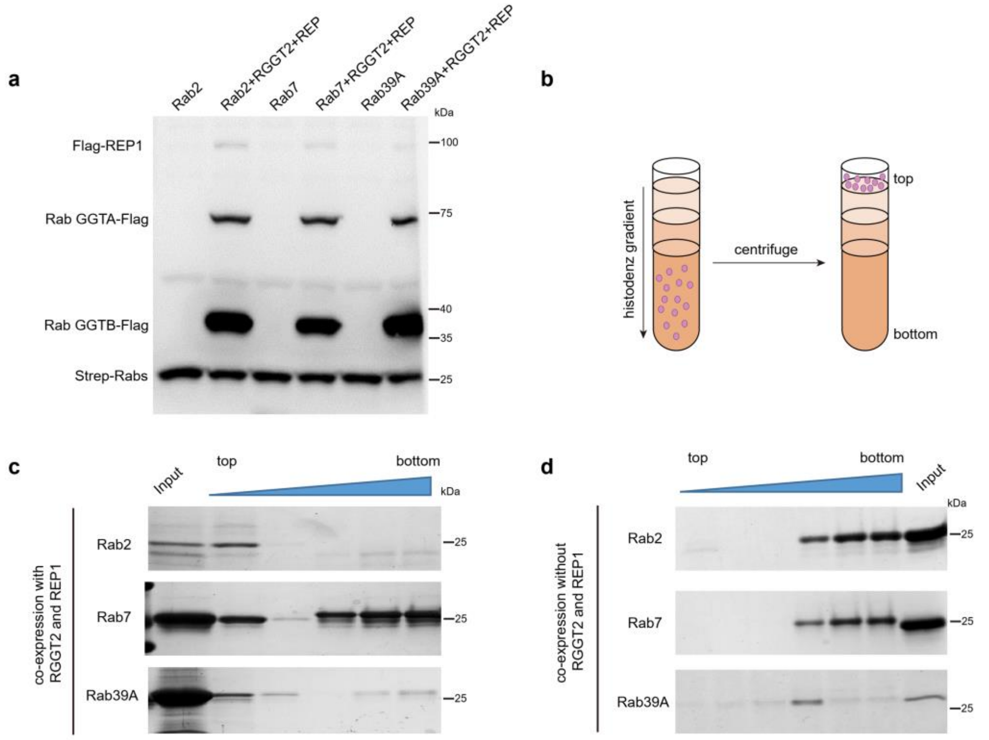

Figure S2. Preparation of prenylated Rab GTPases from mammalian expression system.

(a) Immunoblotting result stained by antibodies anti Flag and Strep of whole cell lysate expressing prenylated Rab GTPases and the chaperon proteins. 293S cells were transfected with indicated Strep tagged Rab GTPases expression plasmid or combined with Flag tagged Rab geranylgeranyl transferase (RGGT2, contains Rab GGTA, 73kD, and Rab GGTB, 37kD) and Flag tagged Rab escort protein 1 (REP1, 95kD). (**b**) Schematic diagram outlining the steps involved in the liposomes co-floatation assay. The liposomes reconstituted with Rab GTPases were isolated by a co-floatation assay on a Histodenz density gradient (40%:35%:30%). After centrifugation, the floated fractions were collected

and analyzed by SDS-PAGE and Coomassie blue staining. **(c)** Analysis of membrane anchoring of prenylated Rab GTPases (Purified Rab GTPases co-expression with RGGT2 and REP1 in 293S cells). The floated fractions were analyzed by SDS-PAGE and Coomassie blue staining. **(d)** Analysis of membrane anchoring of unprenylated Rab GTPases (Purified Rab GTPases without co-expression RGGT2 nor REP1 in 293S cells). The floated fractions were analyzed by SDS-PAGE and Coomassie blue staining. Source data are provided.

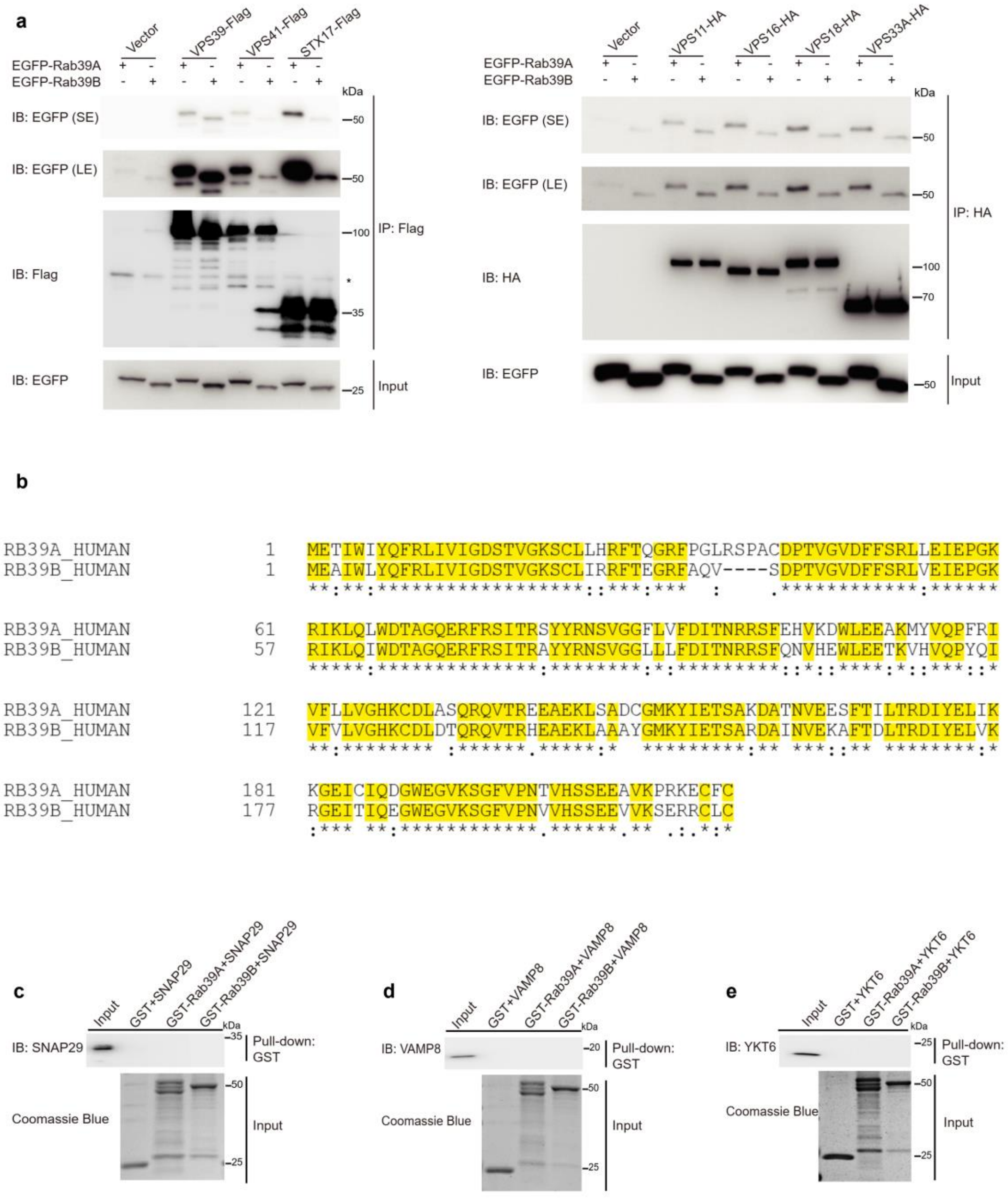

Figure S3. Rab39A and Rab39B, sharing high identity of amino acid sequence, interacted with both STX17 and HOPS complex proteins, but none of SNAP29, VAMP8 and YKT6.

(a) Rab39A/B interacts with STX17 or HOPS complex subunits. 293T cells were transfected with empty vector or tagged protein expression

plasmid as indicated. 24 hours after transfection, cells were lysed and immunoprecipitated with Flag M2 agarose (Left panel) or HA agarose (Right panel)**.** Immunoblotting was performed with indicated antibodies. SE: short exposure, LE: long exposure *,Heavy chain of antibodies. **(b)** Alignment of amino acid sequences between human Rab39A (R39A) and human Rab39B (R39B) using Uniport website tools. Yellow-colored characters show same amino acid. *, indicates positions which have a single, fully conserved residue. (period), indicates conservation between groups of weakly similar properties-scoring=< 0.5 in the Gonnet PAM 250 matrix (colon), indicates conservation between groups of strongly similar properties-scoring>0.5 in the Gonnet PAM 250 matrix. **(c), (d), (e)** Neither Rab39A nor Rab39B binds to autophagic SNAREs SNAP29, VAMP8 and YKT6. Glutathione beads was blocked with BSA or conjugated with GST or GST-Rab39A or GST-Rab39B, then Glutathione beads was incubated with purified SNAP29 **(c)**, VAMP8 **(d)** or YKT6 **(e)** respectively, and then washed and boiled for immunoblotting by indicated antibodies or Coomassie blue staining. Source data are provided.

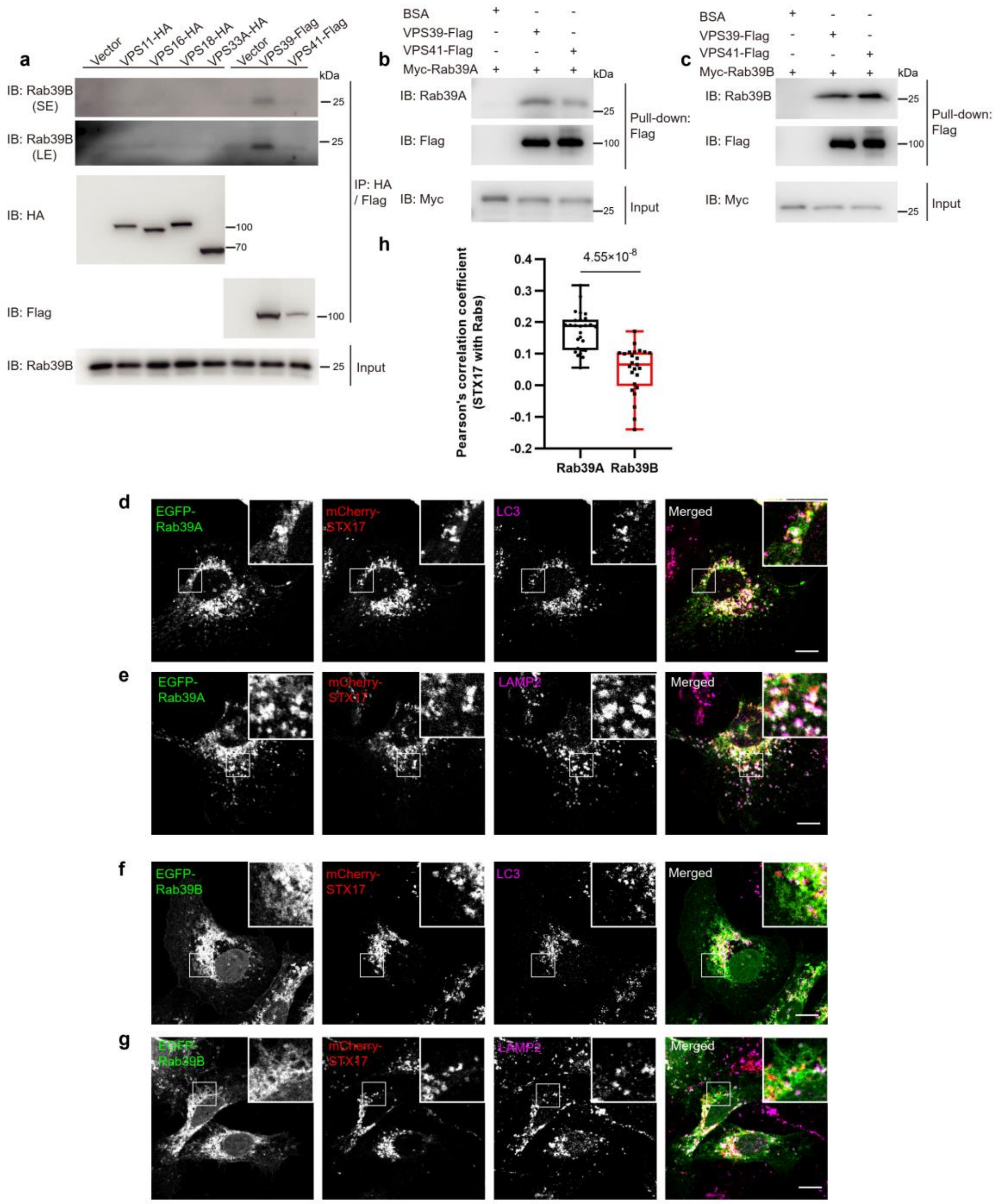

Figure S4. Rab39A and Rab39B displayed interaction with HOPS complex subunits and colocalization with distinct autophagic membrane structures.

(a) Endogenous Rab39B mainly interacts with HOPS complex subunit VPS39. 293T cells were transfected with empty vector or tagged protein

expression plasmid as indicated. 24 hours after transfection, cells were lysed and immunoprecipitated with Flag M2 agarose or HA agarose. Immunoblotting was performed with indicated antibodies. SE, short exposure; LE, long exposure. **(b), (c)** VPS39-Flag or VPS41-Flag directly interacts with Myc-Rab39A and Myc-Rab39B. Flag M2 agarose was blocked with BSA or conjugated with purified VPS39-Flag or VPS41-Flag from 293S cells, then incubated with purified Myc-Rab39A **(b)** or Myc-Rab39B **(c)** respectively, and then washed and eluted for immunoblotting. **(d)** Rab39A co-localized with STX17 and LAMP2. U_2OS cells stably expressing STX17-mCherry were transfected with EGFP-Rab39A, then treated with 50 nM Torin 1 for 3 h. Cells were then fixed and immunostained with anti-LAMP2 antibody. White-framed squares show the zoomed area. Scale bar, 10 μm. **(e)** Rab39A co-localized with STX17 and LC3. U_2OS cells stably expressing STX17-mCherry were transfected with EGFP-Rab39A, then treated with 50 nM Torin 1 for 3 h. Cells were then fixed and immunostained with anti-LC3 antibody. White-framed squares show the zoomed area. Scale bar, 10 μm. **(f)** Rab39B partially co-localized with STX17 and LAMP2. U_2OS cells stably expressing STX17-mCherry were transfected with EGFP-Rab39B, then treated with 50 nM Torin 1 for 3 h. Cells were then fixed and immunostained with anti-LAMP2 antibody. White-framed squares show the zoomed area. Scale bar, 10 μm. **(g)** Rab39B partially co-localized with STX17 and LC3. U_2OS cells stably

expressing STX17- mCherry were transfected with EGFP-Rab39B, then treated with 50 nM Torin 1 for 3 h. Cells were then fixed and immunostained with anti-LC3 antibody. White-framed squares show the zoomed area. Scale bar, 10 μm. **(h)** Statistic results of colocalization analysis between STX17 and Rab39A or Rab39B showed in **(d)-(g)**. Each point represents one image for analyzing. n=27, 25, which contain 101, 103 cells respectively. The box extends from the 25th to 75th percentiles, and the whiskers go down to the smallest value and up to the largest. Significance is determined by two-tailed T test. P value is listed. Source data are provided.

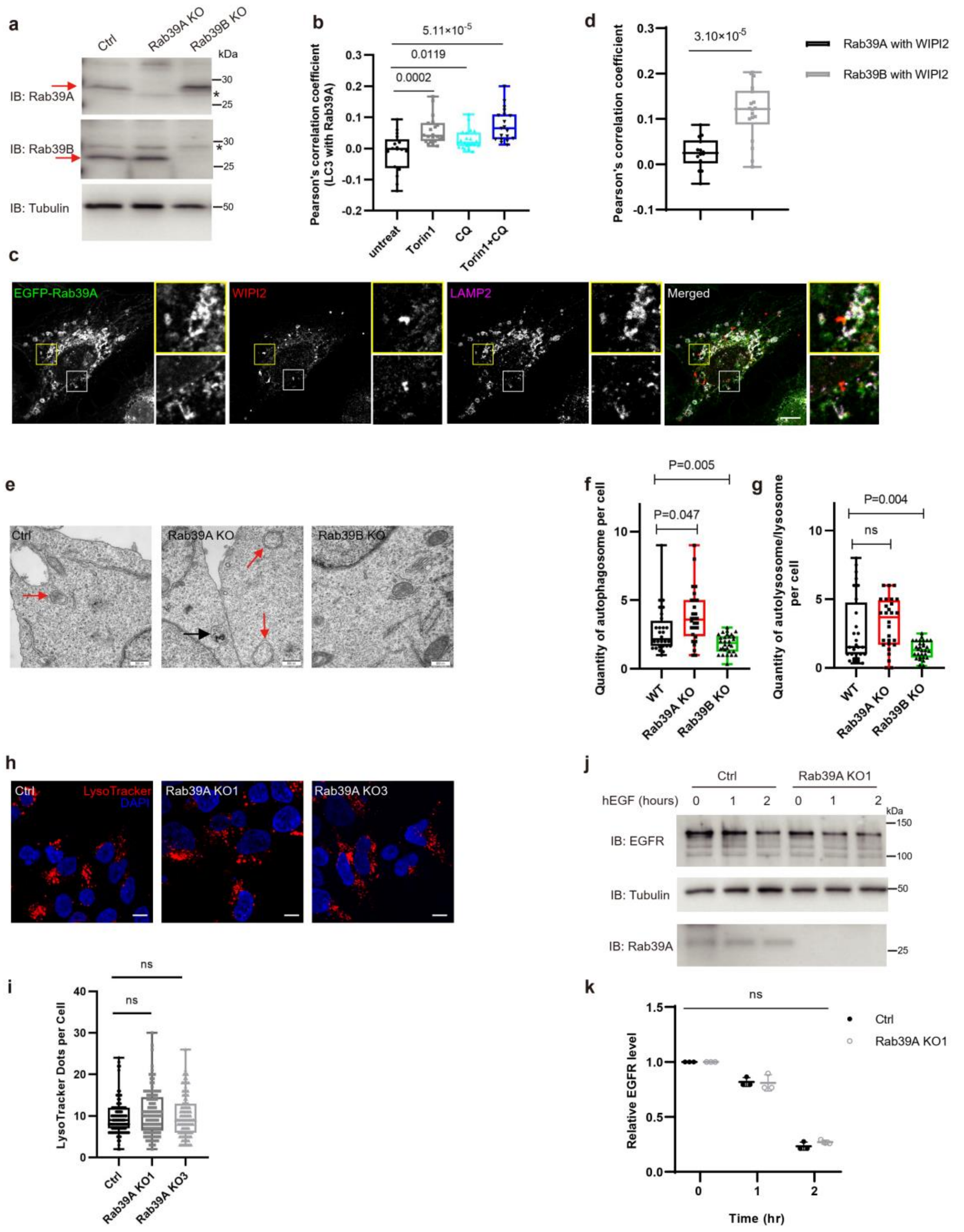

Figure S5. Different effects of Rab39A and Rab39B on distinct stages of autophagy.

(a) Knockout of Rab39A or Rab39B did not alter the commutative expression. Cells were collected and lysed for immunoblotting by indicated antibodies. Asterisk indicates nonspecific band. Red arrows indicate the

position of indicated bands. **(b)** Co-localization of Rab39A and LC3 was analyzed from results of **Figure 2d**. Each point represents an image for analyzing, n=17, 22, 22, 19, which contain 127, 164, 119, 129 cells. **(c)** U_2OS cells with inducible expression of EGFP-Rab39A were treated with 50 nM Torin 1 for 3 h. Then cells were fixed and immunostained with antibodies against WIPI2 and LAMP2. White-framed squares show two representative enlarged areas. Scale bar, 10 μm. **(d)** Quantification result of **(c)** and **Figure S6E**. Each point represents one image for analyzing, n=14, 18, which contain 174, 159 cells. **(e)** Transmission electron microscopy was used after cells are treated with 50 nM Torin 1 for 3 h. Black arrows indicate autolysosomes/lysosomes, and red arrows indicate autophagosomes. Scale bar, 500 nm. **(f), (g)** Quantification of the results of **(e)** for 28 (WT, containing 73 cells; and Rab39A KO, containing 63 cells) or 30 (Rab39B KO, containing 104 cells) random areas per group, average number of double-membraned autophagosome **(f)** or autolysosome/lysosome **(g)** per cell were shown respectively. **(h)** LysoTracker dots were analyzed after cells were treated with 50 nM Torin 1 for 3 h and incubated with LysoTracker for 30 min under fluorescence microscopy. Scale bar, 10 μm. **(i)** Quantification of LysoTracker dots per cell in **(h)**. Data are mean ±SD for 105 cells per group. **(j)** EGFR degradation level was analyzed after cells were treated with human EGF (200 ng/ml) for indicated time. **(k)** Quantification of **(j)** from three

independent repeats. EGFR Level was divided by Tubulin and then normalized with setting it to 1.0 at 0-hour time point. All Data are mean ± SD. Significance was determined by two-tailed T test. ns, not significant. The box extends from the 25th to 75th percentiles, and the whiskers go down to the smallest value and up to the largest. Source data are provided.

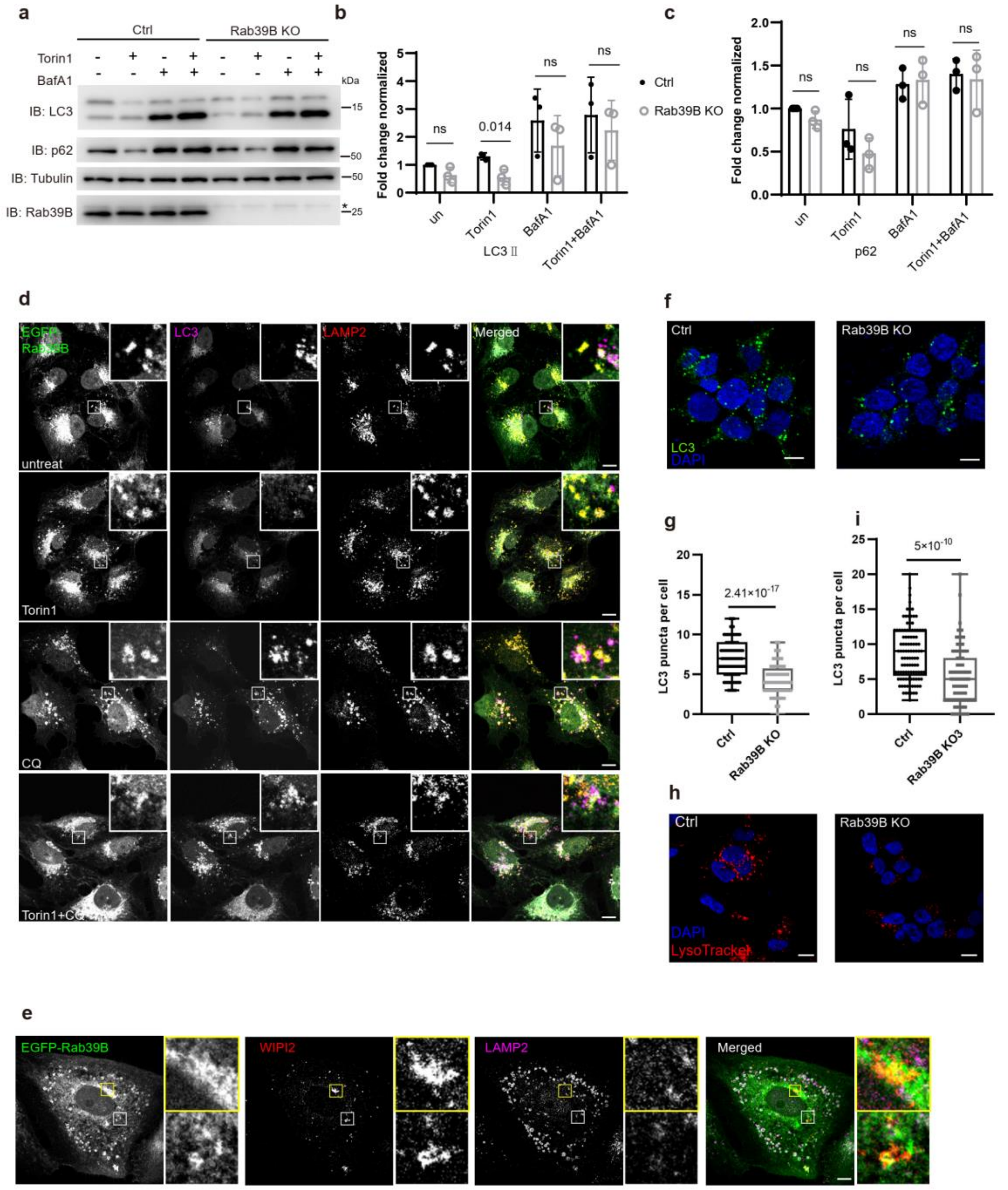

Figure S6. Rab39B has mild effect on autophagy flux.

(a) Autophagy flux was analyzed after cells were treated with 50 nM Torin1 with or without 100 nM Bafilomycin A1 for 3 h and then immunoblotted. **(b) (c)** Statistic results of **(a)** indicating LC3II **(b)** and p62 **(c)** levels from three independent experiments. LC3II and p62 value of

each sample is first divided by the corresponding Tubulin value, then normalized to lane 1 sample. **(d)** EGFP-Rab39B partially residents on autolysosomes. U_2OS cells with inducible expression of EGFP-Rab39B were treated with 50 nM Torin 1 with or without 50 μM CQ for 3 h, then fixed and immune stained with antibodies against LAMP2 and LC3. Images were collected by confocal microscopy. White-framed squares show zoomed area. **(e)** EGFP-Rab39B distributes on phagophores upon autophagy induction. U_2OS cells with inducible expression of EGFP-Rab39B were treated with 50 nM Torin 1 for 3 h. Then cells were fixed and immune stained with antibodies against WIPI2 and LAMP2. Images were collected by confocal microscopy. White-framed squares show two representative enlarged areas. **(f)** LC3-positive autophagic vesicles were decreased in Rab39B KO cells compared with Ctrl cells. Cells were treated with 50 nM Torin 1 for 3 h, then fixed and immune stained with LC3 antibody. DAPI staining indicated nuclei. Images were obtained by fluorescence microscopy. **(g)** Quantification result of **(f)**. Data are mean ± SD for 108 cells per group. **(h)** LysoTracker dots was analyzed after cells were treated with 50 nM Torin 1 for 3 h and incubated with LysoTracker for 30 min under fluorescence microscopy. **(i)** Quantification of LysoTracker dots per cell in **(h)**. Data are mean ± SD for 105 cells per group. All scale bar, 10 μm. All data are mean ± SD. Significance was determined by two-tailed T test. ns, not significant. The box extends from

the 25th to 75th percentiles, and the whiskers go down to the smallest value and up to the largest. Source data are provided.

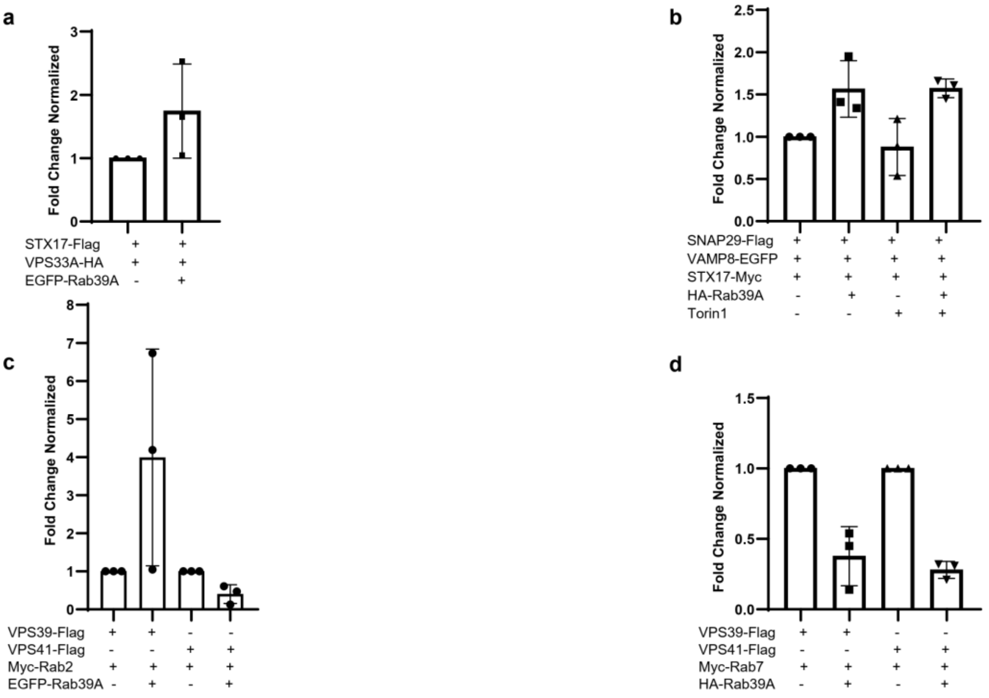

Figure S7. Rab39A enhances assembly of autophagic fusion machinery. **(a)** Quantification results of three independent repeats of **Figure 3a**. Fold change normalized represents the ratio of immunoprecipitated VPS33A-HA band intensity to STX17-Flag band intensity, which were normalized to the lane 3 sample. **(b)** Quantification results of three independent repeats of **Figure 3b**. Fold change normalized represents the ratio of immunoprecipitated VAMP8-EGFP or STX17-Myc band intensity to SNAP29-Flag band intensity, which were normalized to the lane 3 sample. **(c)** Quantification results of three independent repeats of **Figure 3c**. Fold change normalized represents the ratio of immunoprecipitated Myc-Rab2 band intensity to VPS39-Flag or VPS41-Flag band intensity. Lane 5 sample was normalized to the lane 3 sample, and lane 8 sample was normalized to the lane 6 sample. **(d)** Quantification results of three independent repeats

of **Figure 3c**. Fold change normalized represents the ratio of immunoprecipitated Myc-Rab7 band intensity to VPS39-Flag or VPS41-Flag band intensity. Lane 5 sample was normalized to the lane 4 sample, and lane 8 sample was normalized to the lane 7 sample. Data are mean± SD. Source data are provided.

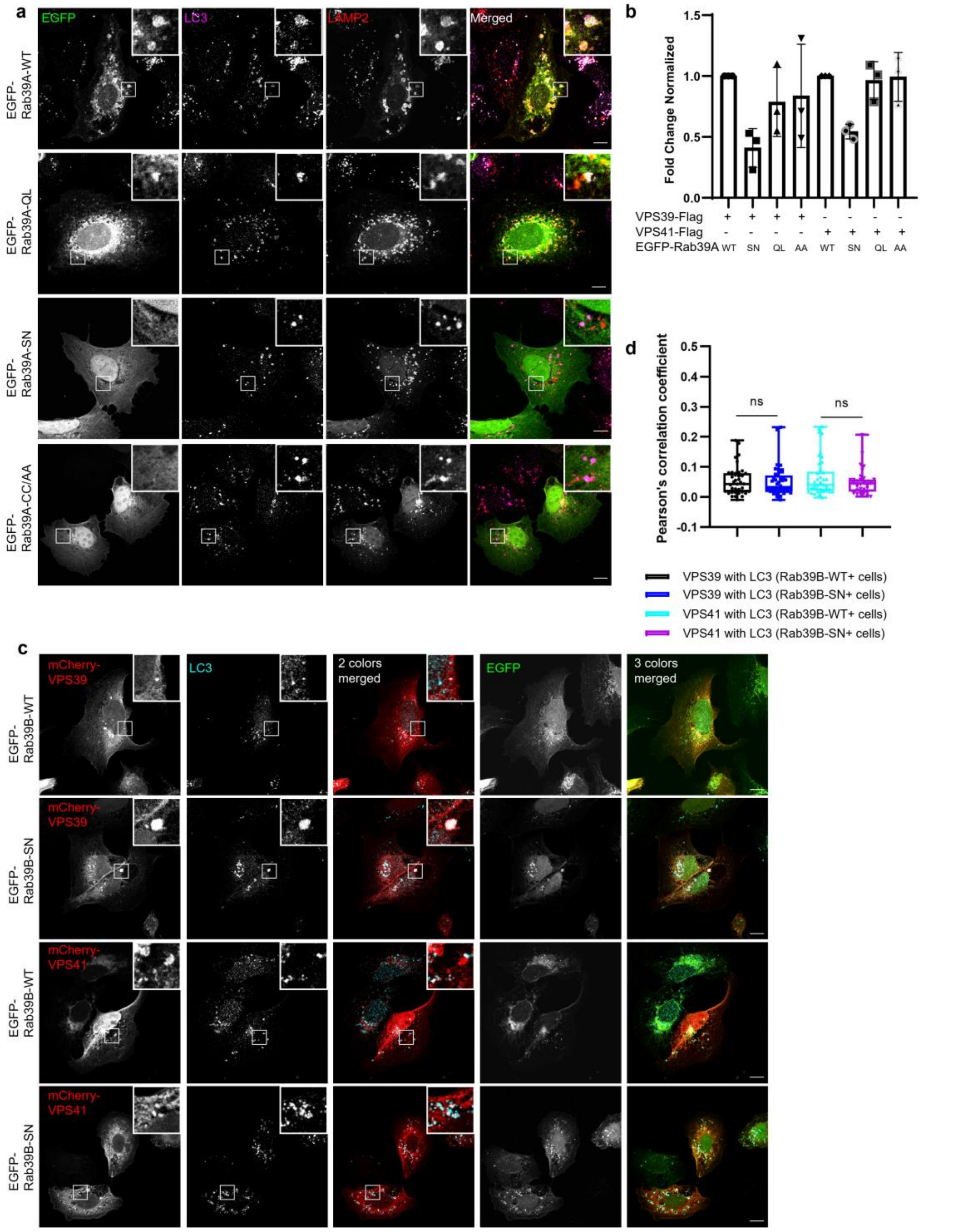

Figure S8. GTP-loading or C-terminal prenylation affects distribution of Rab39A, and inactivation of Rab39B did not affect HOPS complex subunits recruitment onto autophagic structures.

(a) Distribution of Rab39A on autophagic vesicles depends on its GTP-loading activation and C-terminal prenylation. U_2OS cells were transfected

with EGFP-Vector, EGFP-Rab39A-WT, EGFP-Rab39A-Q72L, EGFP-Rab39A-S22N, EGFP-Rab39A-CCAA respectively. After treated with 50 nM Torin 1 for 3 h, cells were fixed and immune stained with indicated antibodies. Scale bar, 10 μm. White-framed squares show zoomed area. (b) Quantification results of three independent repeats of **Figure 5a**. Fold change normalized represents the ratio of immunoprecipitated EGFP-Rab39A WT or SN or QL or AA band intensity to VPS39-Flag or VPS41-Flag band intensity. Lane 6,7,8 samples were normalized to the lane 5 sample, and lane 10, 11, 12 samples were normalized to the lane 9 sample. Data are mean ± SD. **(c)** Inactivation of Rab39B does not affect HOPS complex subunits recruitment onto autophagic vesicles. U_2OS cells were transfected with mCherry-VPS39 or mCherry-VPS41, and co-transfection of Rab39B-WT or Rab39B-S22N. After treated with 50 nM Torin 1 and 50 μM CQ for 3 h, cells were fixed and immune stained with indicated antibody. White-framed squares show zoomed area. Scale bar, 10 μm. **(d)** Quantification results of colocalization analysis between HOPS complex subunits and LC3 in Rab39B-WT+ cells or Rab39B-SN+ cells. Each point represents one image for analyzing, n=41, 42, 42, 34, which contains 103, 104, 106, 102 cells. Significance was determined by two-tailed T test. ns, not significant. The box extends from the 25th to 75th percentiles, and the whiskers go down to the smallest value and up to the largest. Source data are provided.

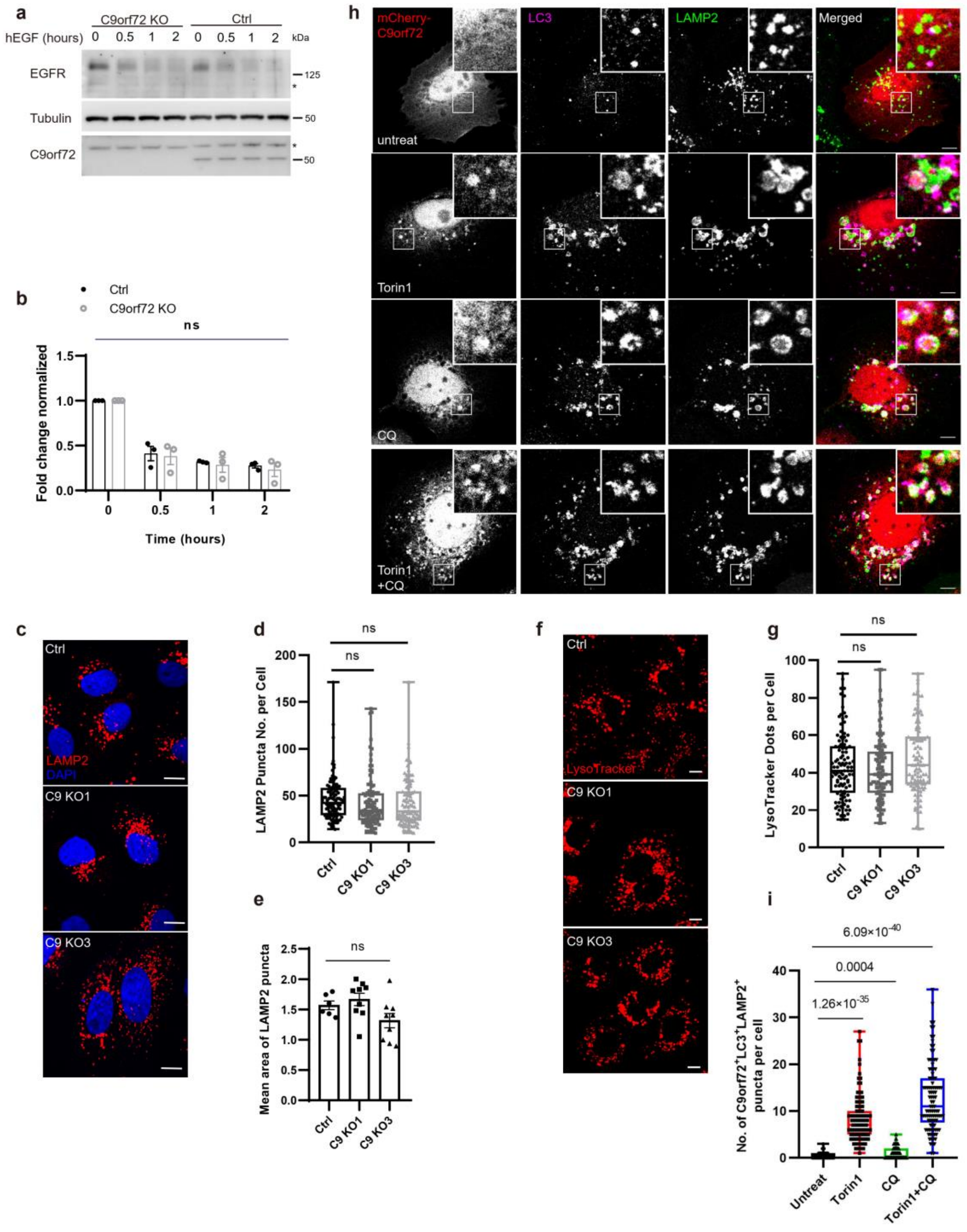

Figure S9. C9orf72 did not affect lysosome activity.

(a) EGFR degradation level was analyzed by immunoblotting after cells were added with human EGF (200 ng/ml) for the indicated time. The band intensity was measured in three independent experiments, and statistic data was shown in **(b)**. EGFR levels were divided by Tubulin levels and then

normalized by setting it to 1.0 at 0-hour timepoint. **(c)** Morphology and quantity of lysosome is not altered in C9orf72 KO cells. Cells were fixed and immunostained with LAMP2 antibody. DAPI staining indicated nuclei. Images were collected by confocal microscopy. **(d)** Quantity of lysosomes per cell were analyzed from **(c)**. Data are mean ± SD for 105 cells per group. **(e)** Quantification of lysosome size were analyzed from results of **(c).** Each point represents one image for analyzing, n=6,9,9, which contain 159, 202, 199 cells. **(f)** Formation of lysosomes is not affected in C9orf72 KO cells. Cells were treated with 50 nM Torin 1 for 3 h and incubated with LysoTracker for 30 min, then observed by fluorescence microscopy. **(g)** Quantification results in **(f)** amount of LysoTracker dots per cell. Data are mean ± SD for 105 cells per group. **(h)** Distribution of C9orf72 in U_2OS cells. U_2OS cells were transfected with mCherry-C9orf72 for 24 h. After seeding and treatment of 50 nM Torin 1 with or without 50 μM CQ for 3 h, cells were fixed and immunostained with antibodies against LC3 and LAMP2. Images were collected by confocal microscopy. White-framed squares show zoomed areas. (**i**) Quantification results from **(h)**. Each point represents one cell, n=101, 103, 102, 101. KO, knockout; C9, C9orf72. All data are mean ± SD. All scale bar is 10μm. Significance was determined by two-tailed T test. P value is listed. The box extends from the 25th to 75th percentiles, and the whiskers go down to the smallest value and up to the largest. Source data are provided.

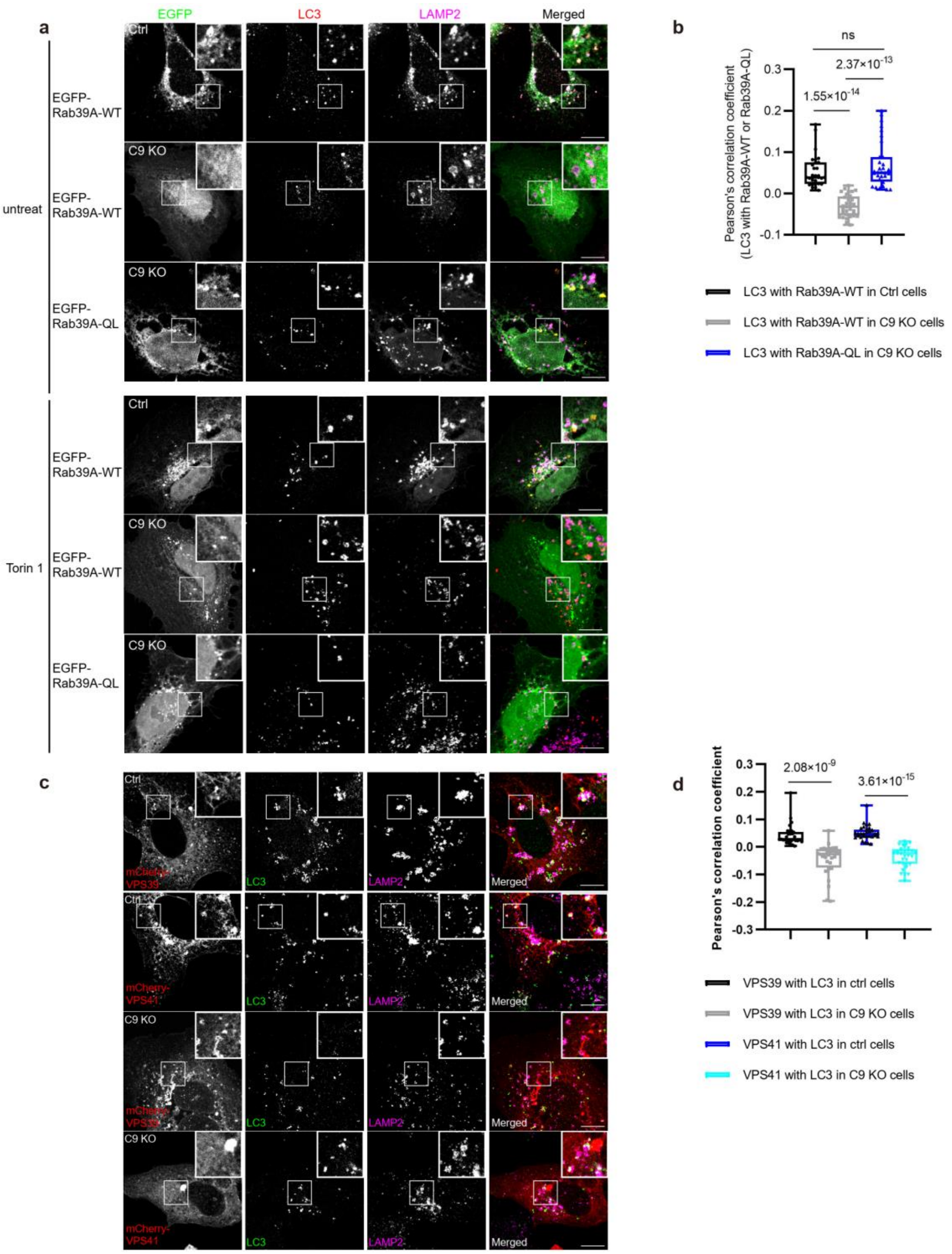

Figure S10. C9orf72 enables Rab39A's recruitment of the HOPS complex onto autophagic structures.

(a) Depletion of C9orf72 inhibits distribution of Rab39A on autophagic structures. Control or C9orf72 KO U_2OS cells were transfected with EGFP-Rab39A-WT or EGFP-Rab39A-QL. After untreated or treated with

50 nM Torin 1 for 3 h, cells were fixed and immune stained with indicated antibody. White-framed squares show zoomed area. Scale bar, 10 μm **(b)** Quantification results of Torin 1 treated cells in **(a)** combining with **Figure. 2d**. each point represents one image for analyzing, n=29, 33, 32, which contain 127, 103, 106 cells. Significance was determined by two-tailed T test. P value is listed. ns, not significant. **(c)** Depletion of C9orf72 inhibits distribution of HOPS complex subunits on autophagic structures. C9orf72 KO U_2OS cells were transfected with mCherry-VPS39 or mCherry-VPS41. After treated with 50 nM Torin 1 for 3 h, cells were fixed and immune stained with indicated antibody. Scale bar, 10 μm. **(d)** Quantification results of **(c)**. each point represents one image for analyzing, n=30, 31, 31, 32, which contain 101, 106, 104, 102 cells. Significance was determined by two-tailed T test. P value is listed. The box extends from the 25th to 75th percentiles, and the whiskers go down to the smallest value and up to the largest. Source data are provided.

Figure S1

b

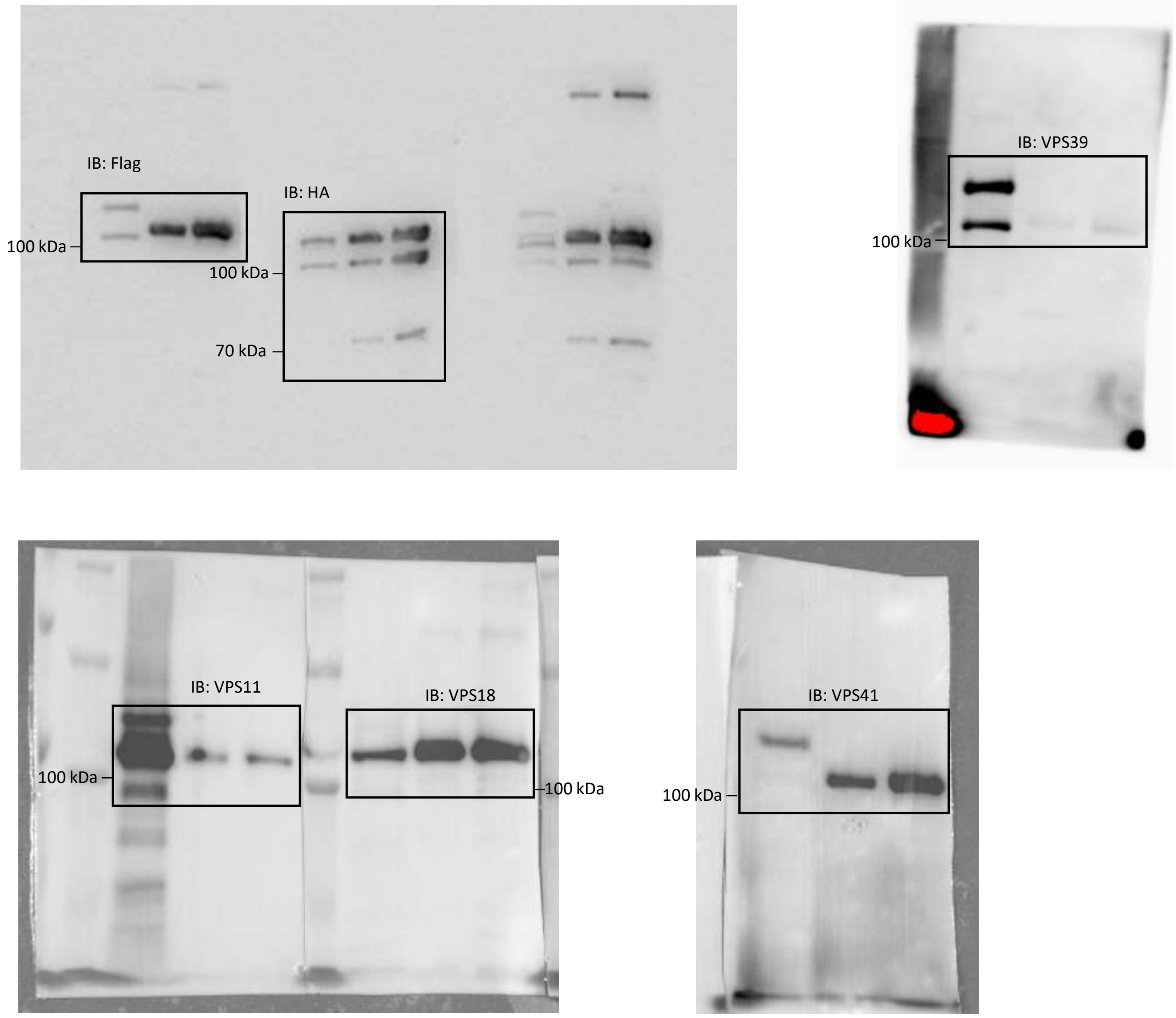

c

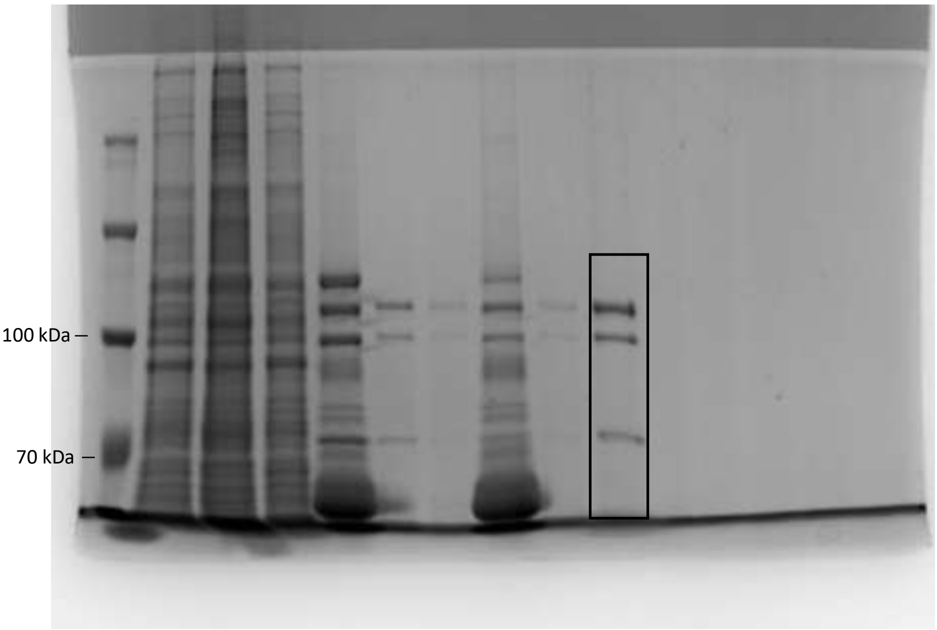

e

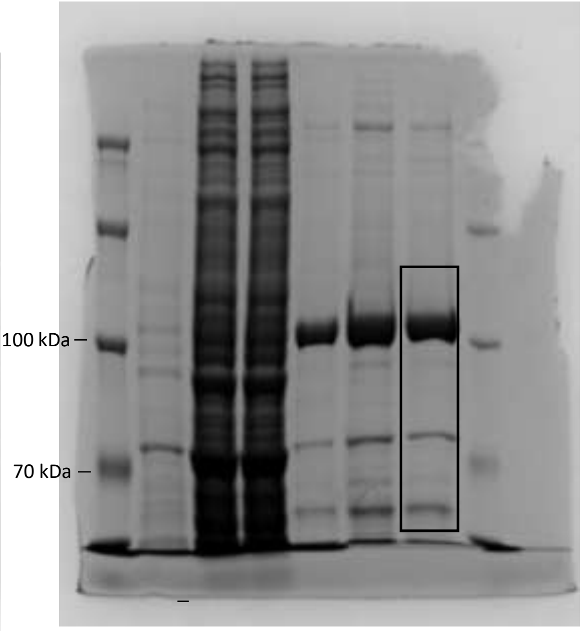

Figure S1

f

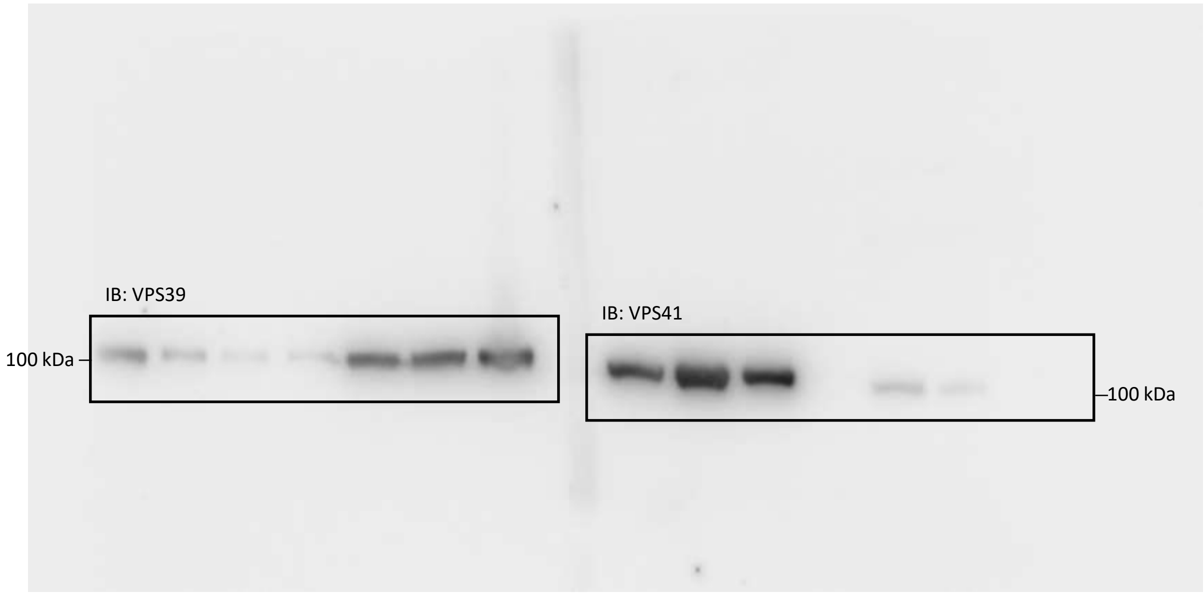
IB: VPS39
100 kDa
IB: VPS41
100 kDa

h

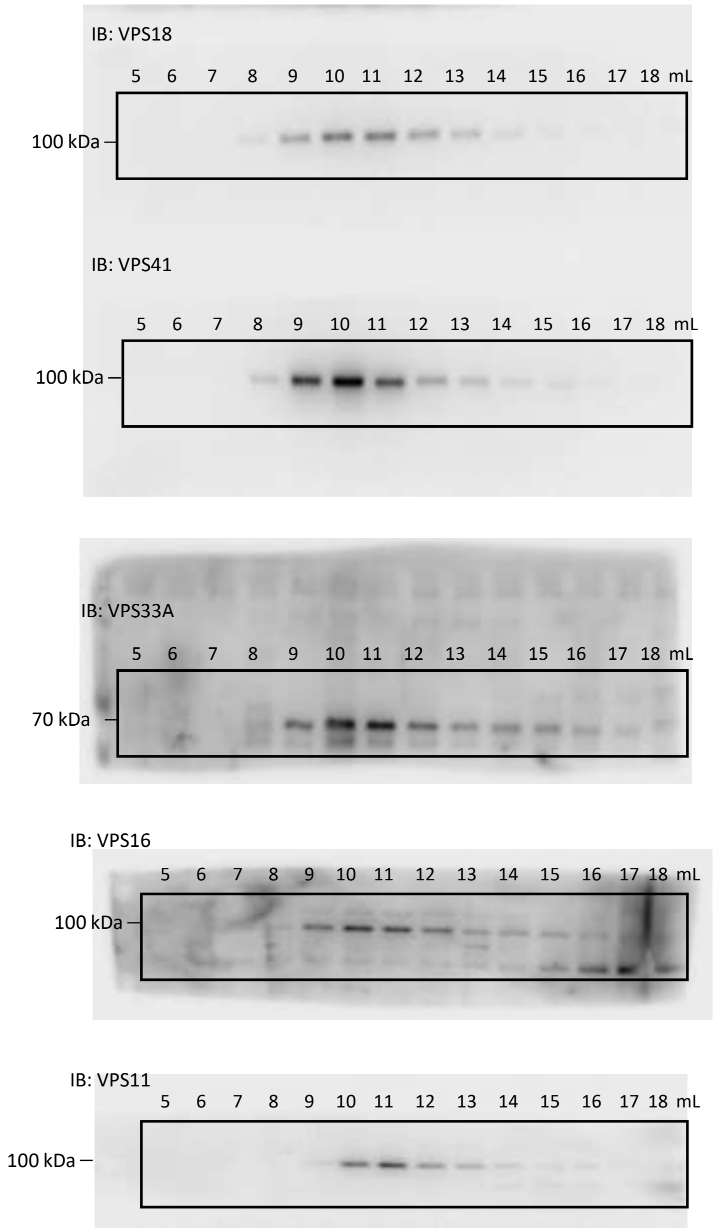
IB: VPS18
5 6 7 8 9 10 11 12 13 14 15 16 17 18 mL
100 kDa
IB: VPS41
5 6 7 8 9 10 11 12 13 14 15 16 17 18 mL
100 kDa
IB: VPS33A
5 6 7 8 9 10 11 12 13 14 15 16 17 18 mL
70 kDa
IB: VPS16
5 6 7 8 9 10 11 12 13 14 15 16 17 18 mL
100 kDa
IB: VPS11
5 6 7 8 9 10 11 12 13 14 15 16 17 18 mL
100 kDa

Figure S1

i

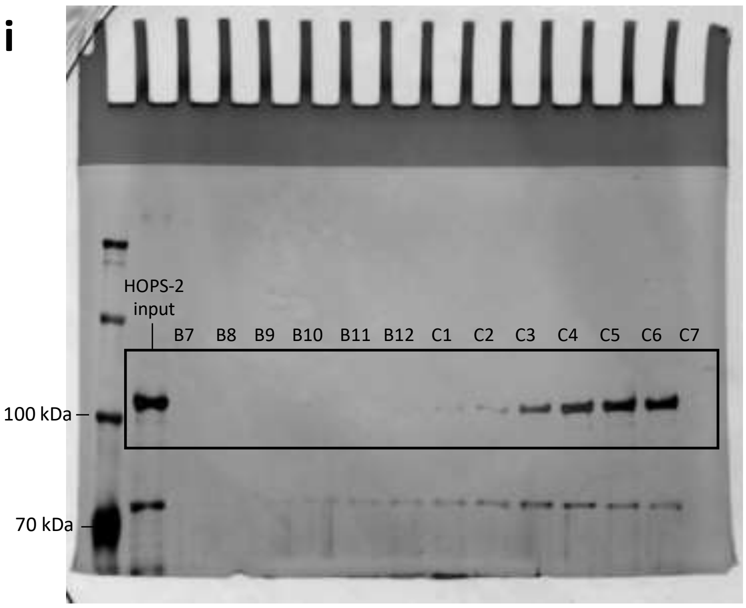

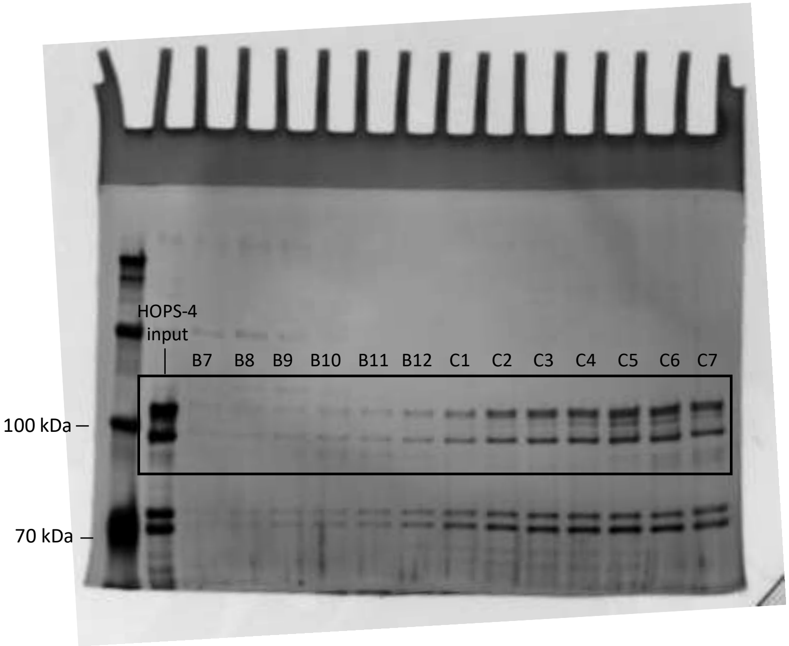

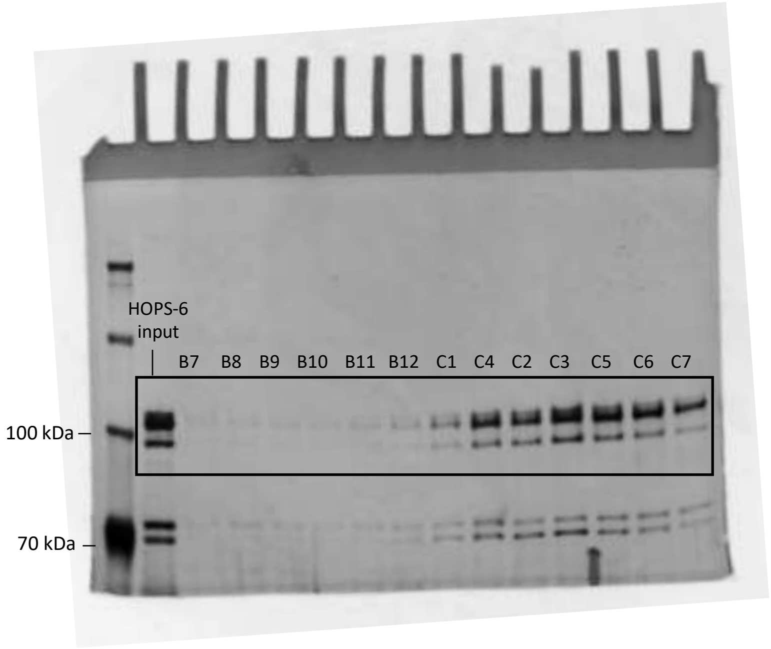

J

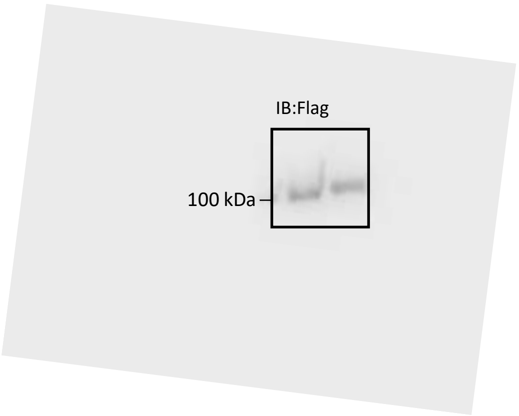

j

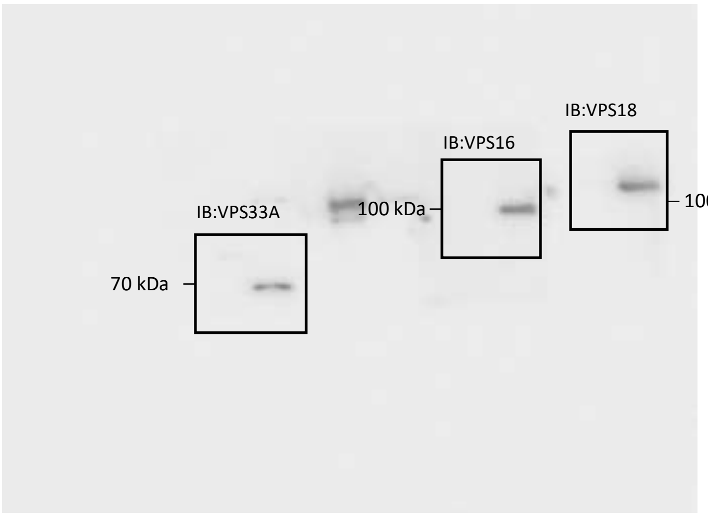

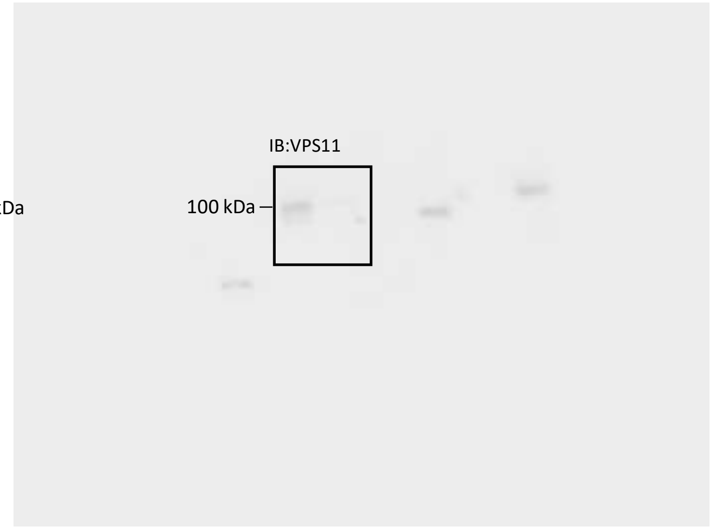

Figure S1

k

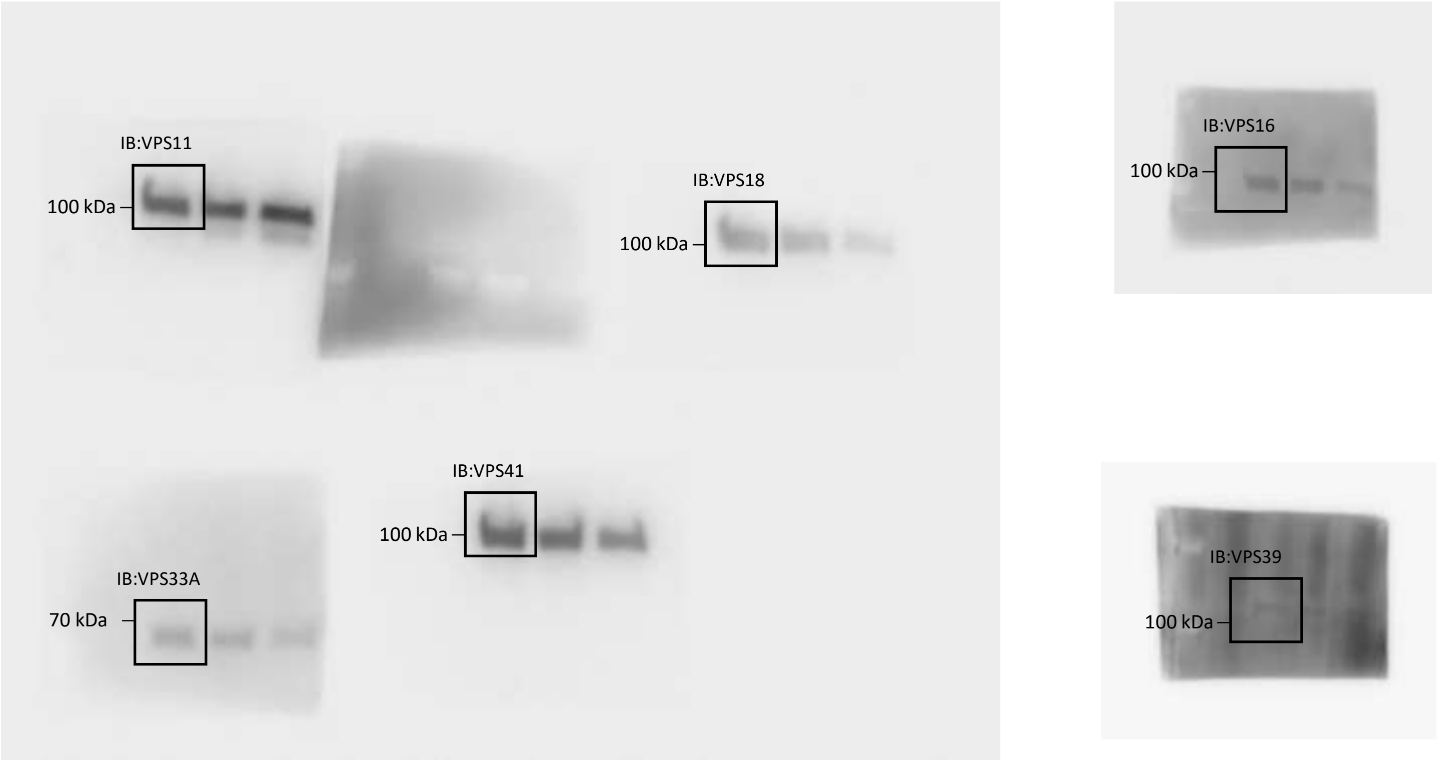

IB:VPS11
100 kDa
IB:VPS18
100 kDa
IB:VPS33A
70 kDa
IB:VPS41
100 kDa
IB:VPS16
100 kDa
IB:VPS39
100 kDa

Figure S2

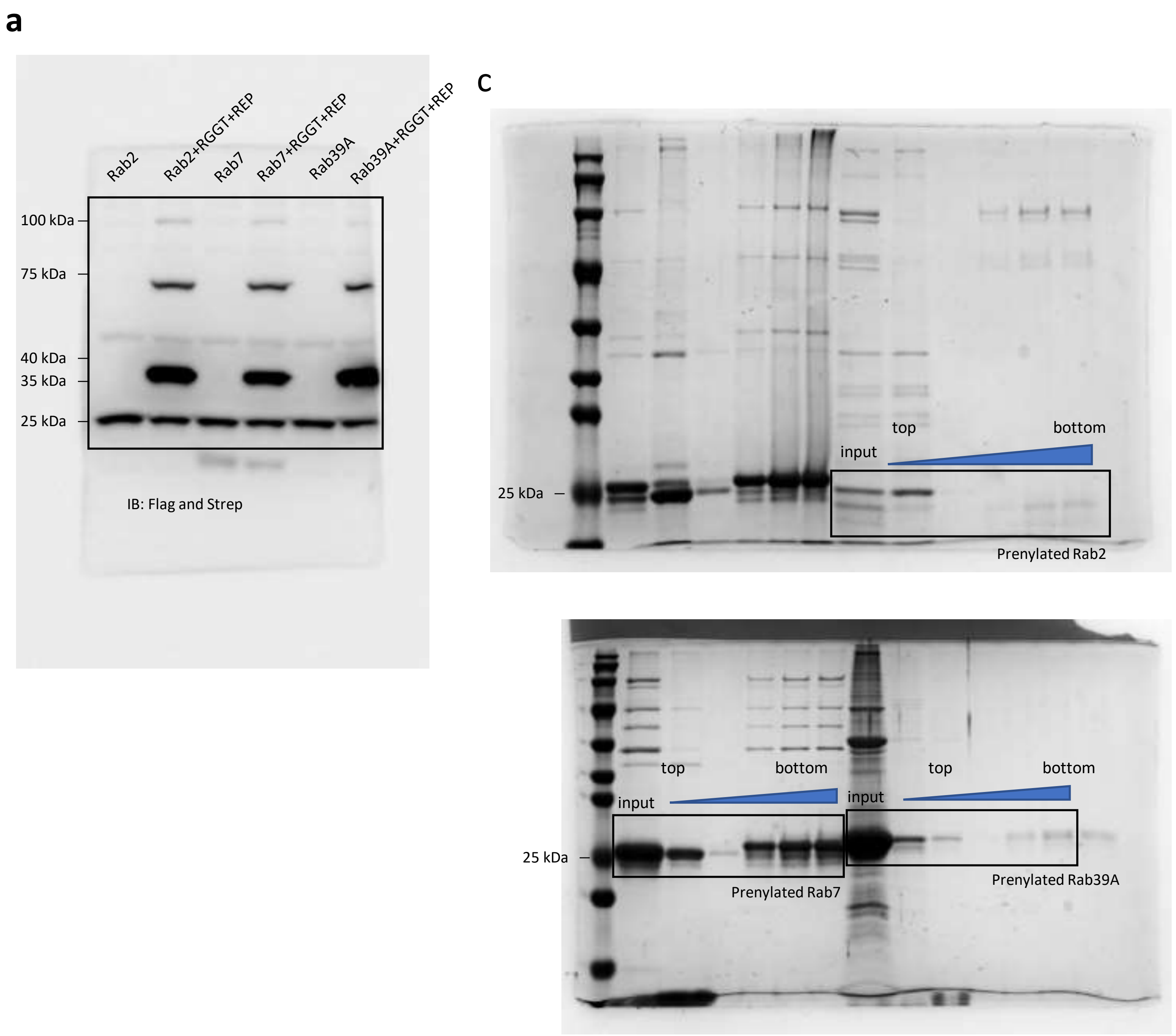
a
Rab2
Rab2+RGGT+REP
Rab7
Rab7+RGGT+REP
Rab39A
Rab39A+RGGT+REP
100 kDa
75 kDa
40 kDa
35 kDa
25 kDa
IB: Flag and Strep
C
25 kDa
input
top
bottom
Prenylated Rab2
input
top
bottom
Prenylated Rab7
input
top
bottom
Prenylated Rab39A

Figure S2

d

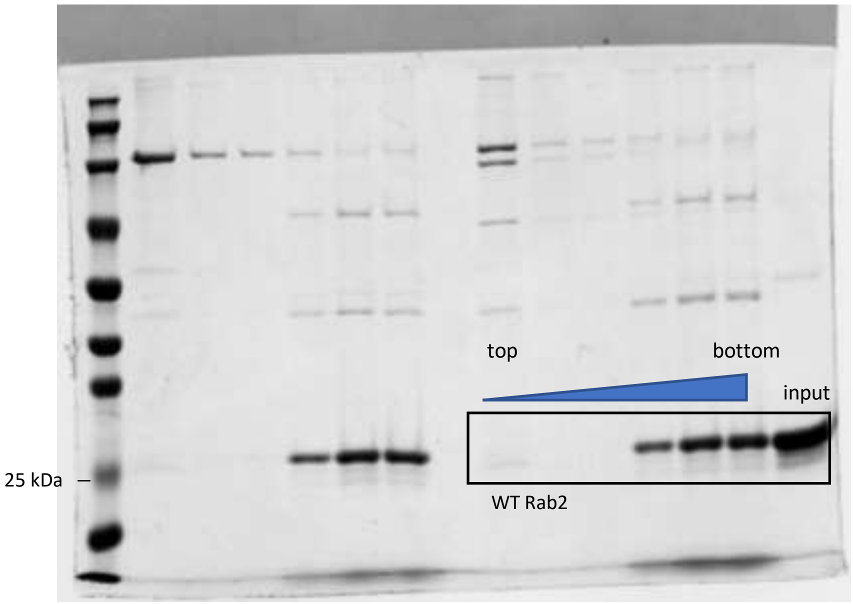

top
bottom
input
25 kDa
WT Rab2

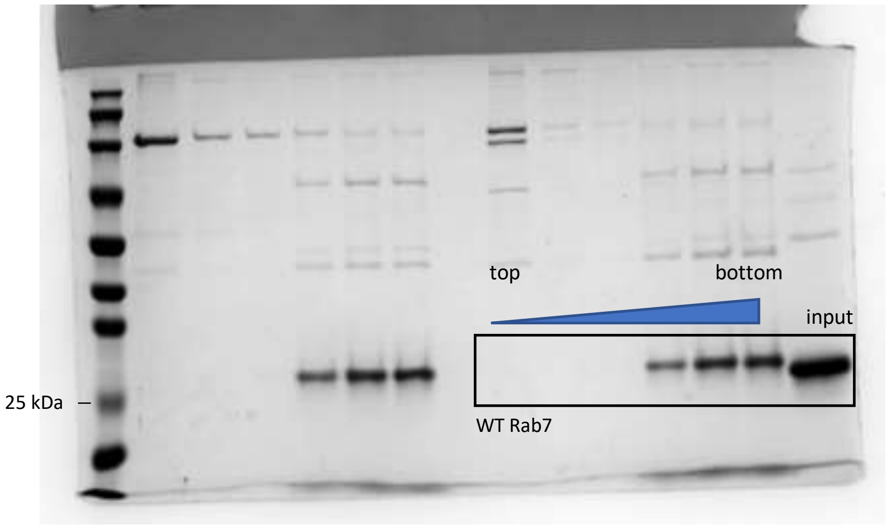

top
bottom
input
25 kDa
WT Rab7

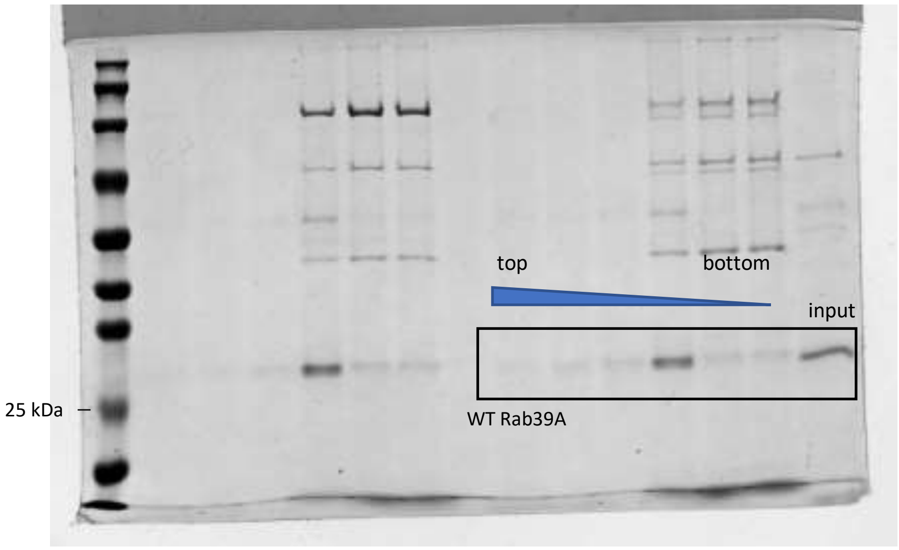

top
bottom
input
25 kDa
WT Rab39A

Figure S3

a

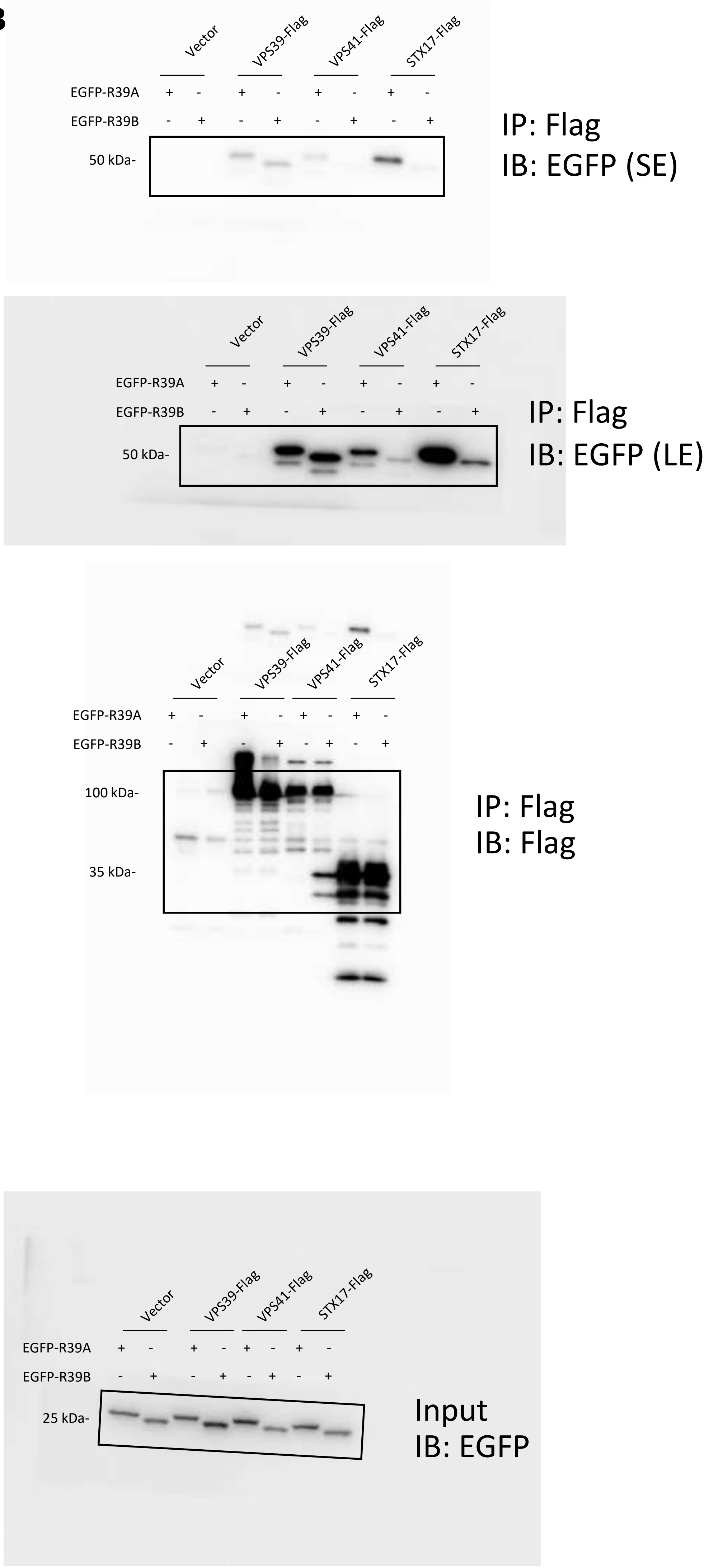
Vector
VPS39-Flag
VPS41-Flag
STX17-Flag
EGFP-R39A
EGFP-R39B
50 kDa-
IP: Flag
IB: EGFP (SE)
IP: Flag
IB: EGFP (LE)
100 kDa-
35 kDa-
IP: Flag
IB: Flag
25 kDa-
Input
IB: EGFP

Figure S3

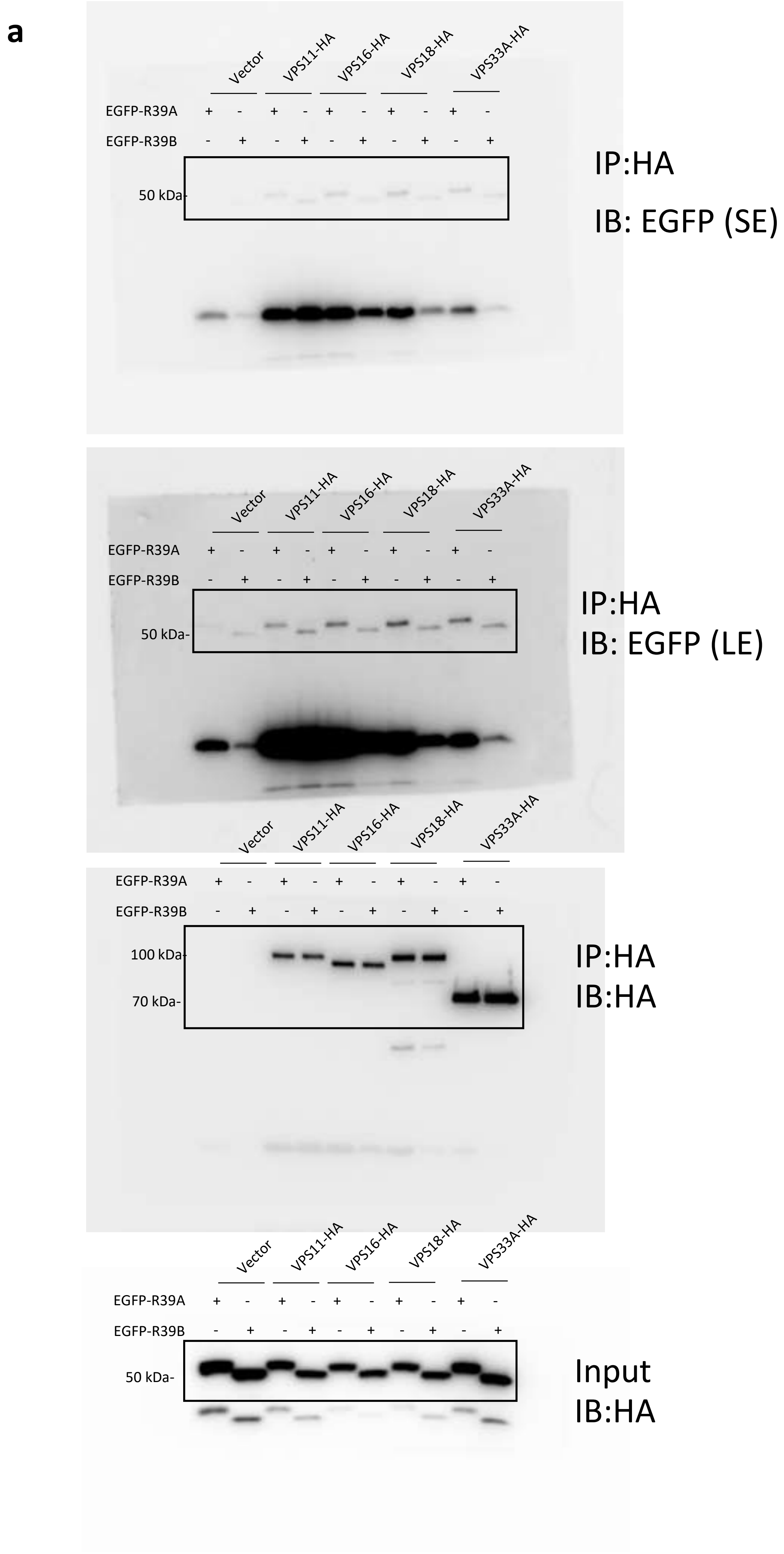
a
Vector
VPS11-HA
VPS16-HA
VPS18-HA
VPS33A-HA
EGFP-R39A + - + - + - + - + -
EGFP-R39B - + - + - + - + - +
50 kDa
IP:HA
IB: EGFP (SE)
Vector
VPS11-HA
VPS16-HA
VPS18-HA
VPS33A-HA
EGFP-R39A + - + - + - + - + -
EGFP-R39B - + - + - + - + - +
50 kDa-
IP:HA
IB: EGFP (LE)
Vector
VPS11-HA
VPS16-HA
VPS18-HA
VPS33A-HA
EGFP-R39A + - + - + - + - + -
EGFP-R39B - + - + - + - + - +
100 kDa
70 kDa-
IP:HA
IB:HA
Vector
VPS11-HA
VPS16-HA
VPS18-HA
VPS33A-HA
EGFP-R39A + - + - + - + - + -
EGFP-R39B - + - + - + - + - +
50 kDa-
Input
IB:HA

Figure S3

c,d,e

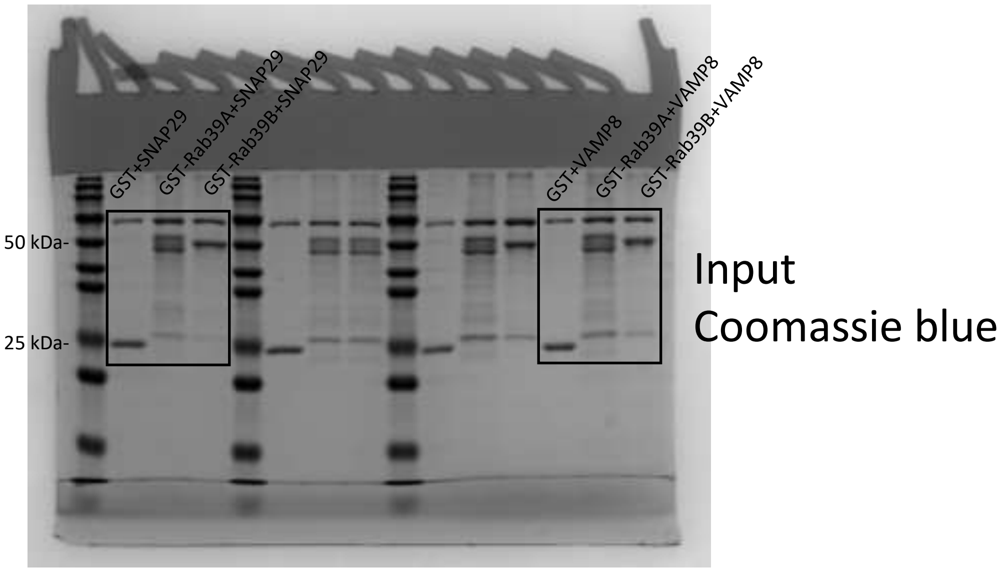

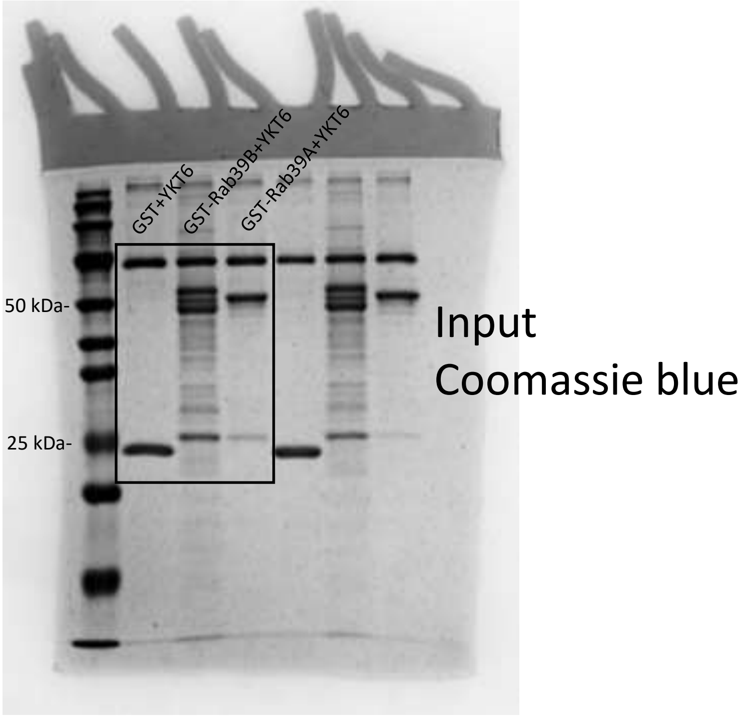

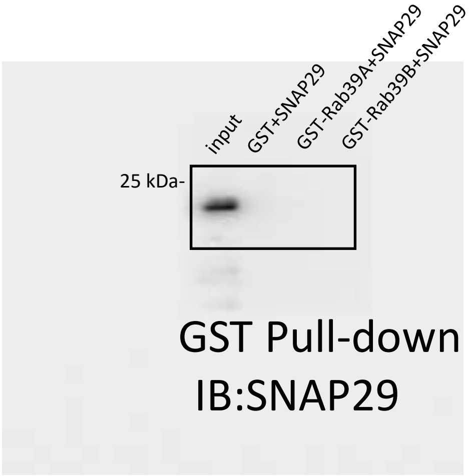

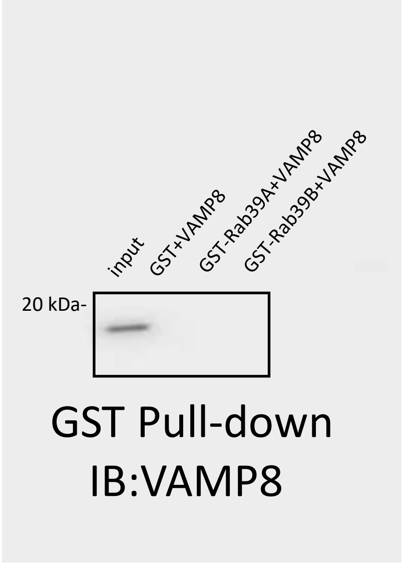

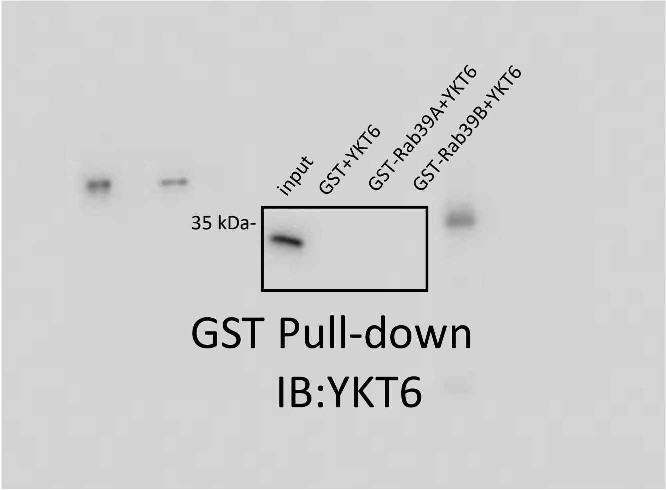

Figure S4

a

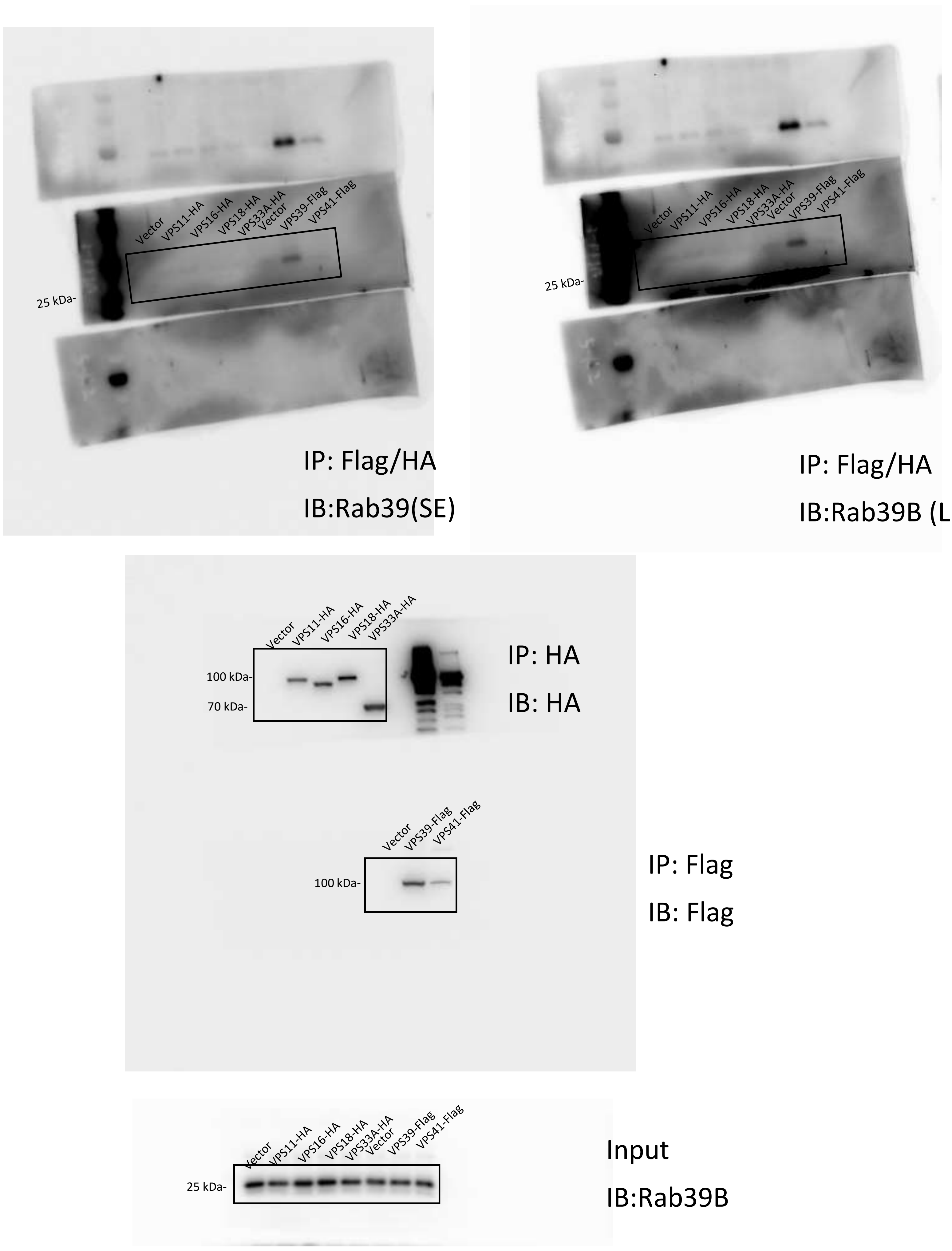
Vector
VPS11-HA
VPS16-HA
VPS18-HA
VPS33A-HA
Vector
VPS39-Flag
VPS41-Flag
25 kDa-
IP: Flag/HA
IB:Rab39(SE)
Vector
VPS11-HA
VPS16-HA
VPS18-HA
VPS33A-HA
Vector
VPS39-Flag
VPS41-Flag
25 kDa-
IP: Flag/HA
IB:Rab39B (LE
Vector
VPS11-HA
VPS16-HA
VPS18-HA
VPS33A-HA
100 kDa-
70 kDa-
IP: HA
IB: HA
Vector
VPS39-Flag
VPS41-Flag
100 kDa-
IP: Flag
IB: Flag
Vector
VPS11-HA
VPS16-HA
VPS18-HA
VPS33A-HA
Vector
VPS39-Flag
VPS41-Flag
25 kDa-
Input
IB:Rab39B

Figure S4

b and c

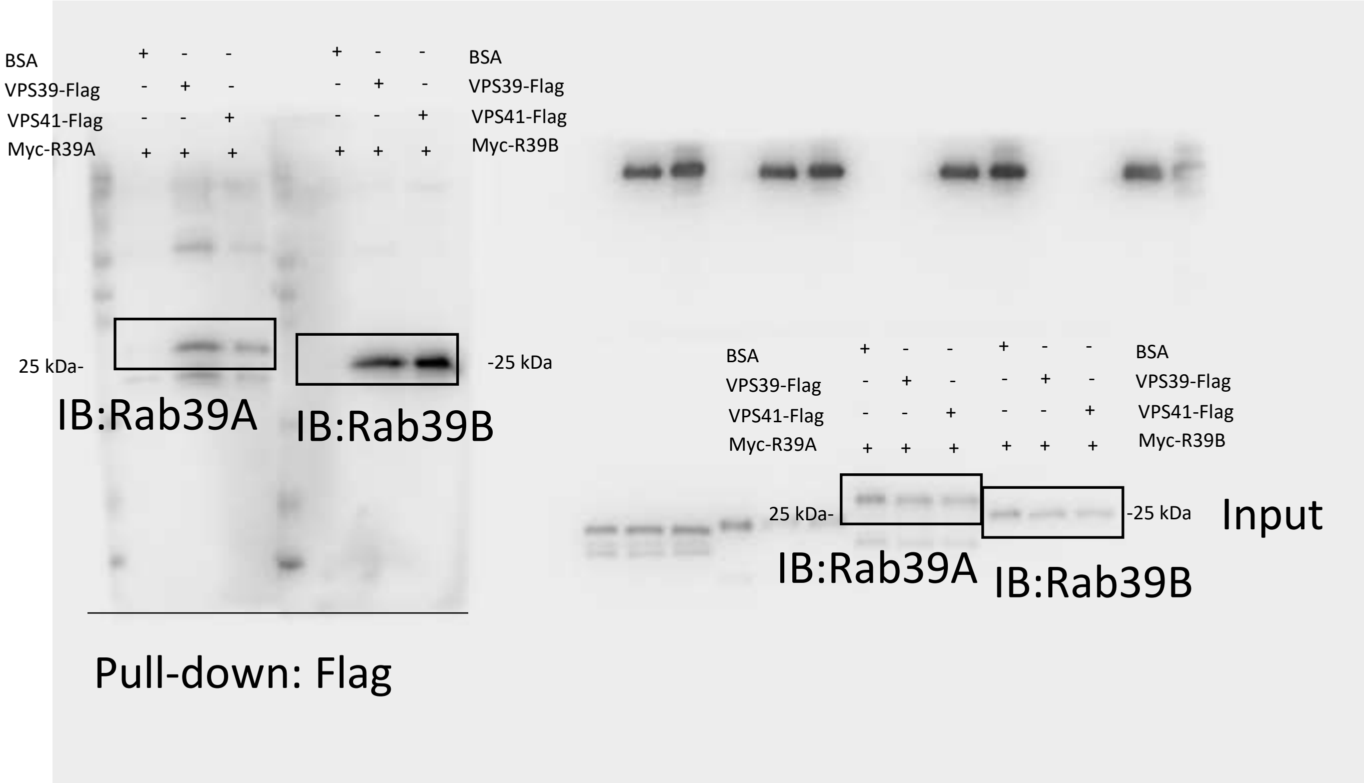

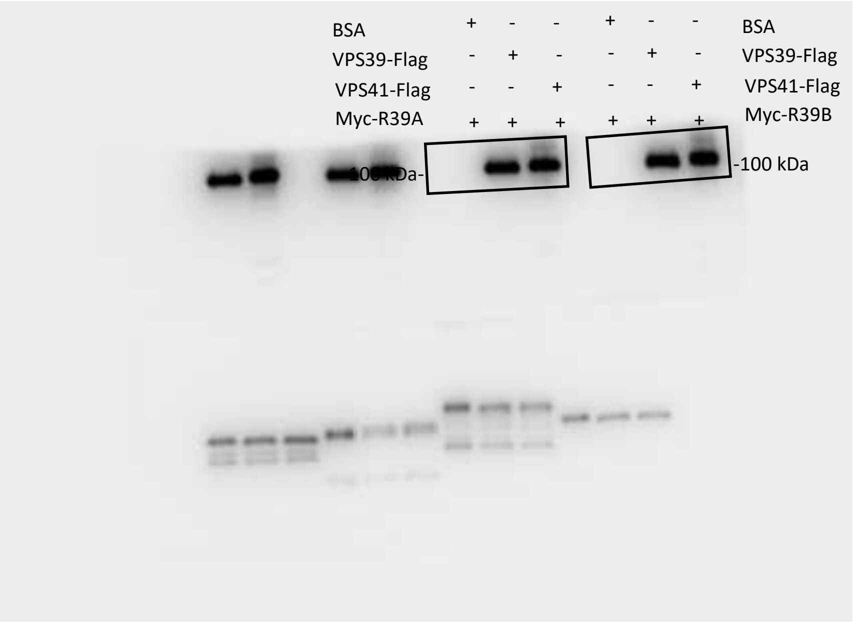

Pull-down: Flag

IB:Flag

Figure S5

a

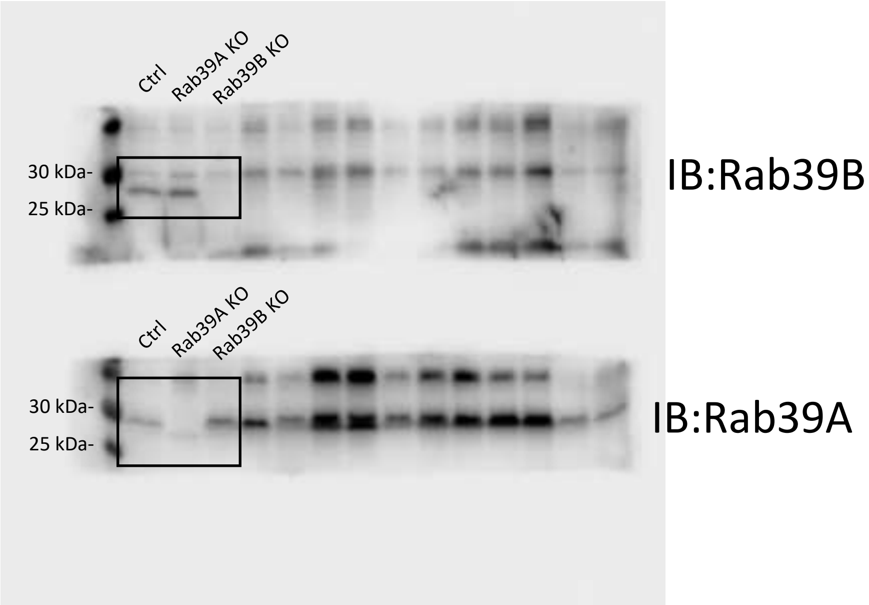

Ctrl
Rab39A KO
Rab39B KO
30 kDa-
25 kDa-
IB:Rab39B
Ctrl
Rab39A KO
Rab39B KO
30 kDa-
25 kDa-
IB:Rab39A

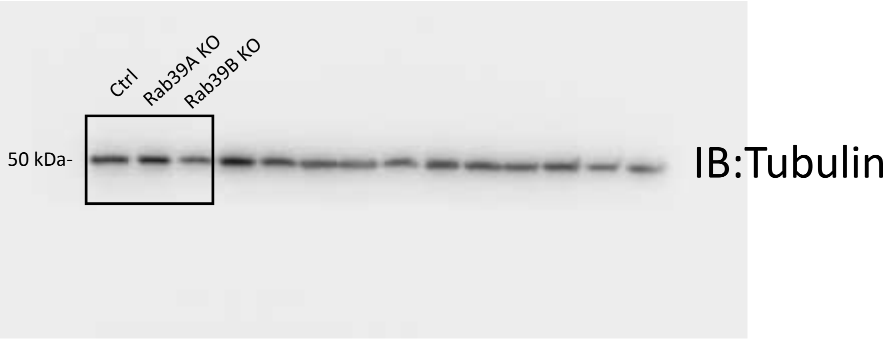

Ctrl
Rab39A KO
Rab39B KO
50 kDa-
IB:Tubulin

Figure S5j

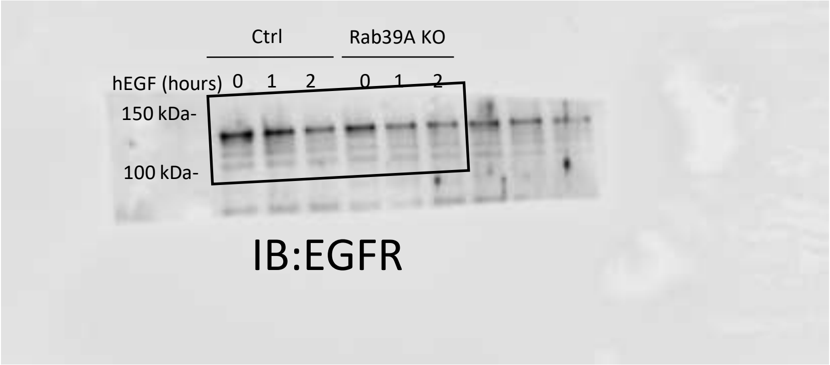

Ctrl
Rab39A KO
hEGF (hours) 0 1 2 0 1 2
150 kDa-
100 kDa-
IB:EGFR

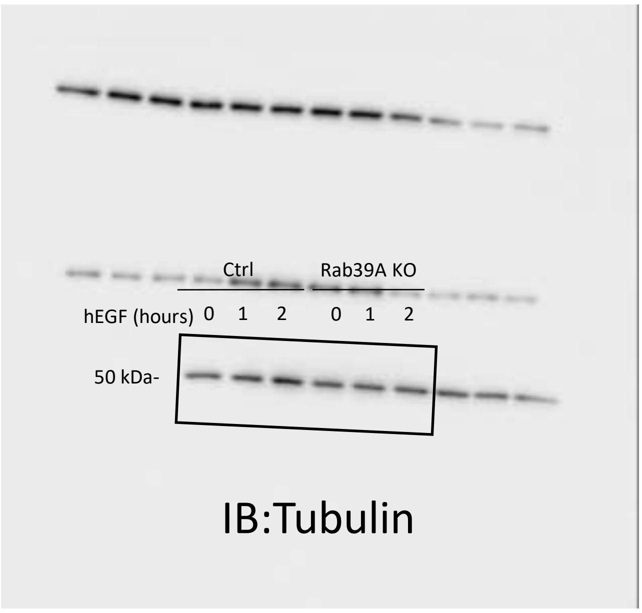

Ctrl
Rab39A KO
hEGF (hours) 0 1 2 0 1 2
50 kDa-
IB:Tubulin

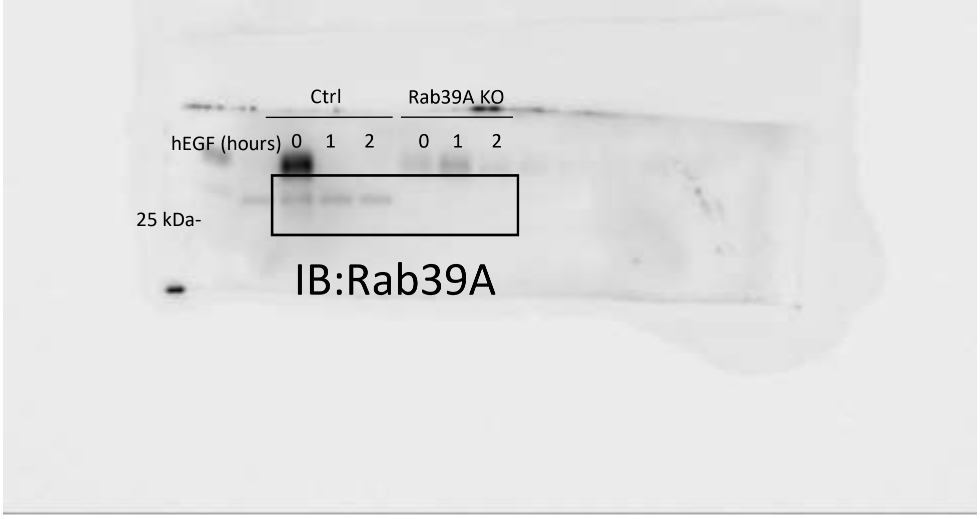

Ctrl
Rab39A KO
hEGF (hours) 0 1 2 0 1 2
25 kDa-
IB:Rab39A

Figure S5j

Results of independent repeats:

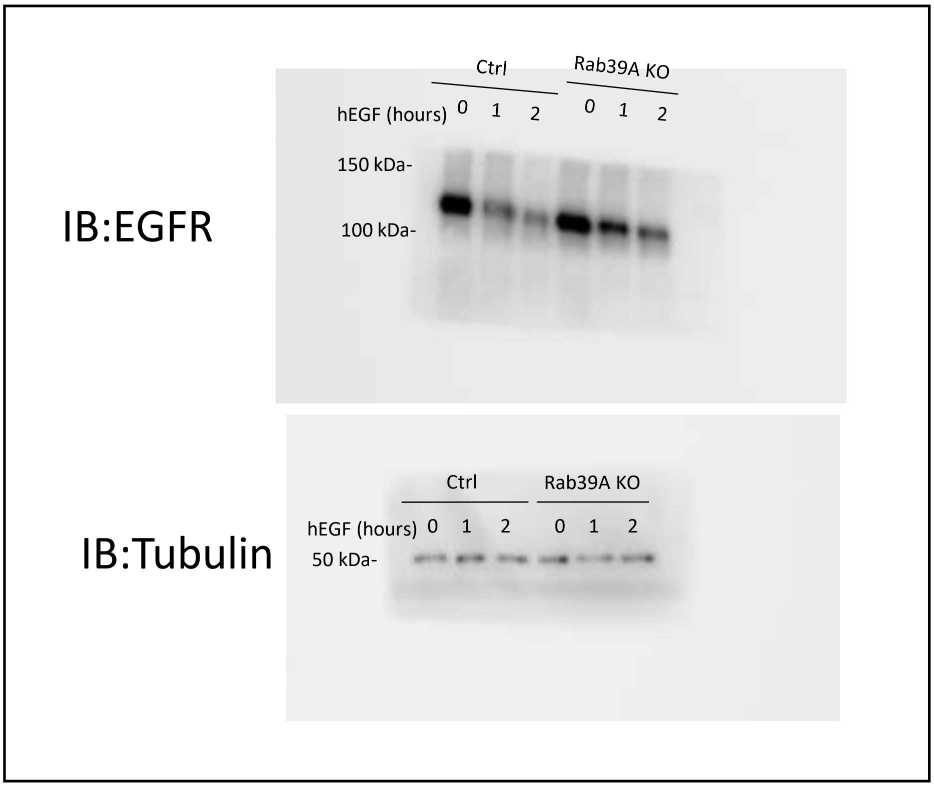

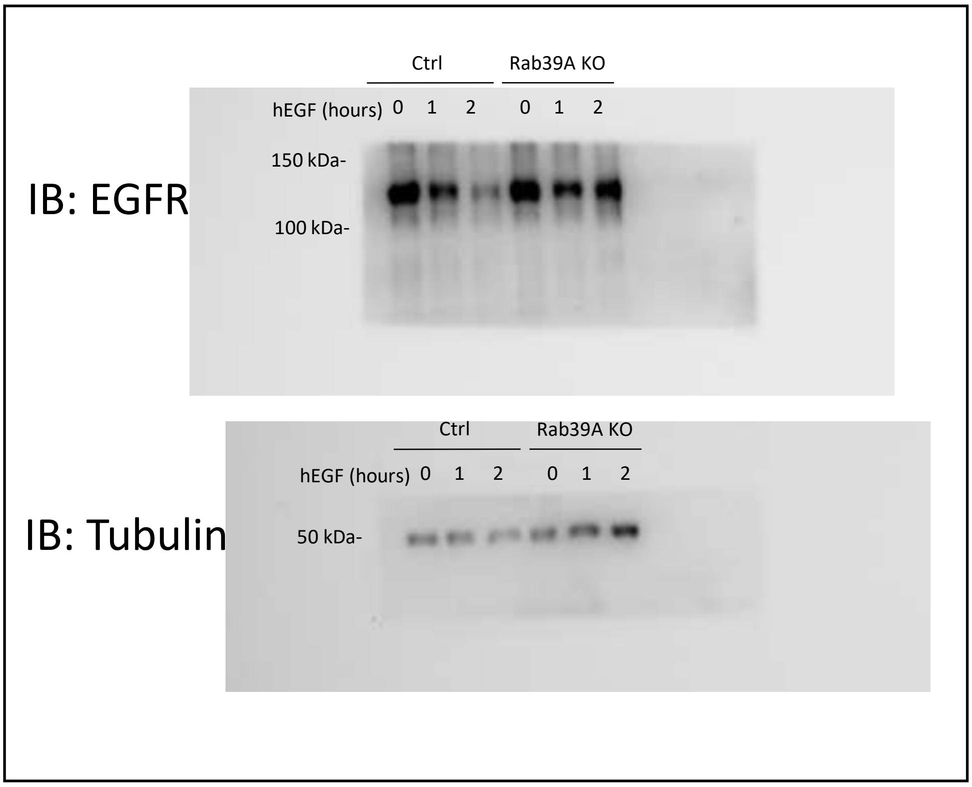

Figure S6 a

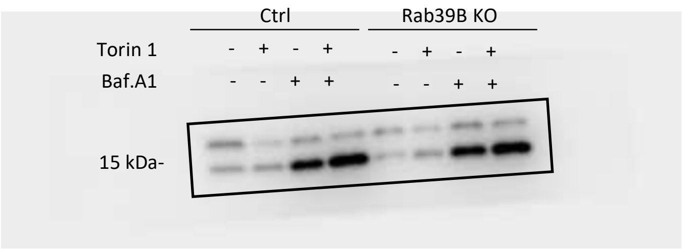

IB: LC3

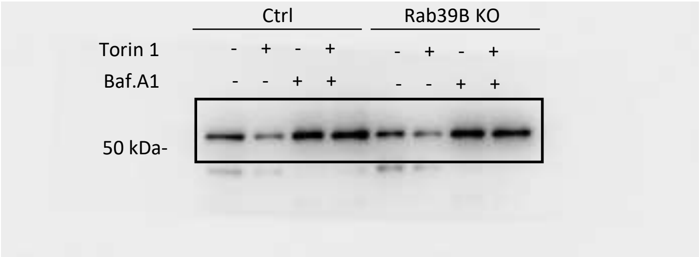

IB: p62

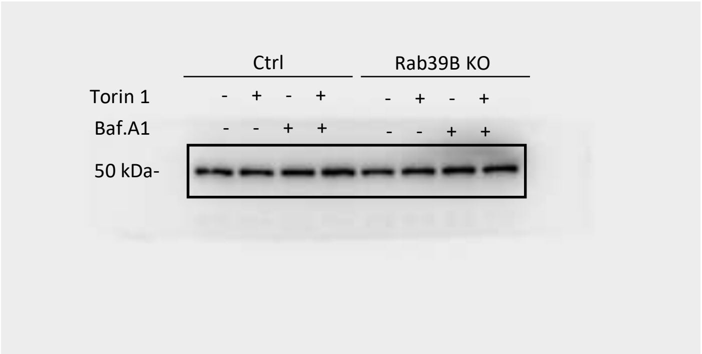

IB: Tubulin

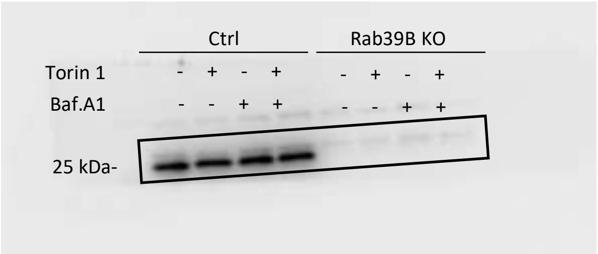

IB: Rab39B

Figure S6a

Results of independent repeats experiment:

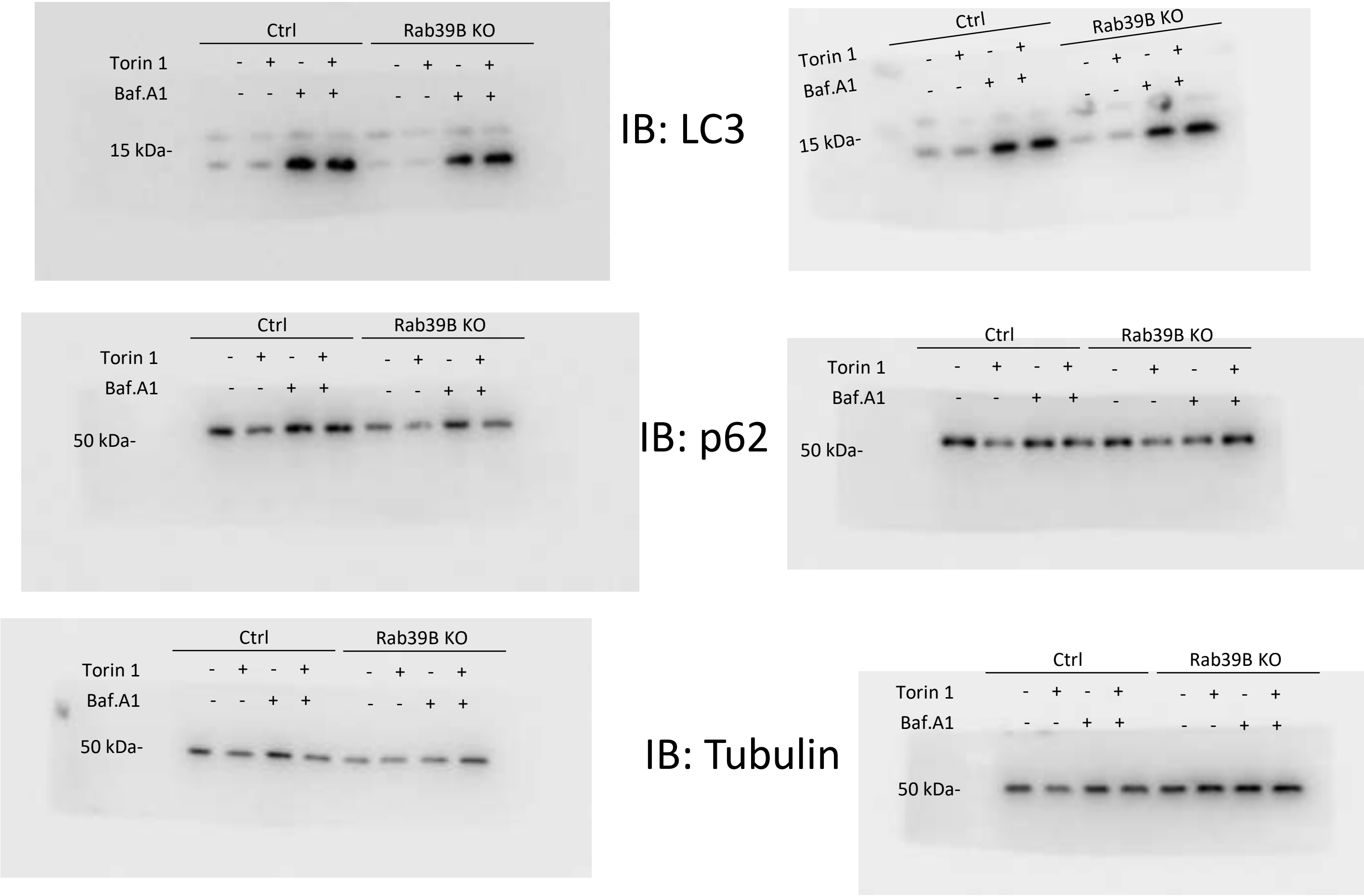

Figure S8a

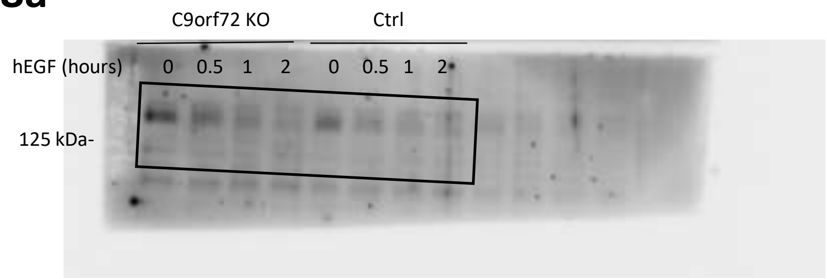

IB:EGFR

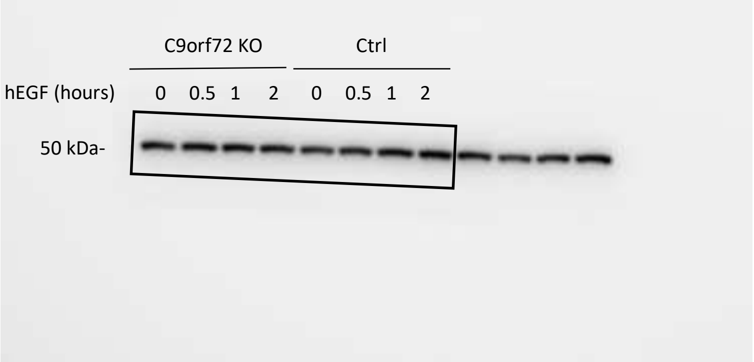

IB:Tubulin

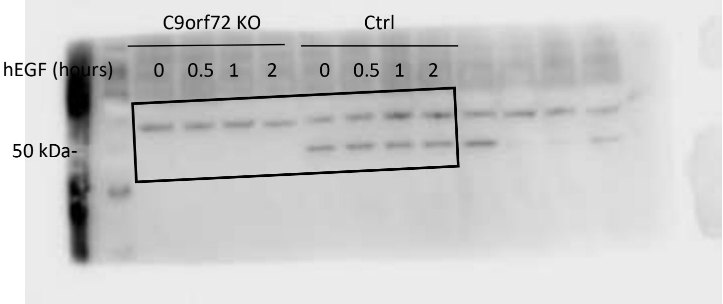

IB: C9orf72

Results of independent repeats:

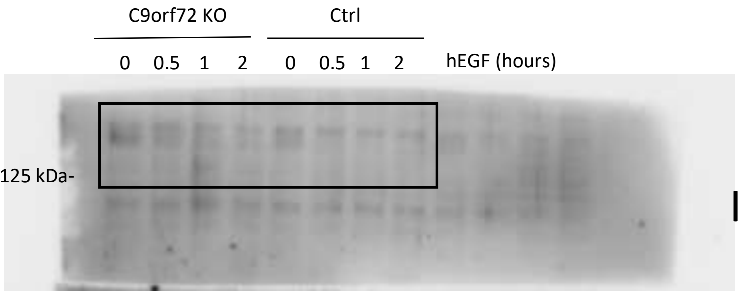

IB:EGFR

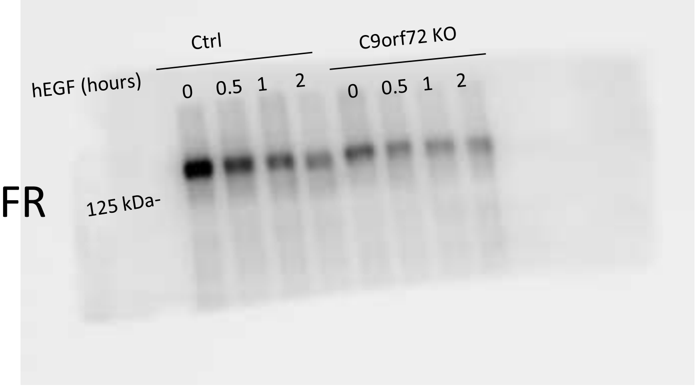

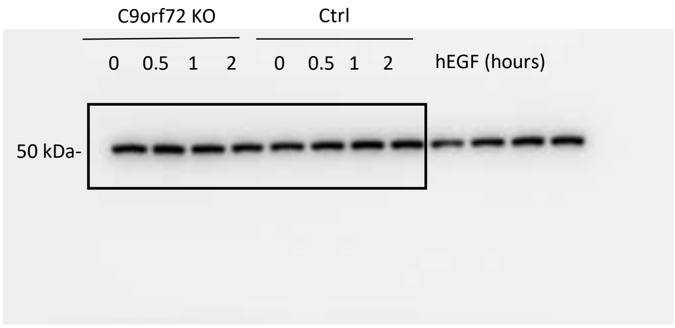

IB:Tubulin

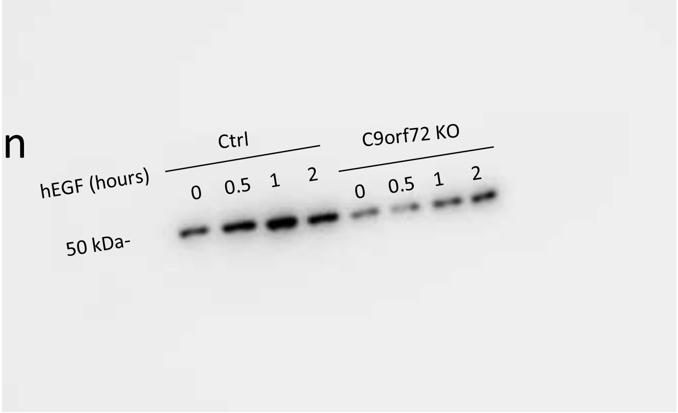

Supplement: Supplementary file 1 — Supplementary Information [file 41467_2023_42003_MOESM1_ESM.pdf]
