# Supplementary material for: C9orf72-catalyzed GTP loading of Rab39A enables HOPS-mediated membrane tethering and fusion in mammalian autophagy
Source: Nat Commun. 2023 Oct 11;14:6360. doi: 10.1038/s41467-023-42003-0 (PMC10567733; doi:10.1038/s41467-023-42003-0)
Supplement: Supplementary file 2 — Description of additional supplementary files [file 41467_2023_42003_MOESM2_ESM.pdf]

<u>Description of additional Supplementary Files</u>

Supplementary Data 1 : Results of tandem affinity purification-mass spectrometry assay for STX17 in 293T cells.
