# Supplementary material for: C9orf72-catalyzed GTP loading of Rab39A enables HOPS-mediated membrane tethering and fusion in mammalian autophagy
Source: Nat Commun. 2023 Oct 11;14:6360. doi: 10.1038/s41467-023-42003-0 (PMC10567733; doi:10.1038/s41467-023-42003-0)

Figure 1a

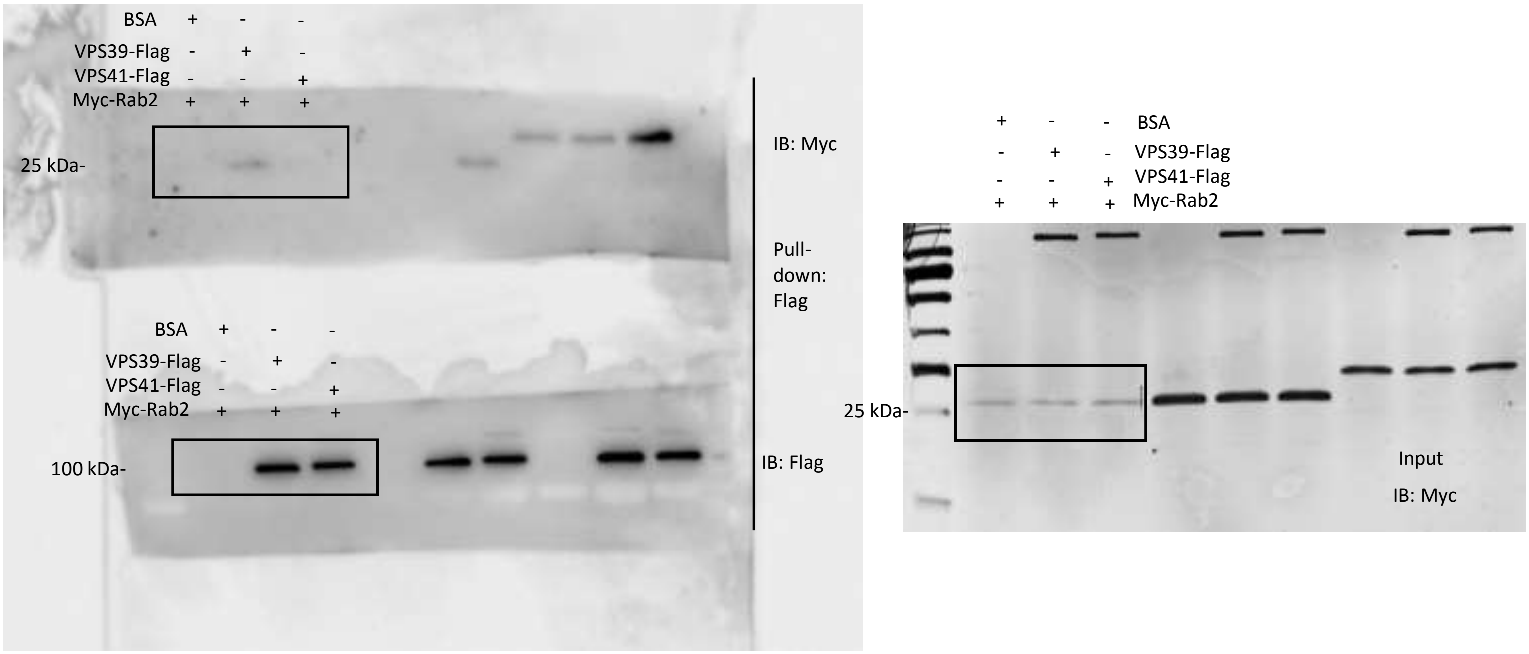

Figure 1b

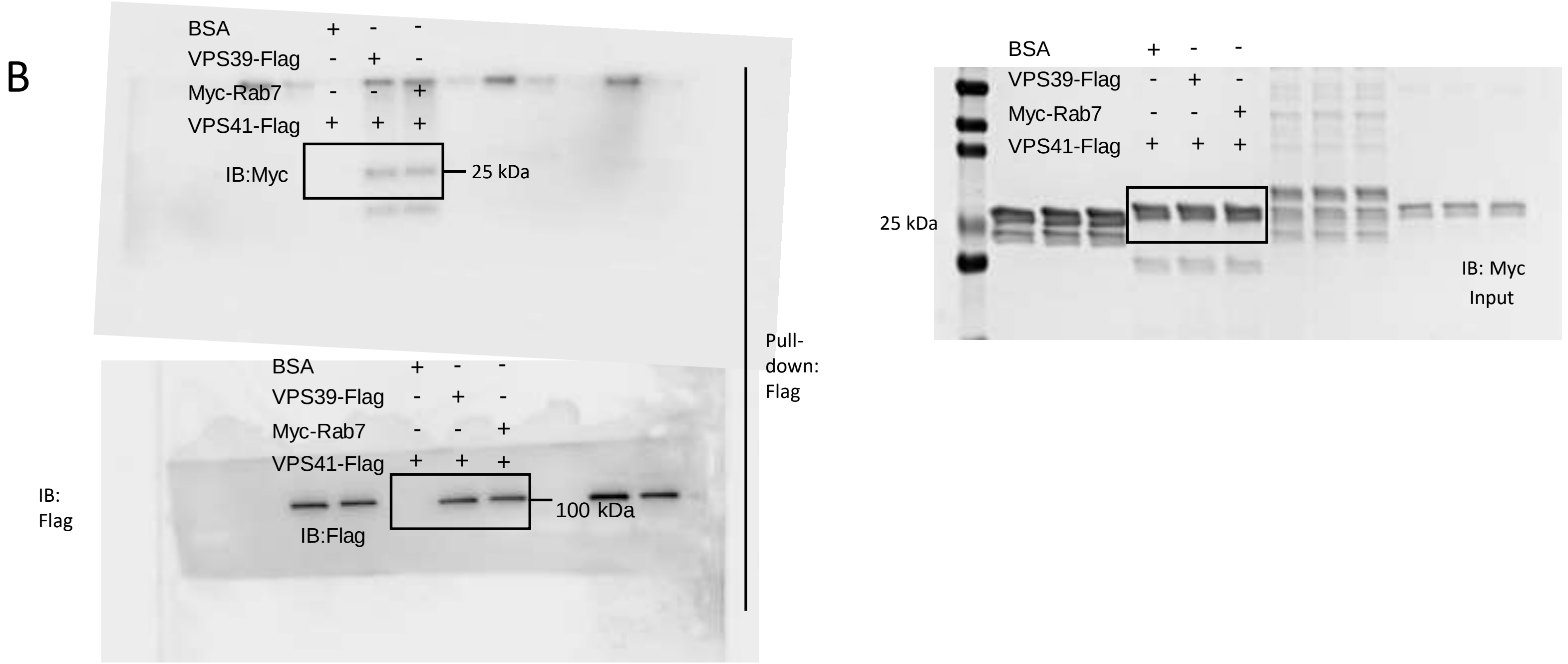

Figure 1f

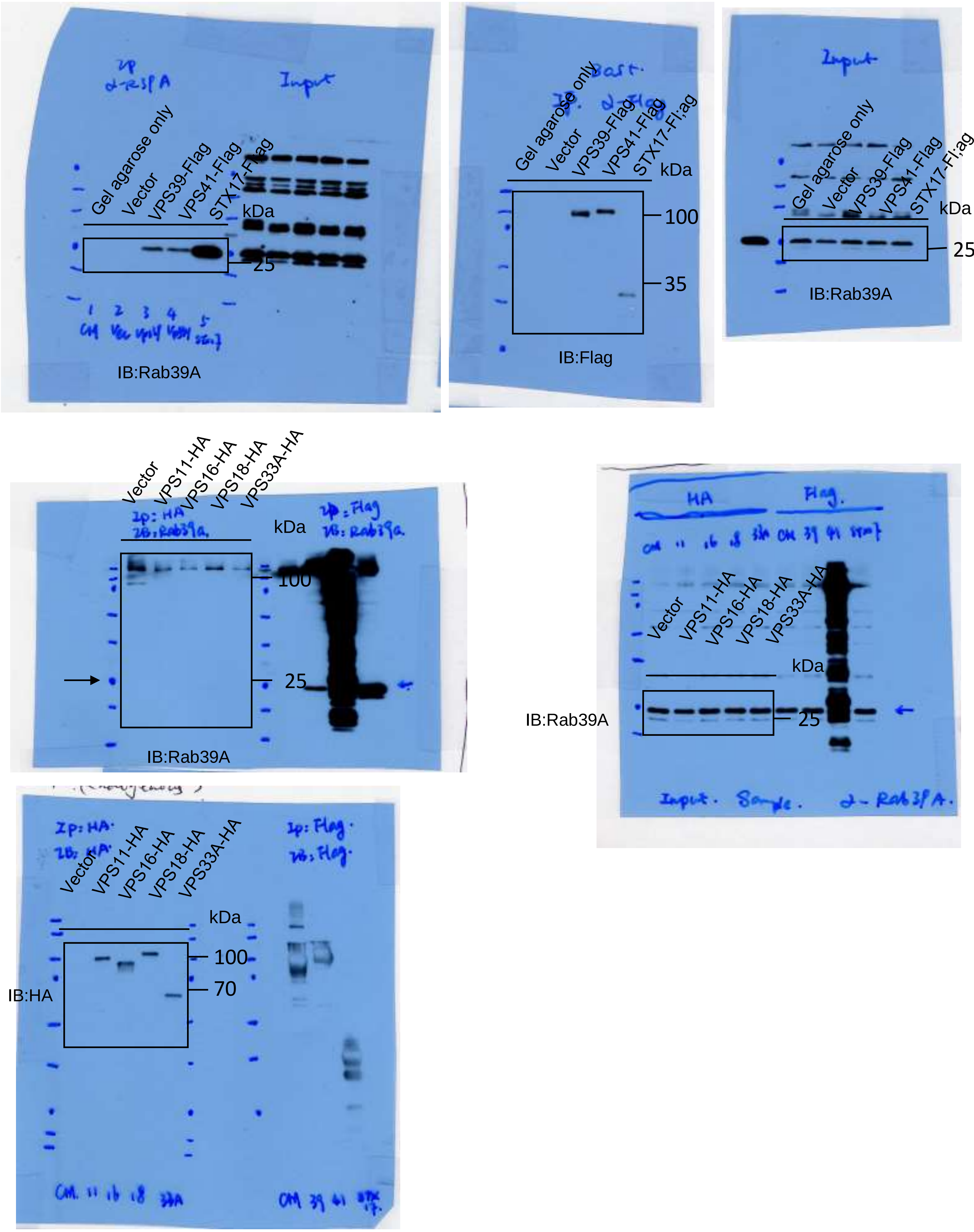
Gel agarose only
Vector
VPS39-Flag
VPS41-Flag
STX17-Flag
kDa
25
IB:Rab39A
Gel agarose only
Vector
VPS39-Flag
VPS41-Flag
STX17-Fl;ag
kDa
100
35
IB:Flag
Gel agarose only
Vector
VPS39-Flag
VPS41-Flag
STX17-Fl;ag
kDa
25
IB:Rab39A
Vector
VPS11-HA
VPS16-HA
VPS18-HA
VPS33A-HA
kDa
100
25
IB:Rab39A
Vector
VPS11-HA
VPS16-HA
VPS18-HA
VPS33A-HA
kDa
25
IB:Rab39A
Vector
VPS11-HA
VPS16-HA
VPS18-HA
VPS33A-HA
kDa
100
70
IB:HA

Figure 1g

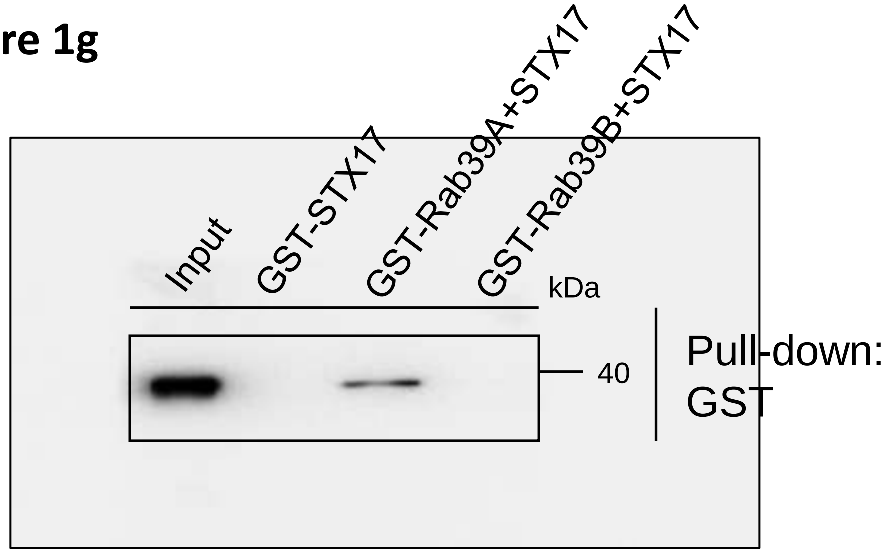
Input
GST-STX17
GST-Rab39A+STX17
GST-Rab39B+STX17
kDa
40
Pull-down:
GST

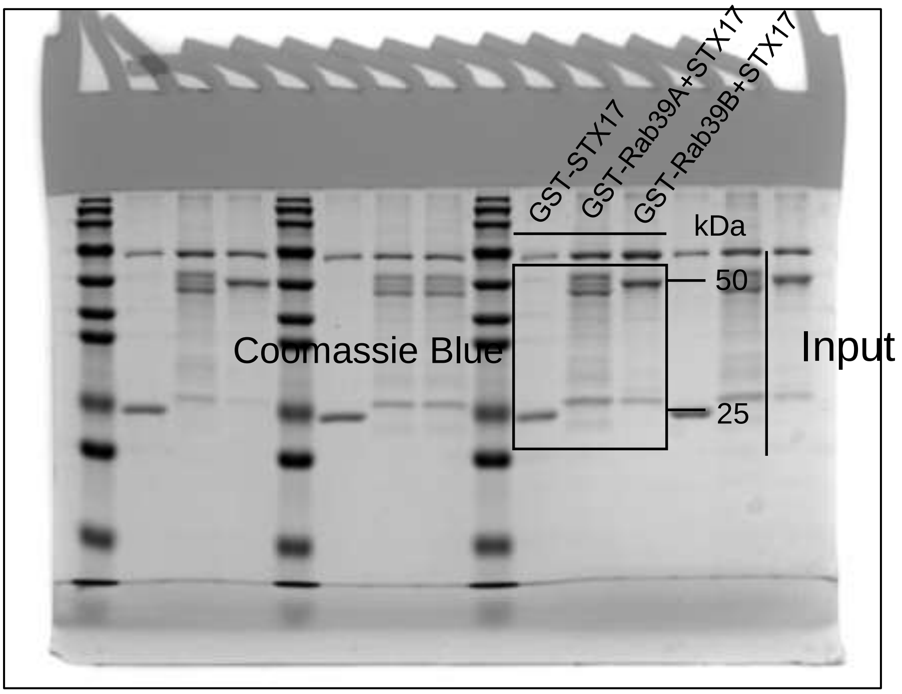
GST-STX17
GST-Rab39A+STX17
GST-Rab39B+STX17
kDa
50
25
Coomassie Blue
Input

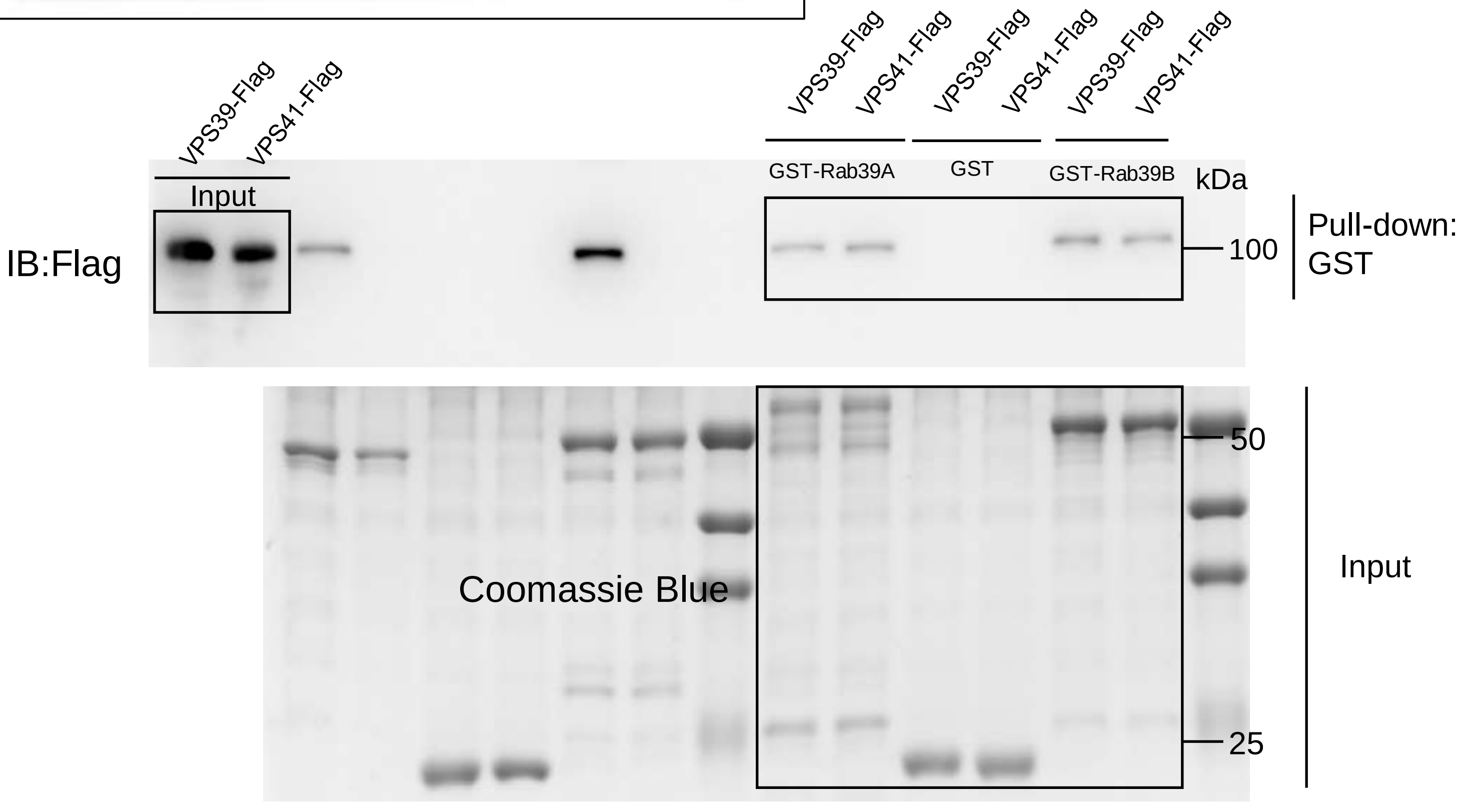
VPS39-Flag
VPS41-Flag
Input
VPS39-Flag
VPS41-Flag
VPS39-Flag
VPS41-Flag
VPS39-Flag
VPS41-Flag
GST-Rab39A
GST
GST-Rab39B
kDa
IB:Flag
100
Pull-down:
GST
Coomassie Blue
50
25
Input

Figure 2a

IB:LC3

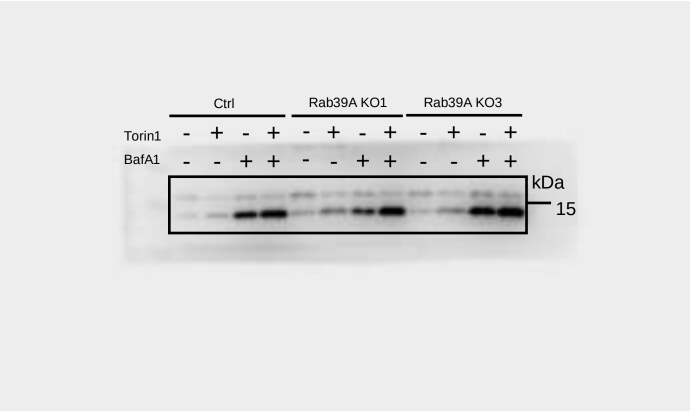

IB: p62

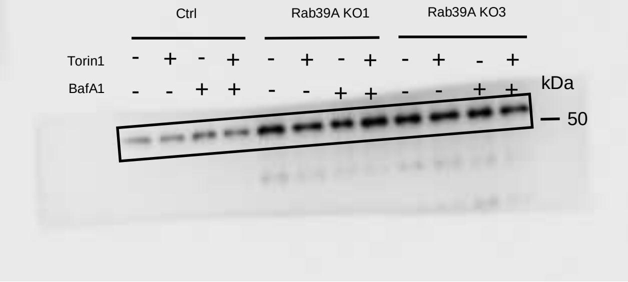

IB: Tubulin

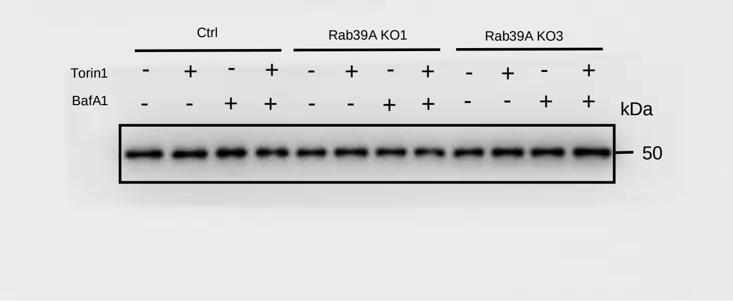

IB: Rab39A

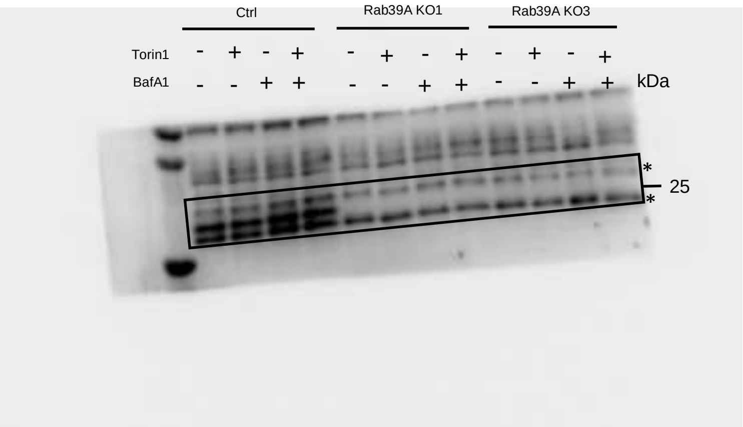

Figure 2a

Results of independent repeats:

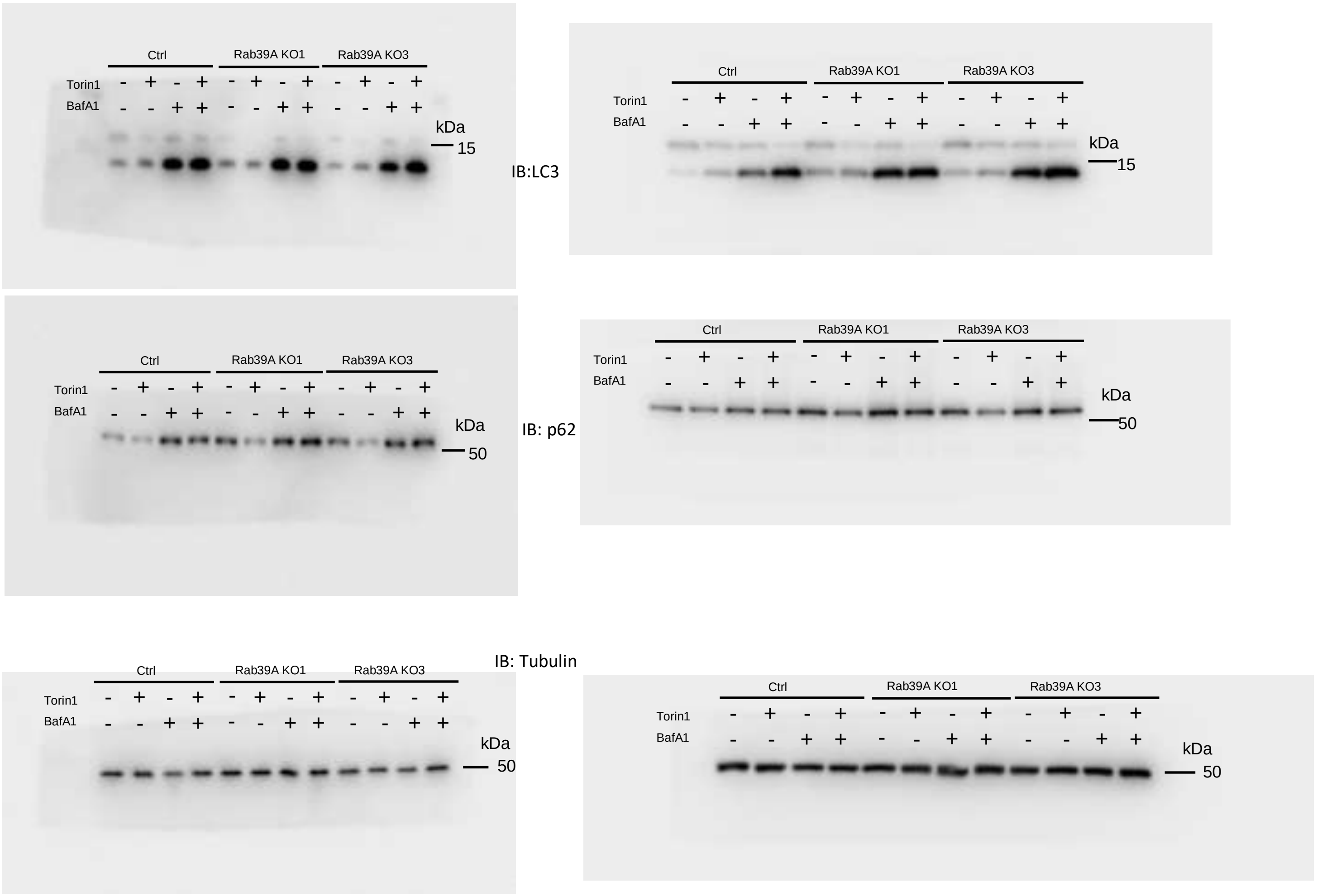

Figure 2i

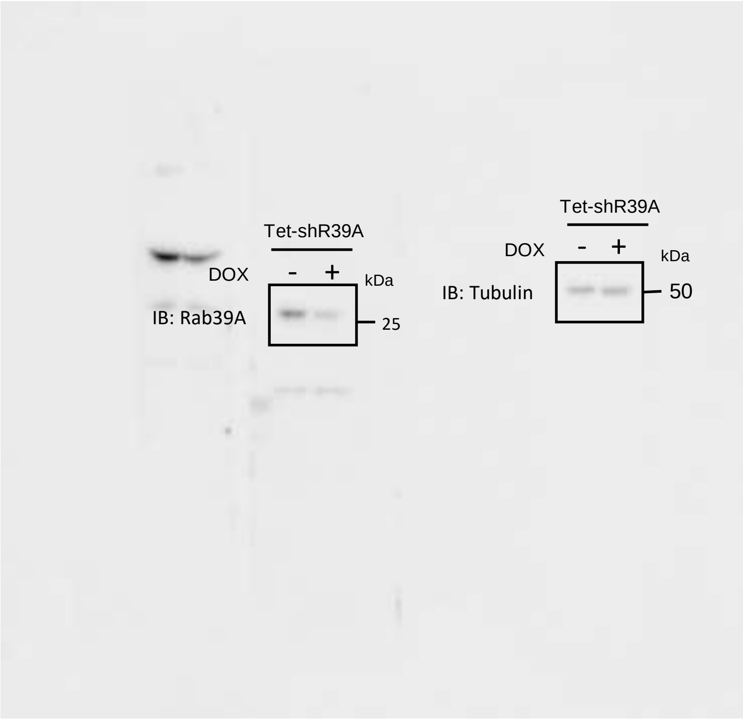
Tet-shR39A
DOX
-
+
kDa
IB: Rab39A
25
Tet-shR39A
DOX
-
+
kDa
IB: Tubulin
50

Figure 3a

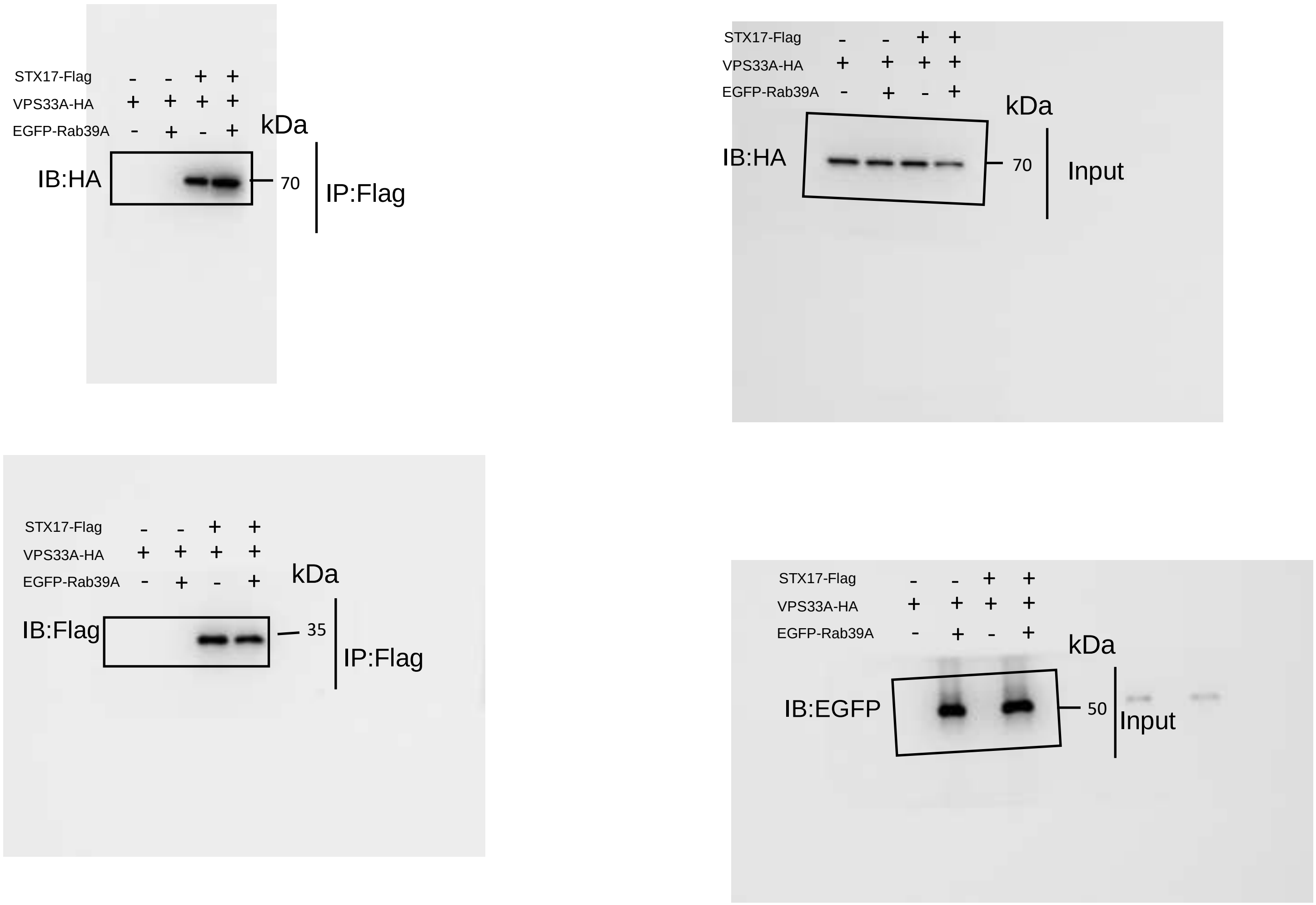
STX17-Flag - - + +
VPS33A-HA + + + +
EGFP-Rab39A - + - +
kDa
IB:HA
70
IP:Flag
STX17-Flag - - + +
VPS33A-HA + + + +
EGFP-Rab39A - + - +
kDa
IB:HA
70
Input
STX17-Flag - - + +
VPS33A-HA + + + +
EGFP-Rab39A - + - +
kDa
IB:Flag
35
IP:Flag
STX17-Flag - - + +
VPS33A-HA + + + +
EGFP-Rab39A - + - +
kDa
IB:EGFP
50
Input

Figure 3a

Results of independent repeats experiment:

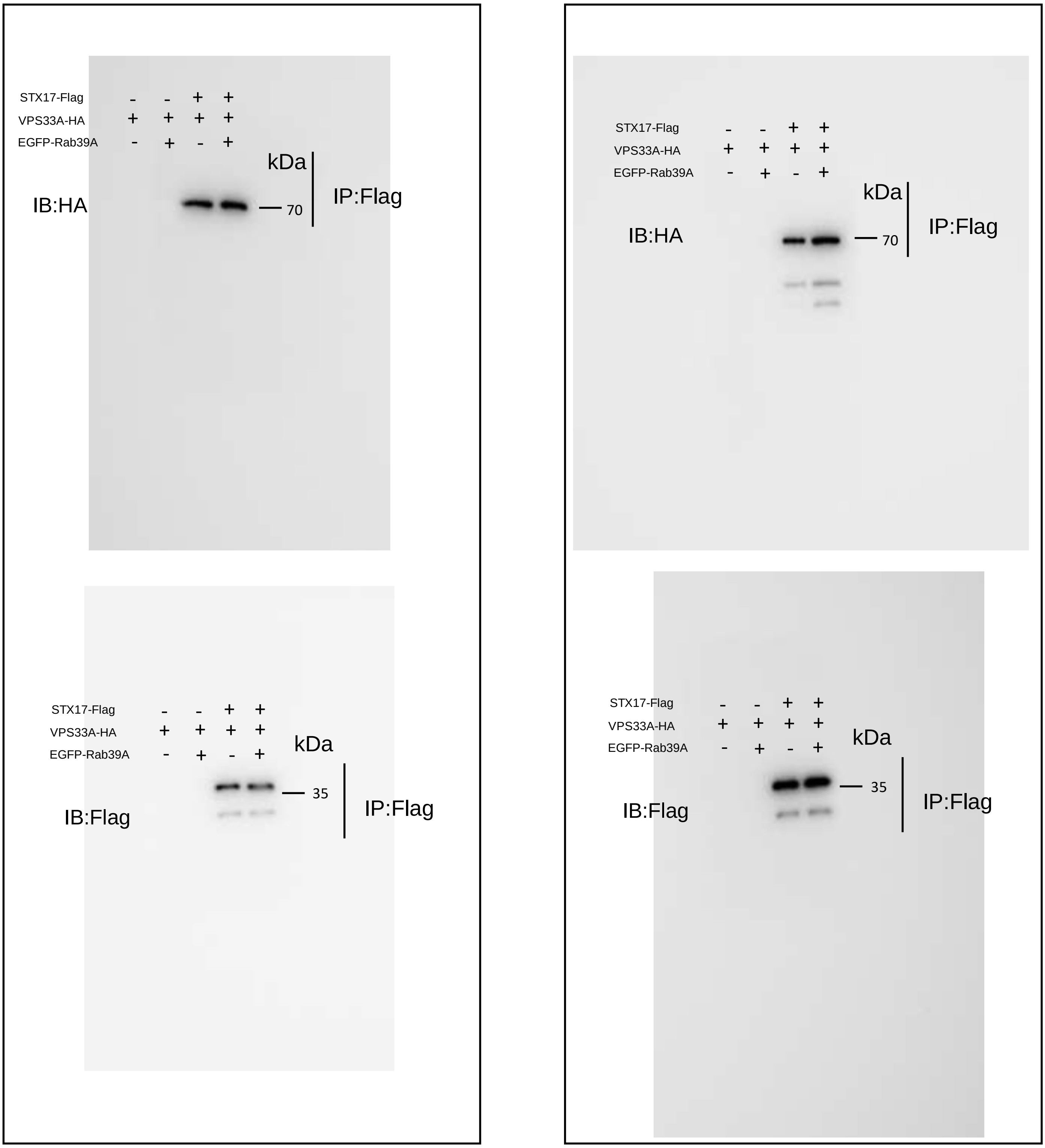

Figure3b

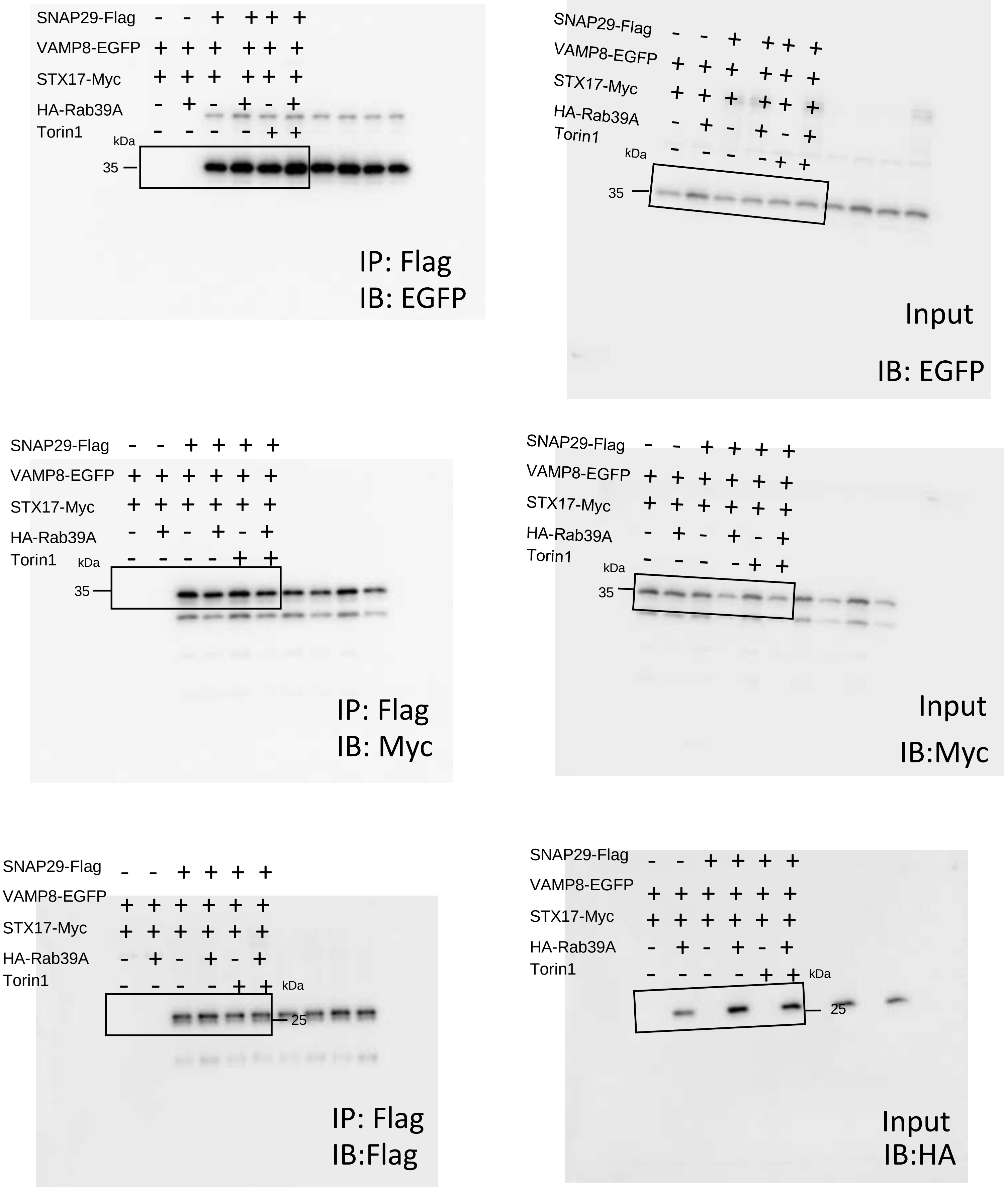
SNAP29-Flag - - + + + +
VAMP8-EGFP + + + + + +
STX17-Myc + + + + + +
HA-Rab39A - + - + - +
Torin1 - - - - + +
kDa
35
IP: Flag
IB: EGFP
SNAP29-Flag - - + + + +
VAMP8-EGFP + + + + + +
STX17-Myc + + + + + +
HA-Rab39A - + - + - +
Torin1 - - - - + +
kDa
35
Input
IB: EGFP
SNAP29-Flag - - + + + +
VAMP8-EGFP + + + + + +
STX17-Myc + + + + + +
HA-Rab39A - + - + - +
Torin1 - - - - + +
kDa
35
IP: Flag
IB: Myc
SNAP29-Flag - - + + + +
VAMP8-EGFP + + + + + +
STX17-Myc + + + + + +
HA-Rab39A - + - + - +
Torin1 - - - - + +
kDa
35
Input
IB:Myc
SNAP29-Flag - - + + + +
VAMP8-EGFP + + + + + +
STX17-Myc + + + + + +
HA-Rab39A - + - + - +
Torin1 - - - - + +
kDa
25
IP: Flag
IB:Flag
SNAP29-Flag - - + + + +
VAMP8-EGFP + + + + + +
STX17-Myc + + + + + +
HA-Rab39A - + - + - +
Torin1 - - - - + +
kDa
25
Input
IB:HA

Figure3b

Results of independent repeats experiment:

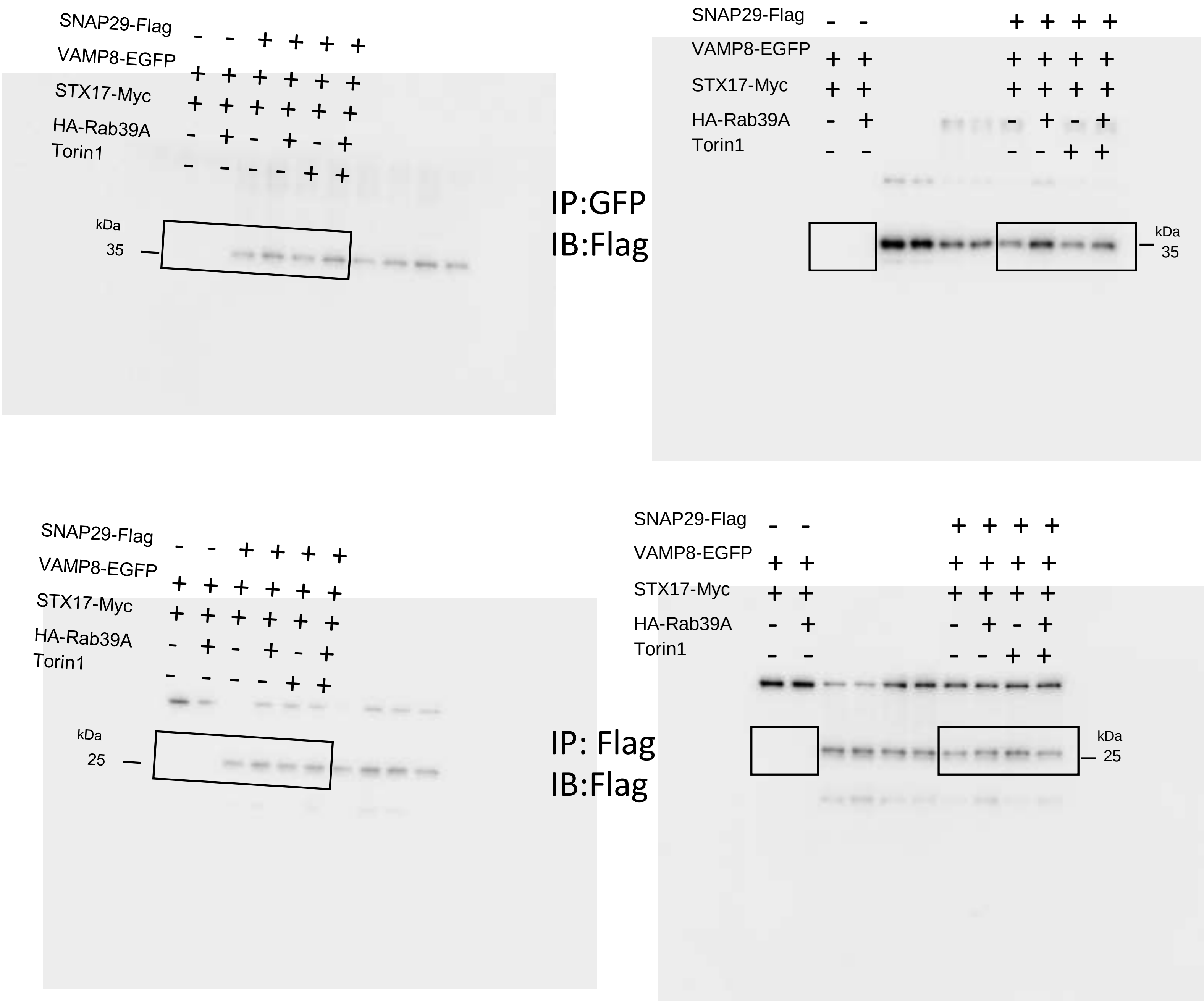

Figure 3c

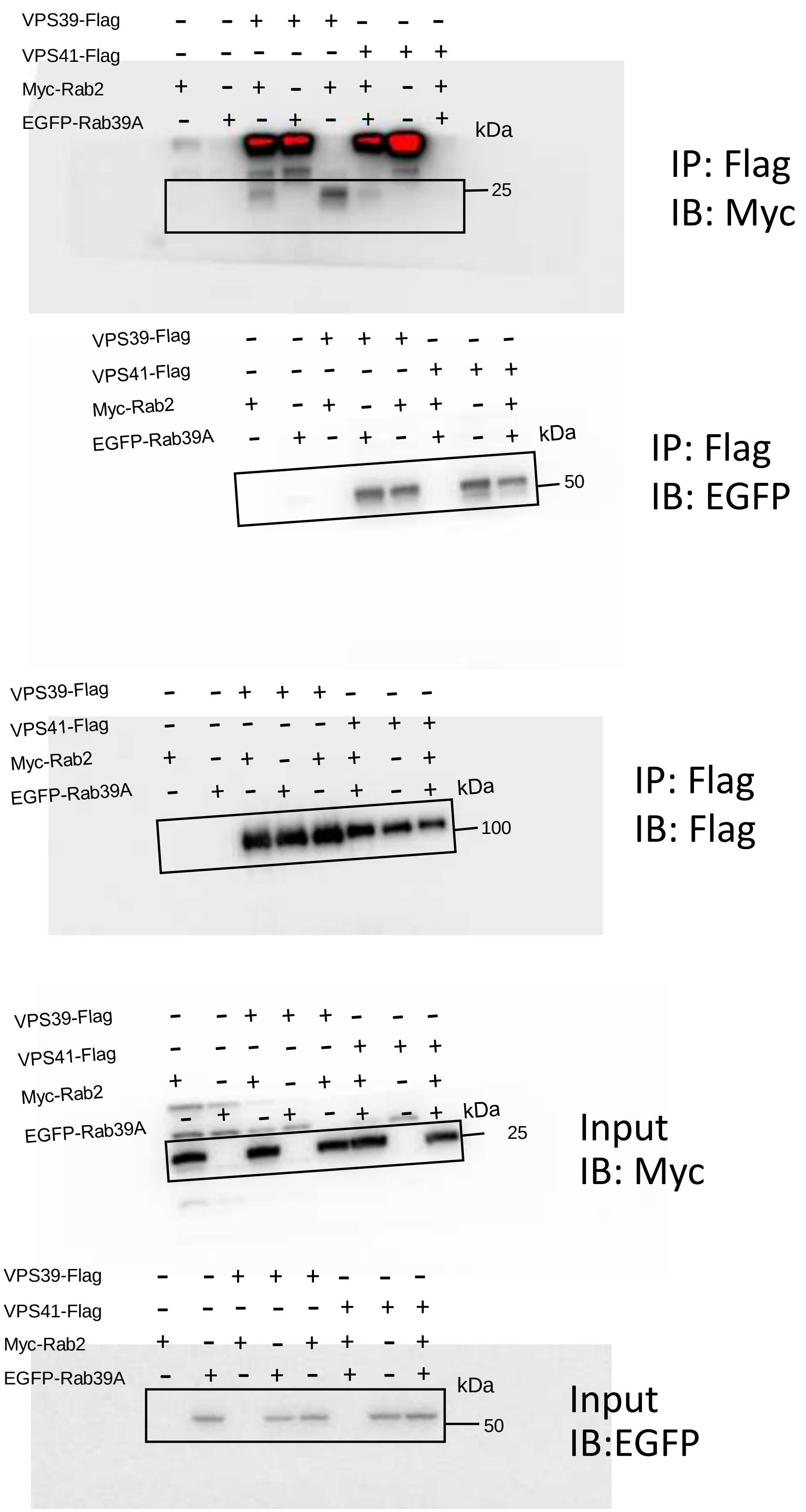
VPS39-Flag – – + + + – – –
VPS41-Flag – – – – – + + +
Myc-Rab2 + – + – + + – +
EGFP-Rab39A – + – + – + – +
kDa
25
IP: Flag
IB: Myc
VPS39-Flag – – + + + – – –
VPS41-Flag – – – – – + + +
Myc-Rab2 + – + – + + – +
EGFP-Rab39A – + – + – + – +
kDa
50
IP: Flag
IB: EGFP
VPS39-Flag – – + + + – – –
VPS41-Flag – – – – – + + +
Myc-Rab2 + – + – + + – +
EGFP-Rab39A – + – + – + – +
kDa
100
IP: Flag
IB: Flag
VPS39-Flag – – + + + – – –
VPS41-Flag – – – – – + + +
Myc-Rab2 + – + – + + – +
EGFP-Rab39A – + – + – + – +
kDa
25
Input
IB: Myc
VPS39-Flag – – + + + – – –
VPS41-Flag – – – – – + + +
Myc-Rab2 + – + – + + – +
EGFP-Rab39A – + – + – + – +
kDa
50
Input
IB:EGFP

Figure 3c

Results of independent repeats experiment:

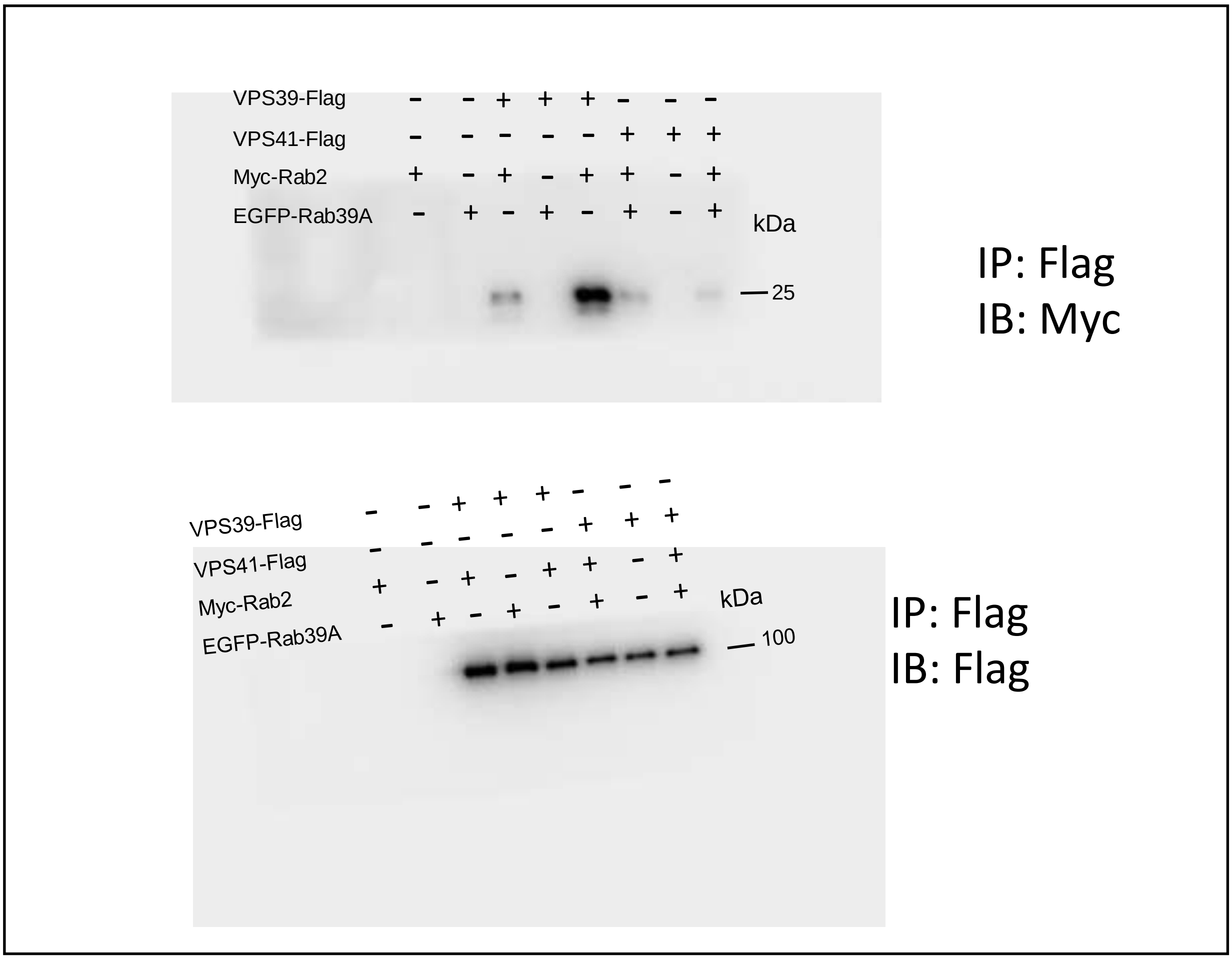

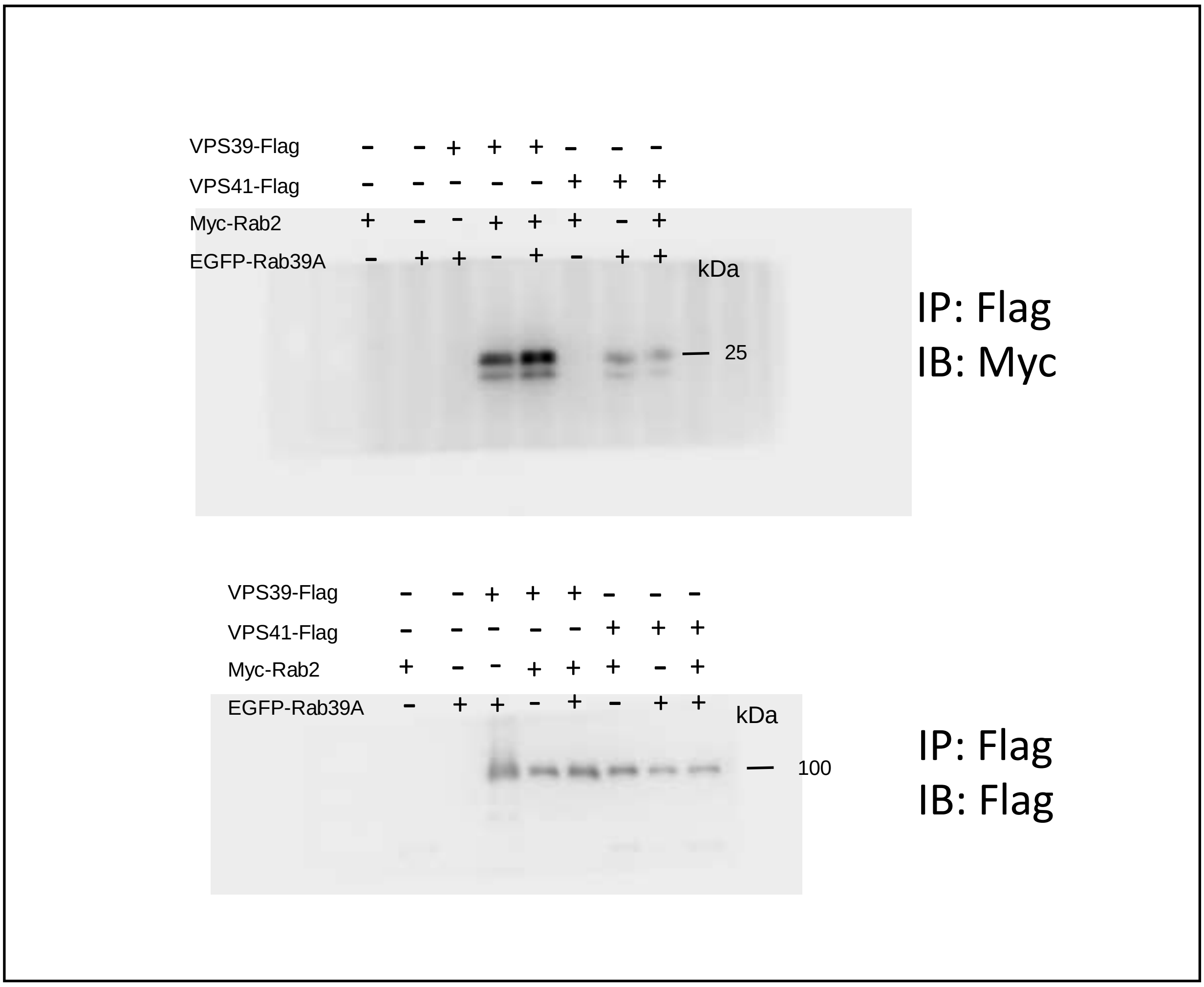

Figure 3d

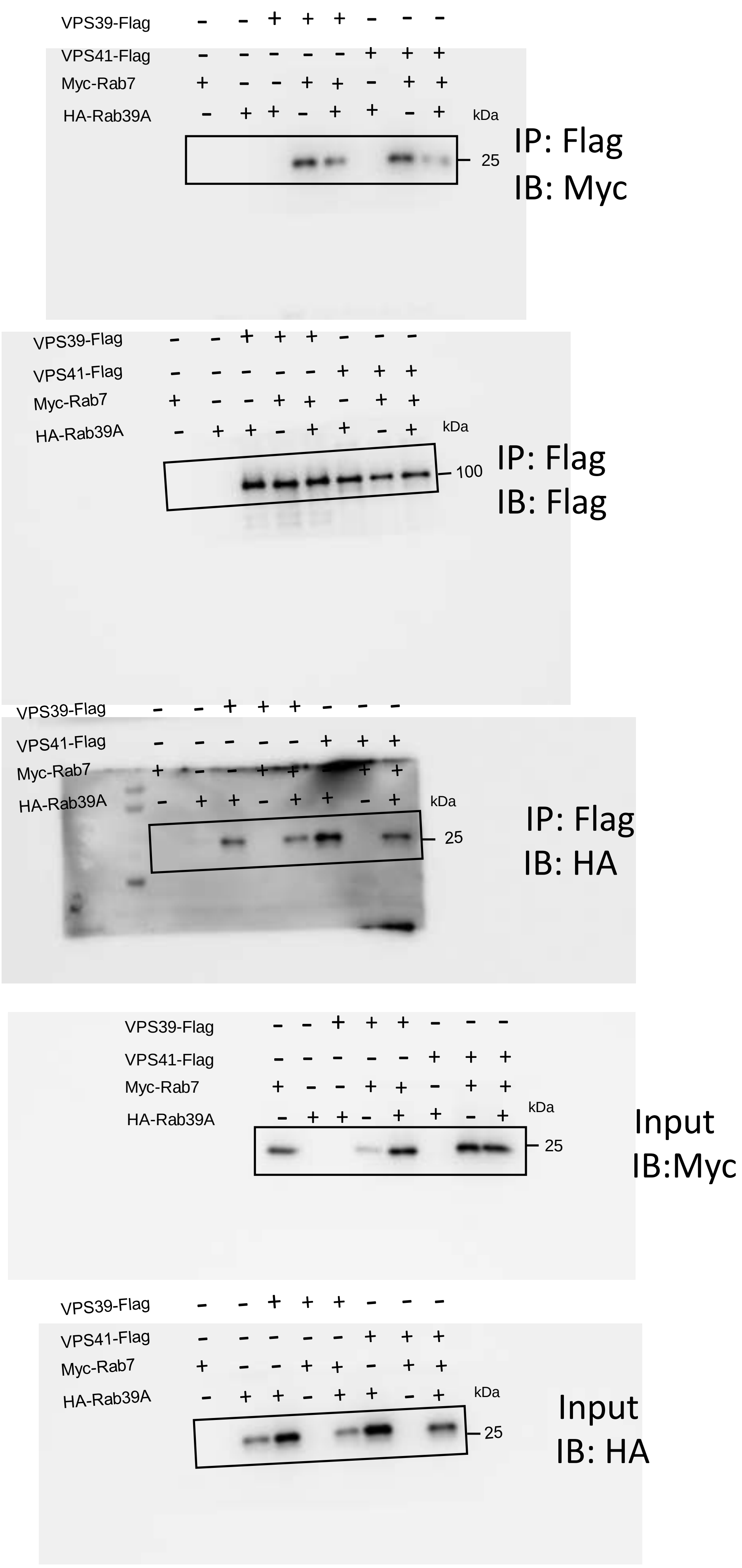
VPS39-Flag – – + + + – – –
VPS41-Flag – – – – – + + +
Myc-Rab7 + – – + + – + +
HA-Rab39A – + + – + + – +
kDa
25
IP: Flag
IB: Myc
VPS39-Flag – – + + + – – –
VPS41-Flag – – – – – + + +
Myc-Rab7 + – – + + – + +
HA-Rab39A – + + – + + – +
kDa
100
IP: Flag
IB: Flag
VPS39-Flag – – + + + – – –
VPS41-Flag – – – – – + + +
Myc-Rab7 + – – + + – + +
HA-Rab39A – + + – + + – +
kDa
25
IP: Flag
IB: HA
VPS39-Flag – – + + + – – –
VPS41-Flag – – – – – + + +
Myc-Rab7 + – – + + – + +
HA-Rab39A – + + – + + – +
kDa
25
Input
IB:Myc
VPS39-Flag – – + + + – – –
VPS41-Flag – – – – – + + +
Myc-Rab7 + – – + + – + +
HA-Rab39A – + + – + + – +
kDa
25
Input
IB: HA

Figure 3d

Results of independent repeats:

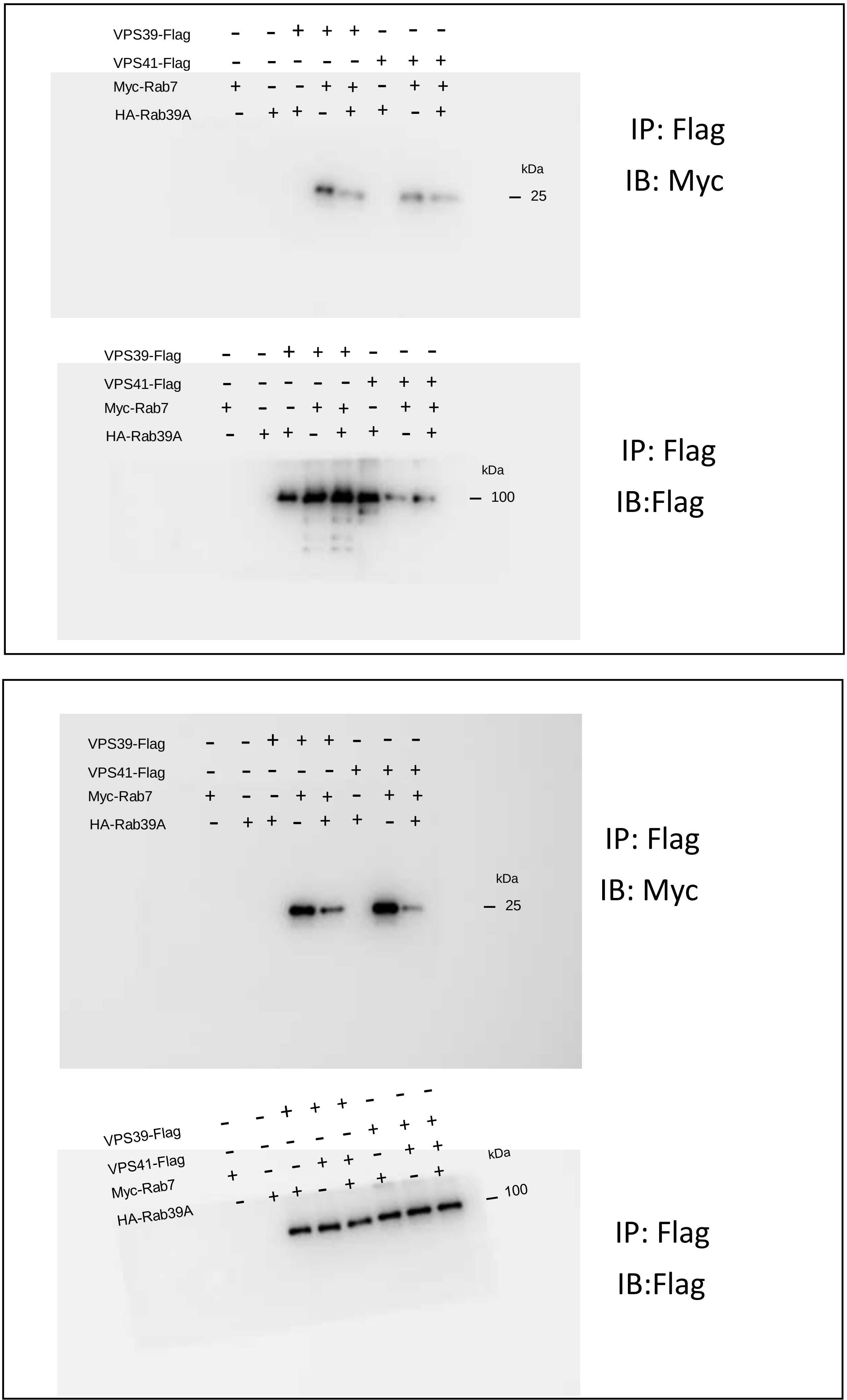

Figure 5a

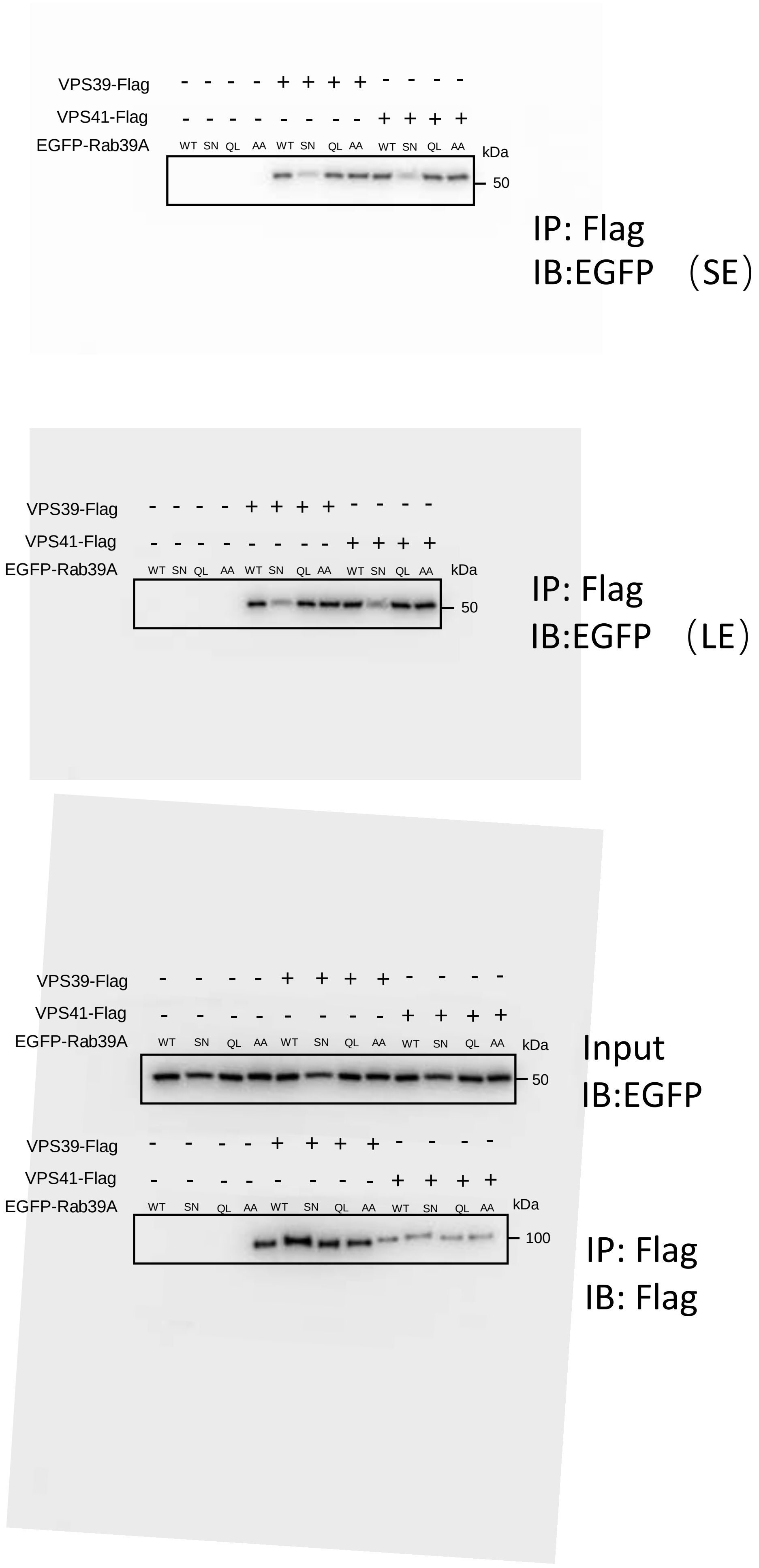
VPS39-Flag - - - - + + + + - - - -
VPS41-Flag - - - - - - - - + + + +
EGFP-Rab39A WT SN QL AA WT SN QL AA WT SN QL AA
kDa
50
IP: Flag
IB:EGFP （SE）
VPS39-Flag - - - - + + + + - - - -
VPS41-Flag - - - - - - - - + + + +
EGFP-Rab39A WT SN QL AA WT SN QL AA WT SN QL AA
kDa
50
IP: Flag
IB:EGFP （LE）
VPS39-Flag - - - - + + + + - - - -
VPS41-Flag - - - - - - - - + + + +
EGFP-Rab39A WT SN QL AA WT SN QL AA WT SN QL AA
kDa
50
Input
IB:EGFP
VPS39-Flag - - - - + + + + - - - -
VPS41-Flag - - - - - - - - + + + +
EGFP-Rab39A WT SN QL AA WT SN QL AA WT SN QL AA
kDa
100
IP: Flag
IB: Flag

Figure 5a

Results of independent repeats:

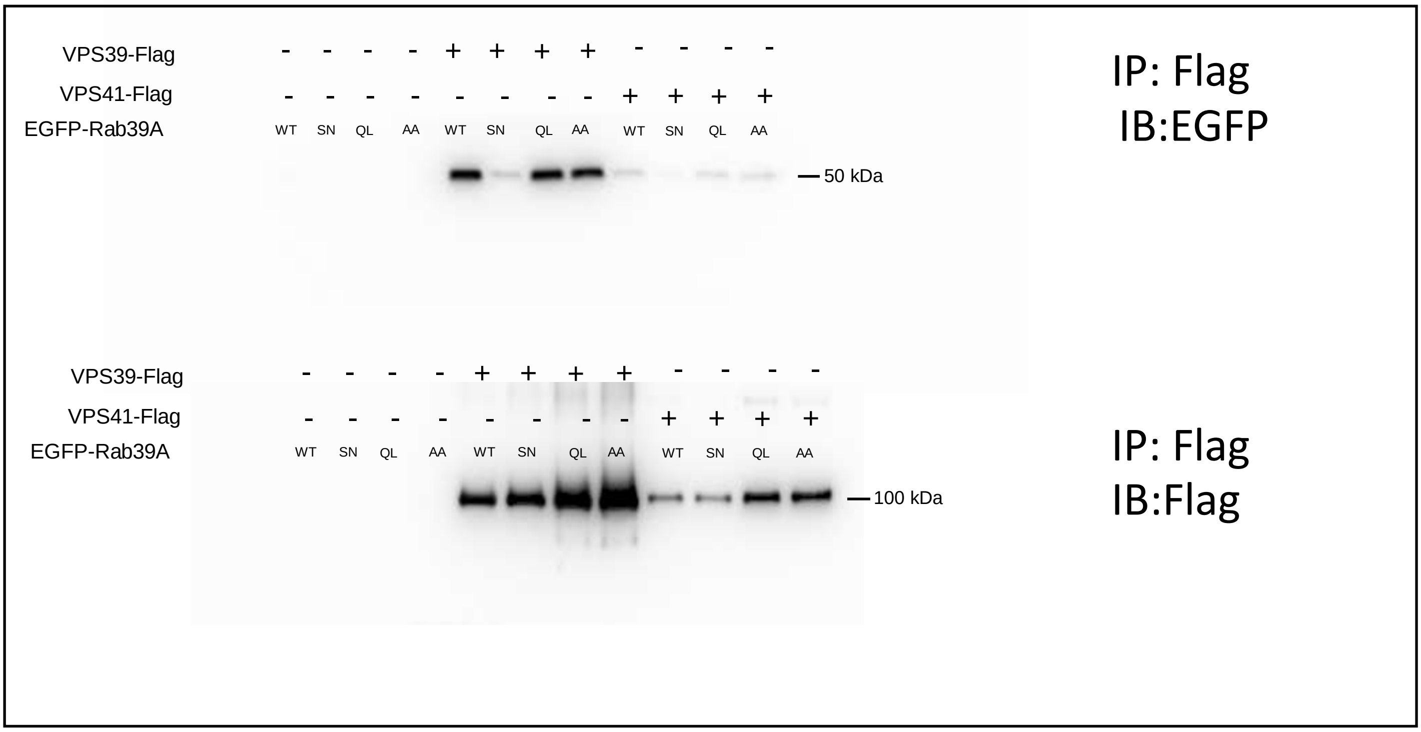

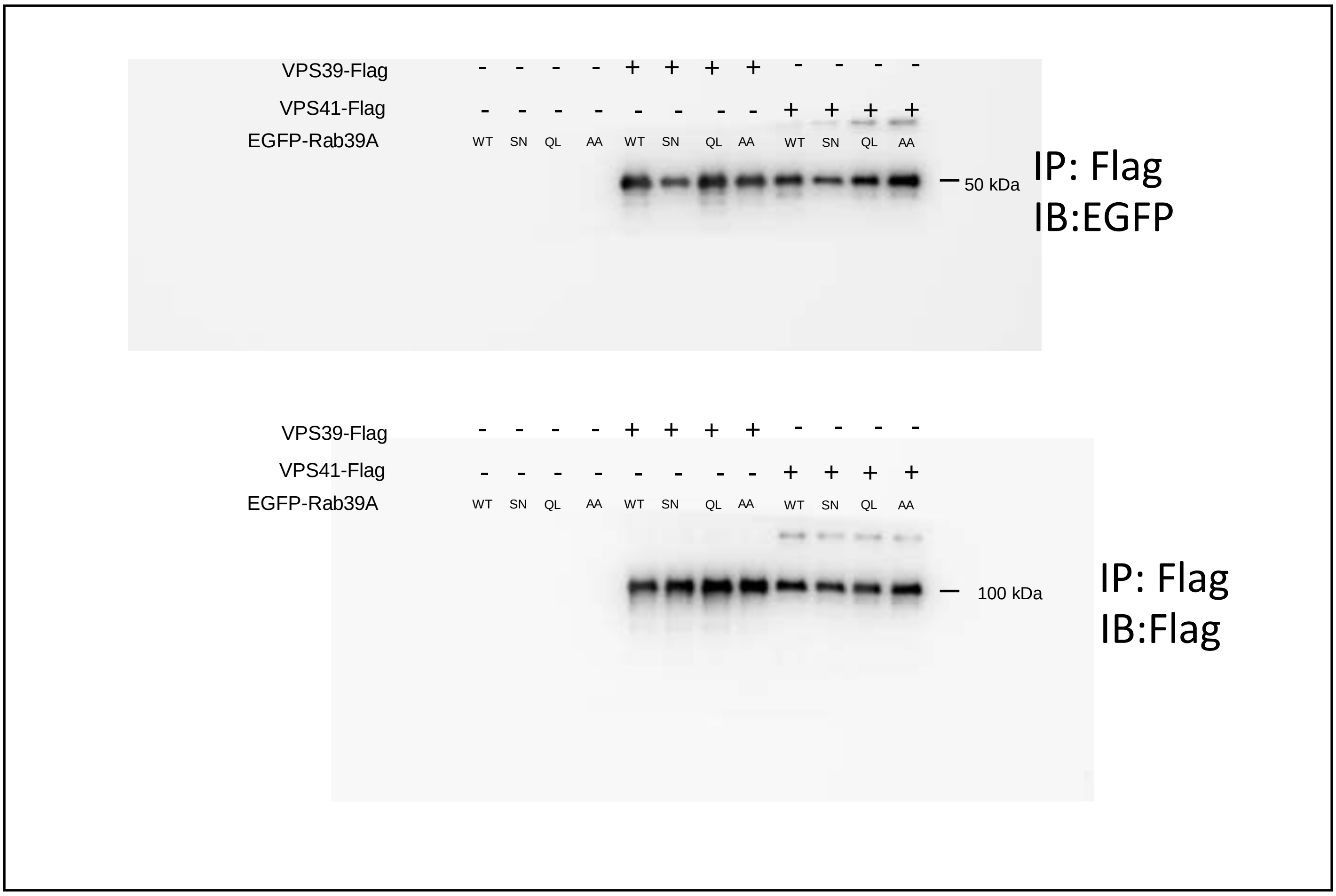

Figure 5d

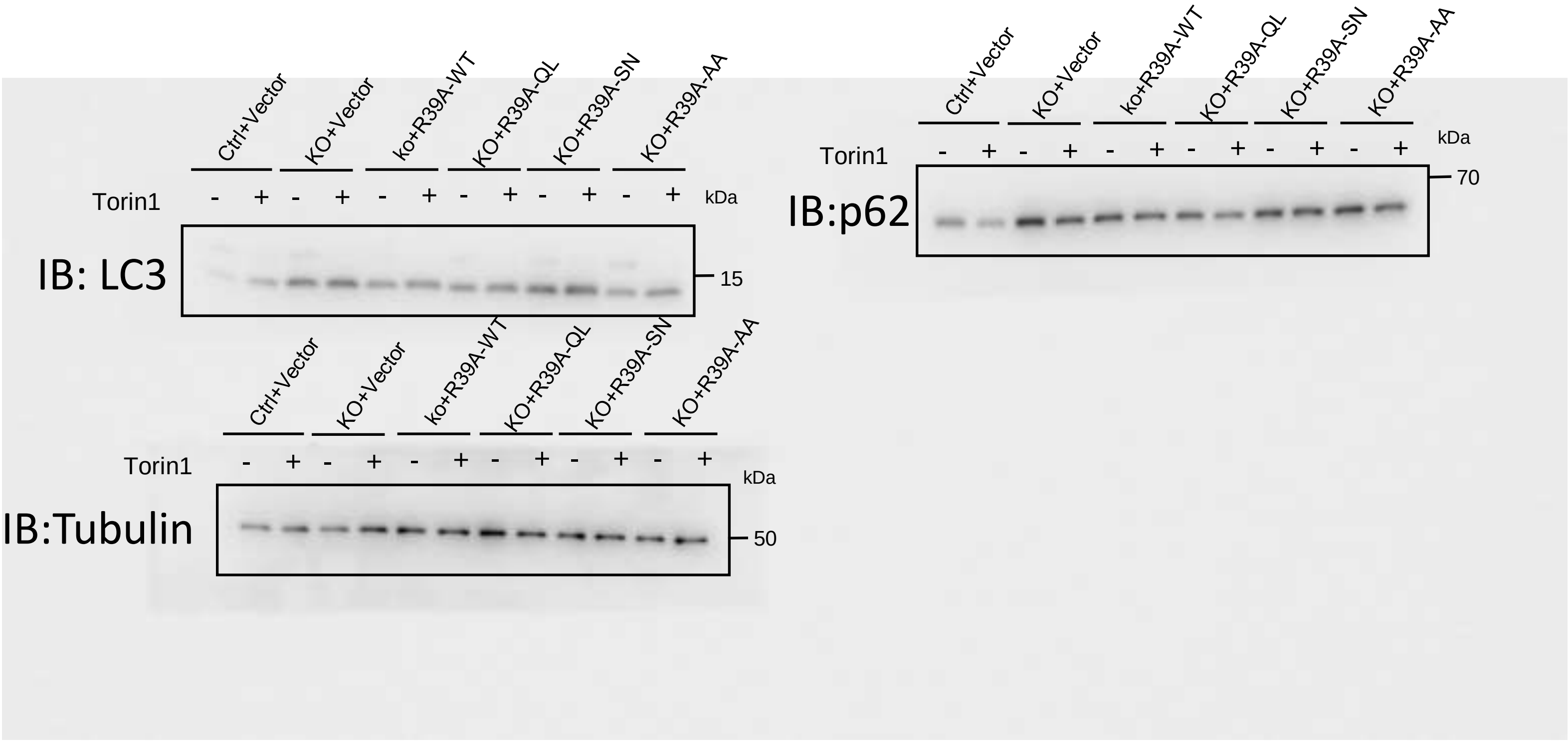
Ctrl+Vector
KO+Vector
ko+R39A-WT
KO+R39A-QL
KO+R39A-SN
KO+R39A-AA
Torin1
- + - + - + - + - + - +
kDa
IB: LC3
15
IB:p62
70
IB:Tubulin
50

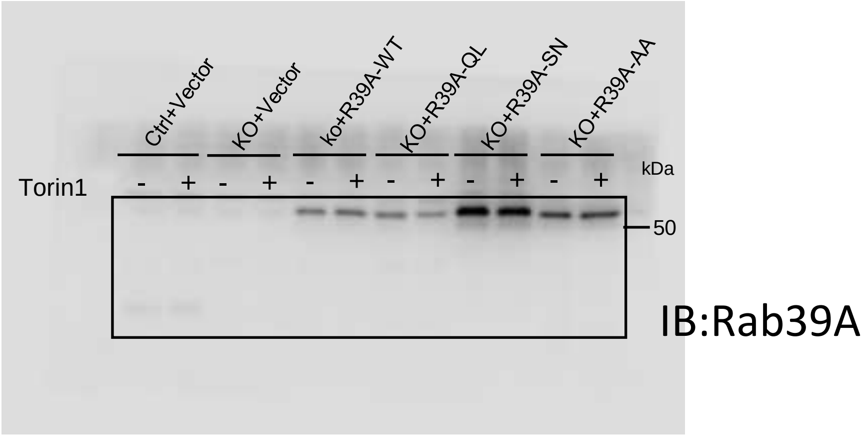
Ctrl+Vector
KO+Vector
ko+R39A-WT
KO+R39A-QL
KO+R39A-SN
KO+R39A-AA
Torin1
- + - + - + - + - + - +
kDa
50
IB:Rab39A

Figure 5d

Results of independent repeats:

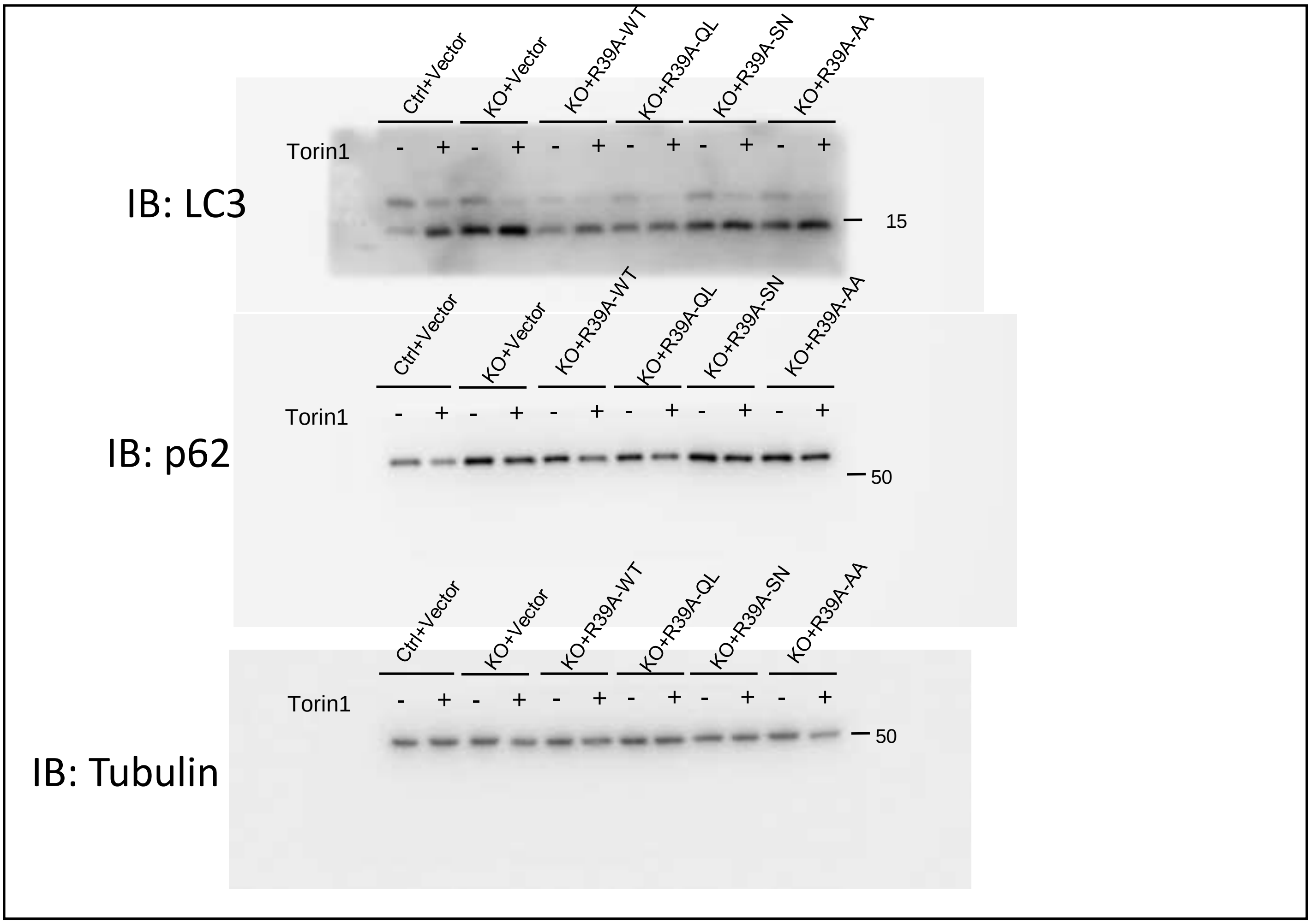

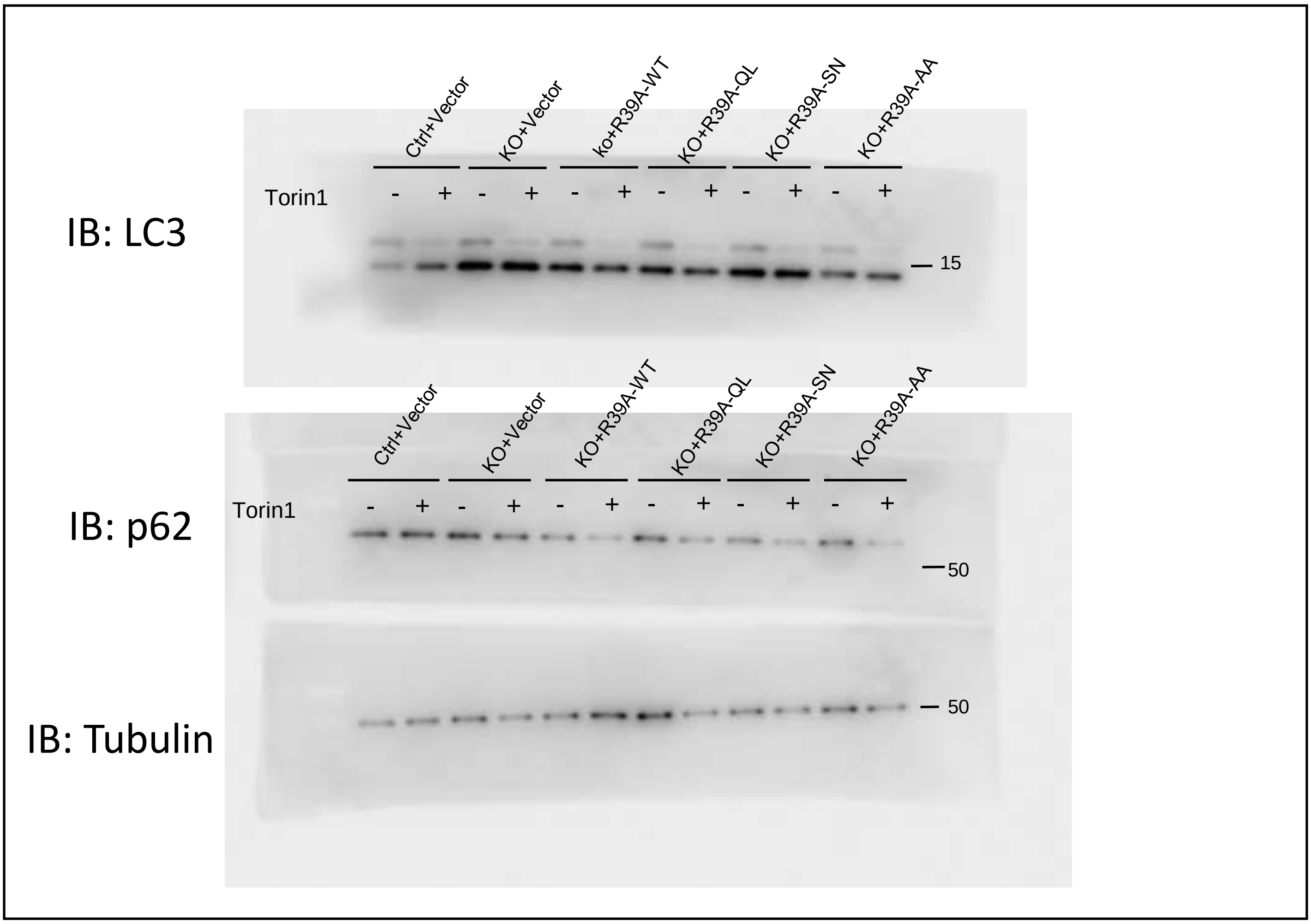

Figure 6

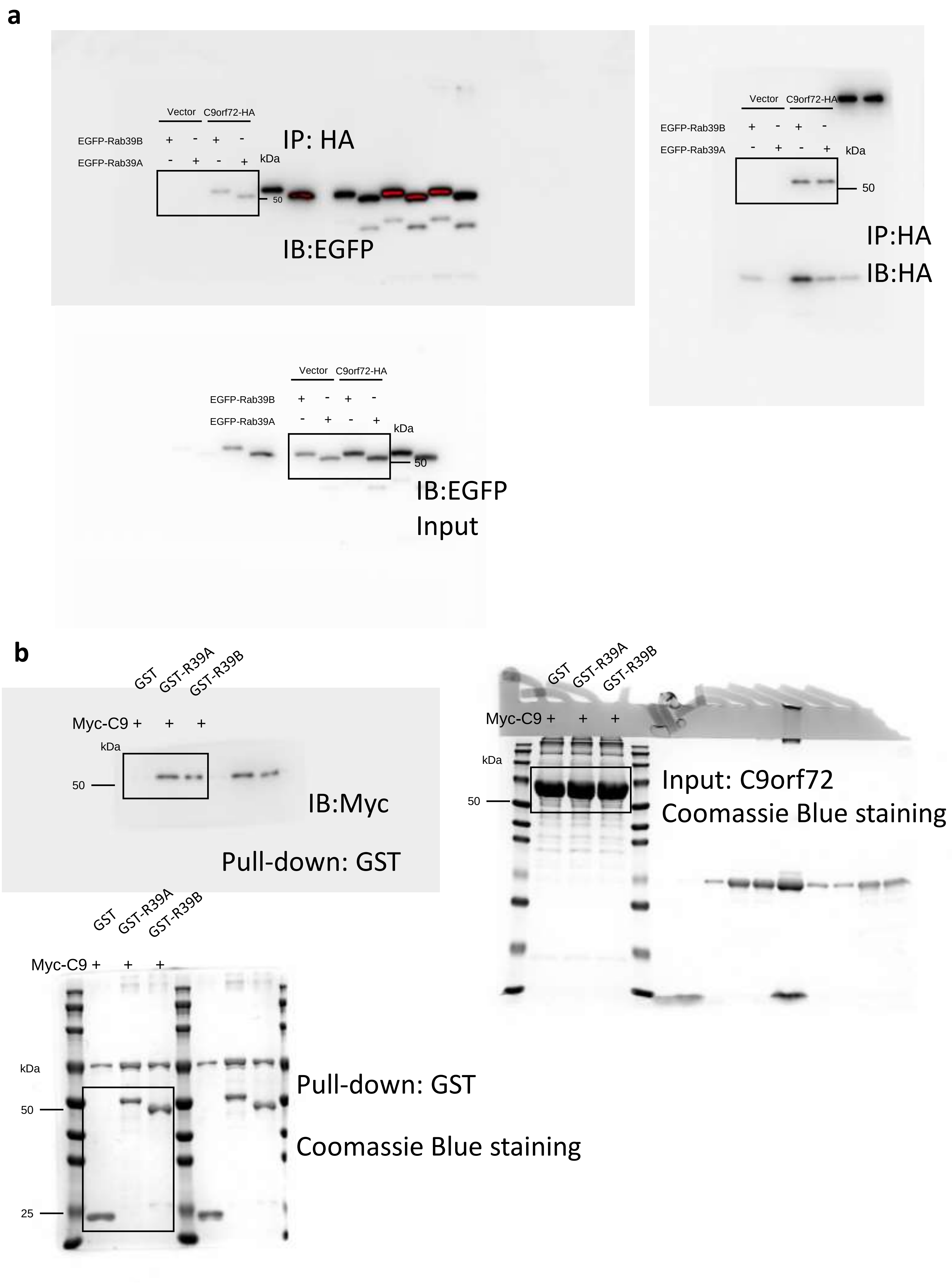
a
Vector
C9orf72-HA
EGFP-Rab39B + - + -
EGFP-Rab39A - + - +
kDa
50
IP: HA
IB:EGFP
IP:HA
IB:HA
IB:EGFP
Input
b
GST
GST-R39A
GST-R39B
Myc-C9 + + +
kDa
50
IB:Myc
Pull-down: GST
Input: C9orf72
Coomassie Blue staining
25
Pull-down: GST
Coomassie Blue staining

Figure 6c

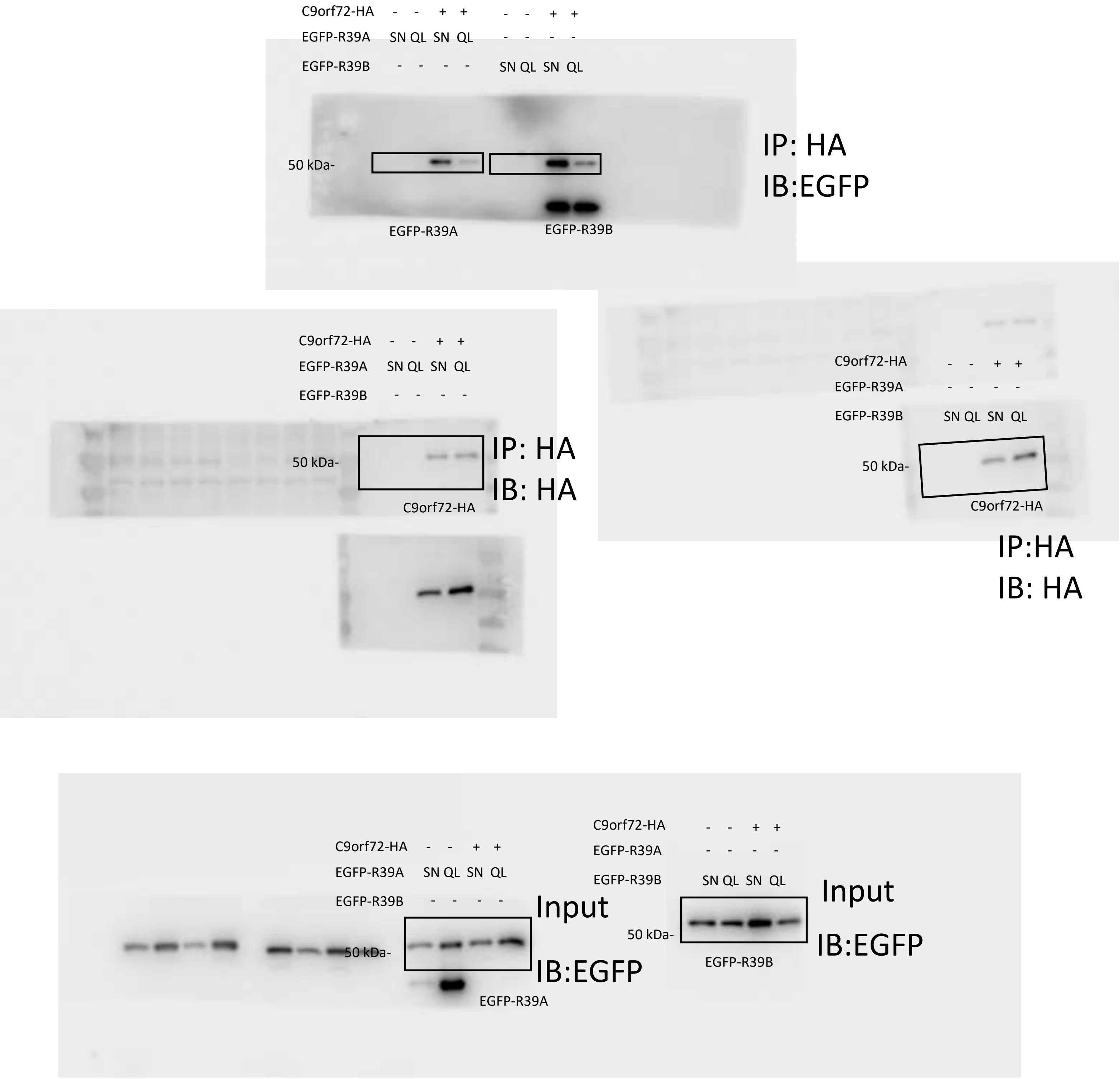
C9orf72-HA - - + + - - + +
EGFP-R39A SN QL SN QL - - - -
EGFP-R39B - - - - SN QL SN QL
50 kDa-
EGFP-R39A
EGFP-R39B
IP: HA
IB:EGFP
C9orf72-HA - - + +
EGFP-R39A SN QL SN QL
EGFP-R39B - - - -
50 kDa-
C9orf72-HA
IP: HA
IB: HA
C9orf72-HA - - + +
EGFP-R39A - - - -
EGFP-R39B SN QL SN QL
50 kDa-
C9orf72-HA
IP:HA
IB: HA
C9orf72-HA - - + +
EGFP-R39A SN QL SN QL
EGFP-R39B - - - -
50 kDa-
EGFP-R39A
Input
IB:EGFP
C9orf72-HA - - + +
EGFP-R39A - - - -
EGFP-R39B SN QL SN QL
50 kDa-
EGFP-R39B
Input
IB:EGFP

Figure 6c

Results of independent repeats:

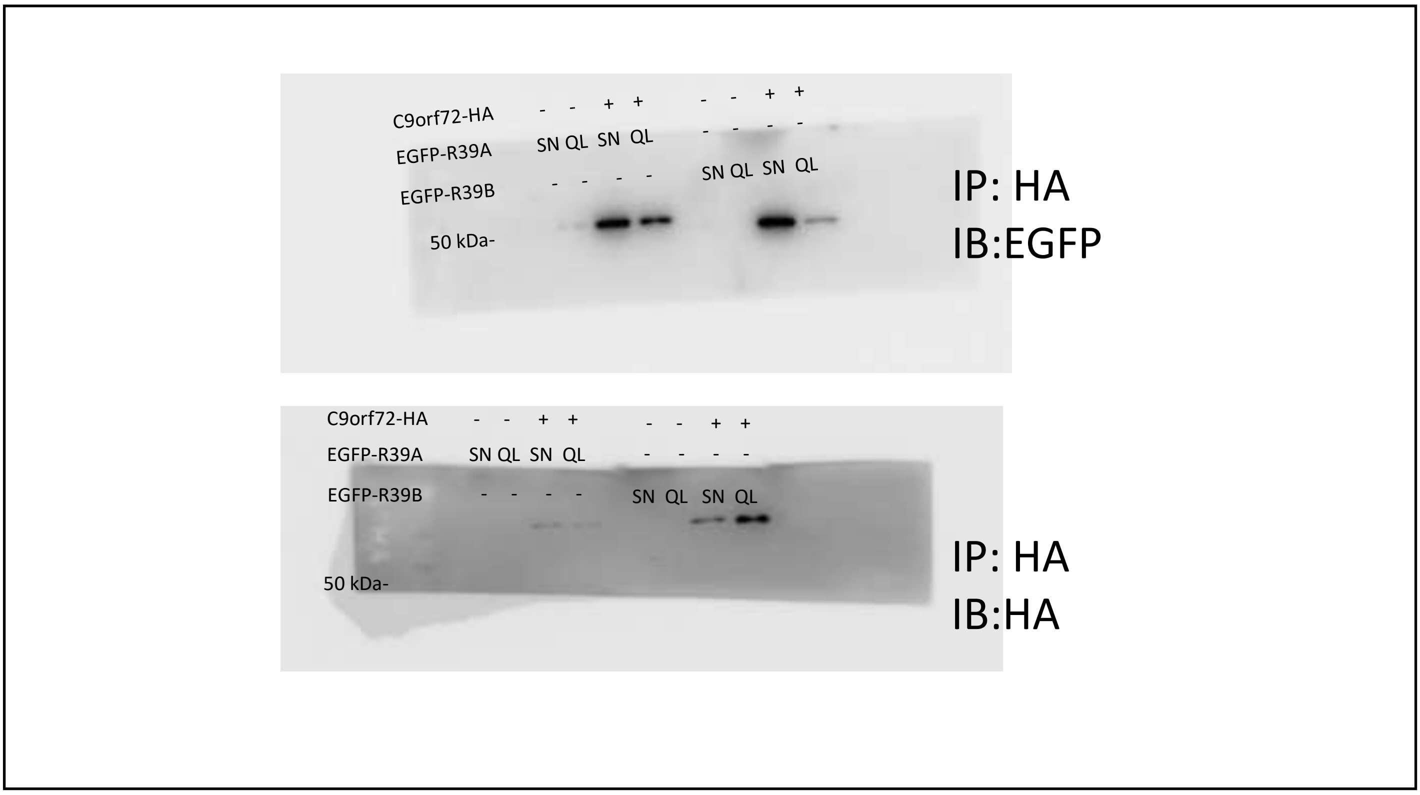

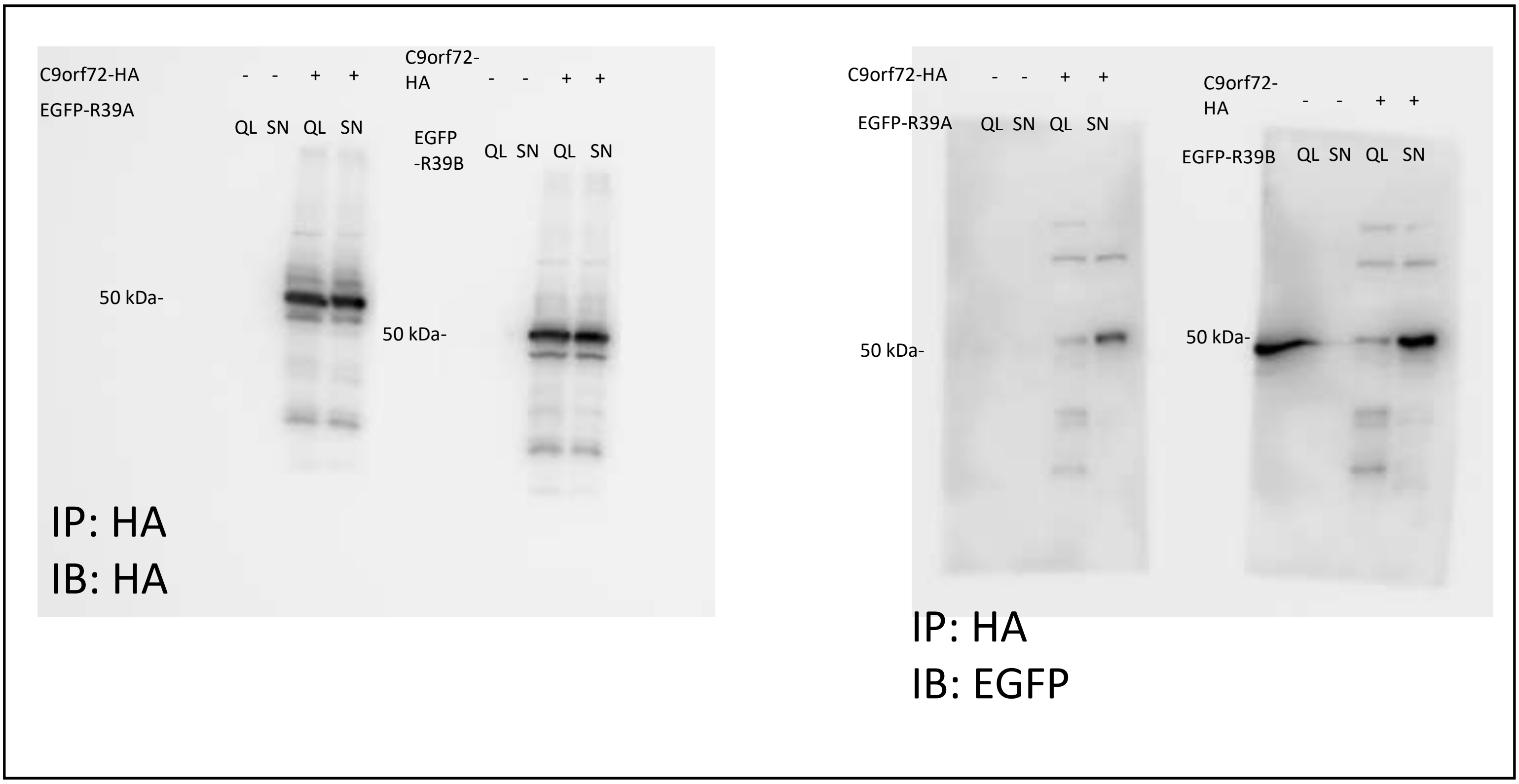

Figure 7a

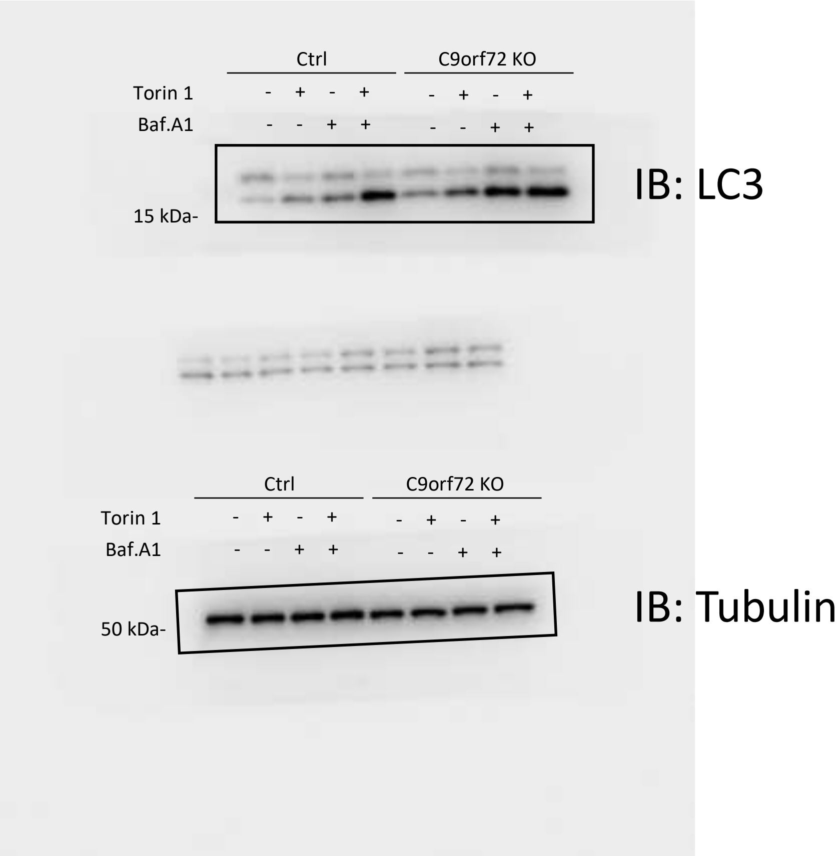
Ctrl
C9orf72 KO
Torin 1 - + - + - + - +
Baf.A1 - - + + - - + +
15 kDa-
IB: LC3
Ctrl
C9orf72 KO
Torin 1 - + - + - + - +
Baf.A1 - - + + - - + +
50 kDa-
IB: Tubulin

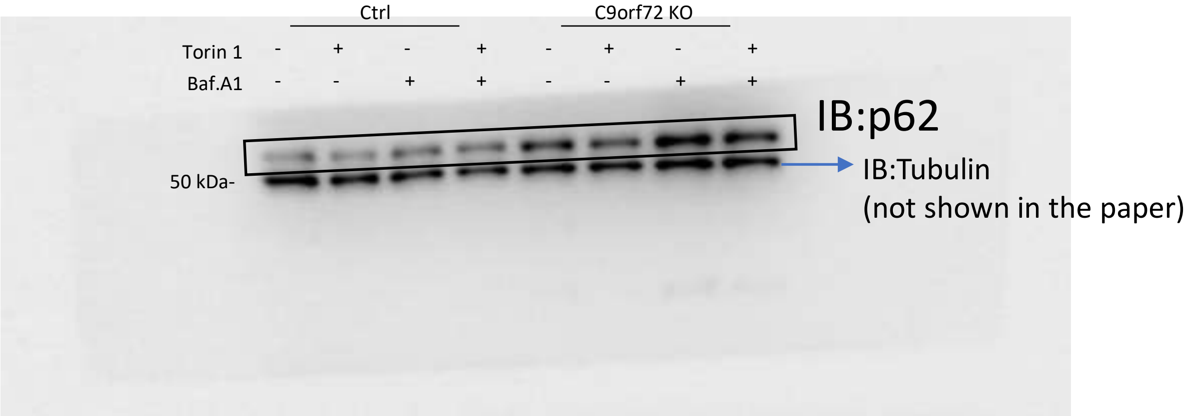
Ctrl
C9orf72 KO
Torin 1 - + - + - + - +
Baf.A1 - - + + - - + +
50 kDa-
IB:p62
IB:Tubulin
(not shown in the paper)

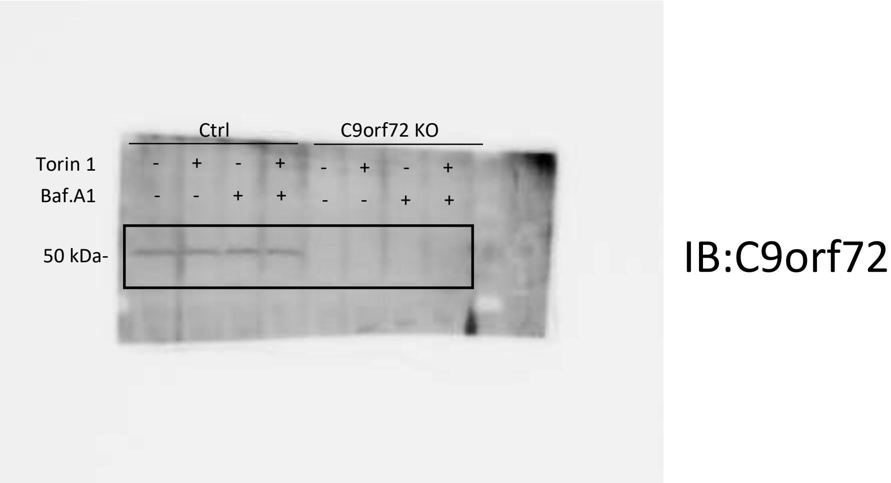
Ctrl
C9orf72 KO
Torin 1 - + - + - + - +
Baf.A1 - - + + - - + +
50 kDa-
IB:C9orf72

Figure 7a

Results of independent repeats:

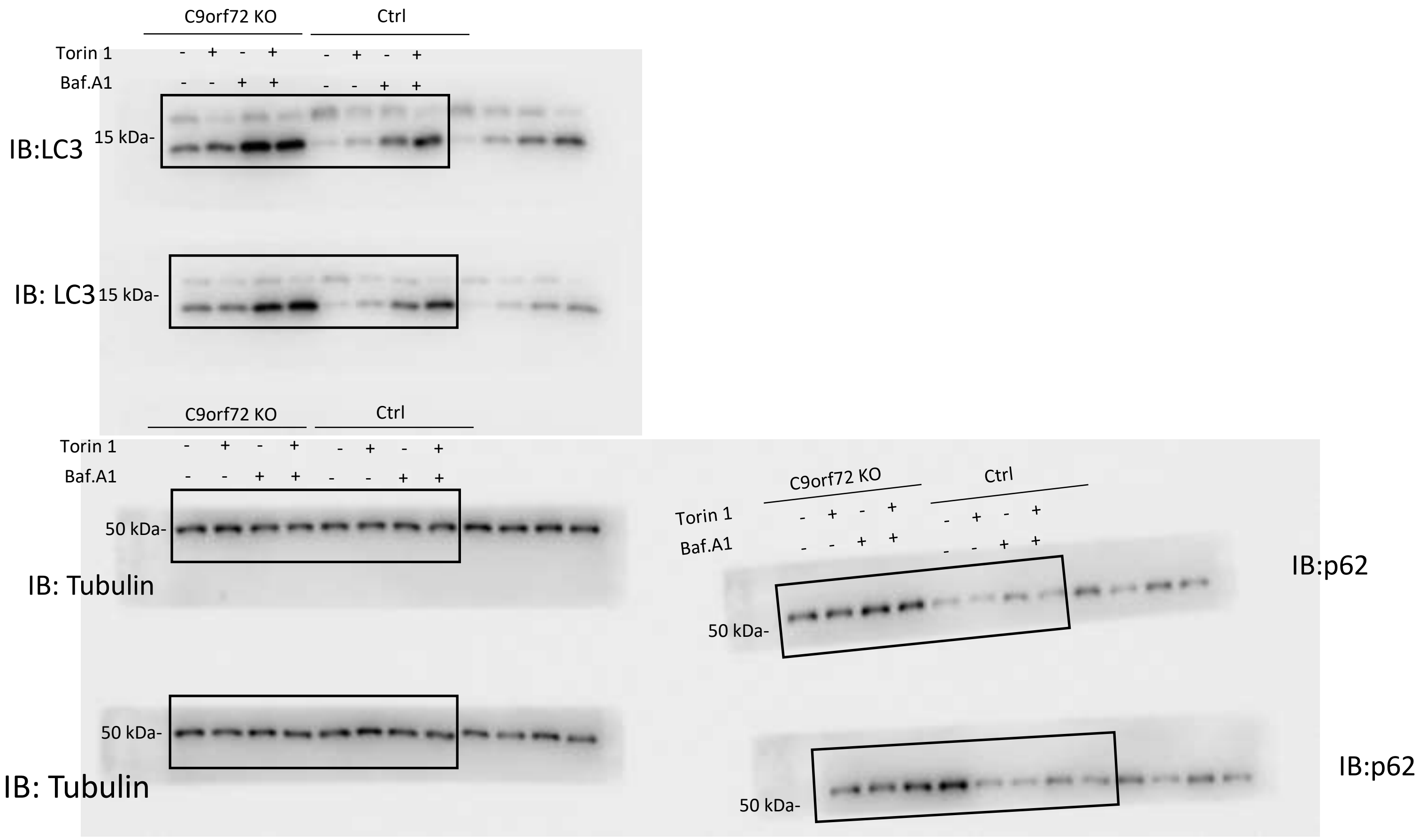

Figure7

h

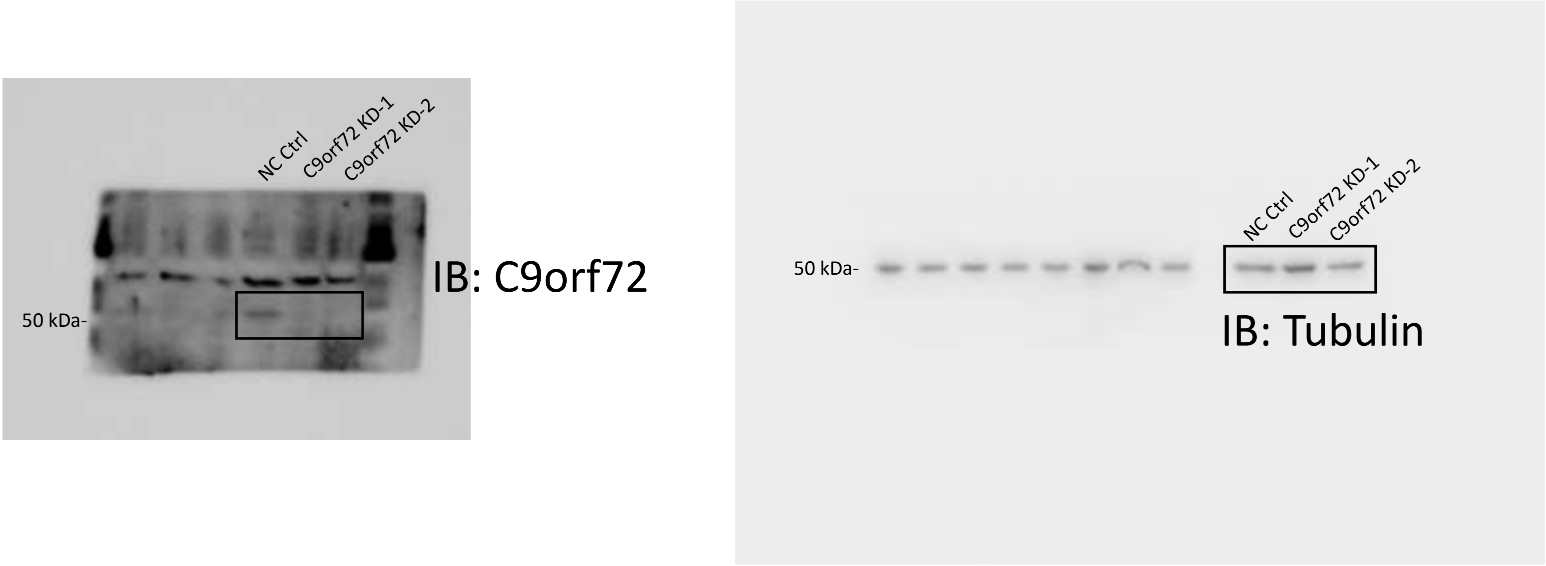

NC Ctrl
C9orf72 KD-1
C9orf72 KD-2
50 kDa-
IB: C9orf72
NC Ctrl
C9orf72 KD-1
C9orf72 KD-2
50 kDa-
IB: Tubulin

i

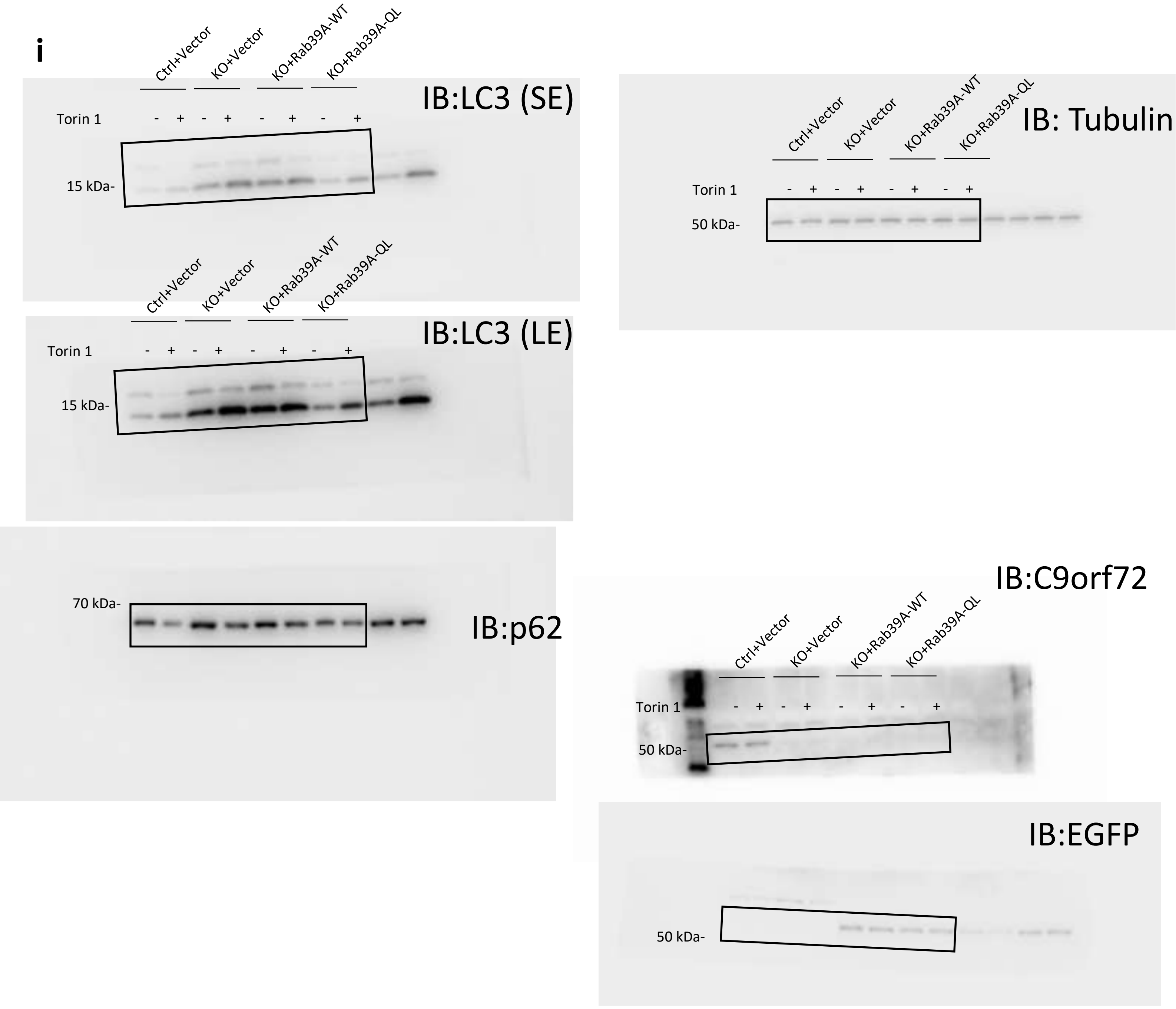

Ctrl+Vector
KO+Vector
KO+Rab39A-WT
KO+Rab39A-QL
IB:LC3 (SE)
Torin 1
- + - + - + - +
15 kDa-
Ctrl+Vector
KO+Vector
KO+Rab39A-WT
KO+Rab39A-QL
IB:LC3 (LE)
Torin 1
- + - + - + - +
15 kDa-
70 kDa-
IB:p62
Ctrl+Vector
KO+Vector
KO+Rab39A-WT
KO+Rab39A-QL
IB: Tubulin
Torin 1
- + - + - + - +
50 kDa-
IB:C9orf72
Ctrl+Vector
KO+Vector
KO+Rab39A-WT
KO+Rab39A-QL
Torin 1
- + - + - + - +
50 kDa-
IB:EGFP
50 kDa-

Figure 7i

Results of independent repeats:

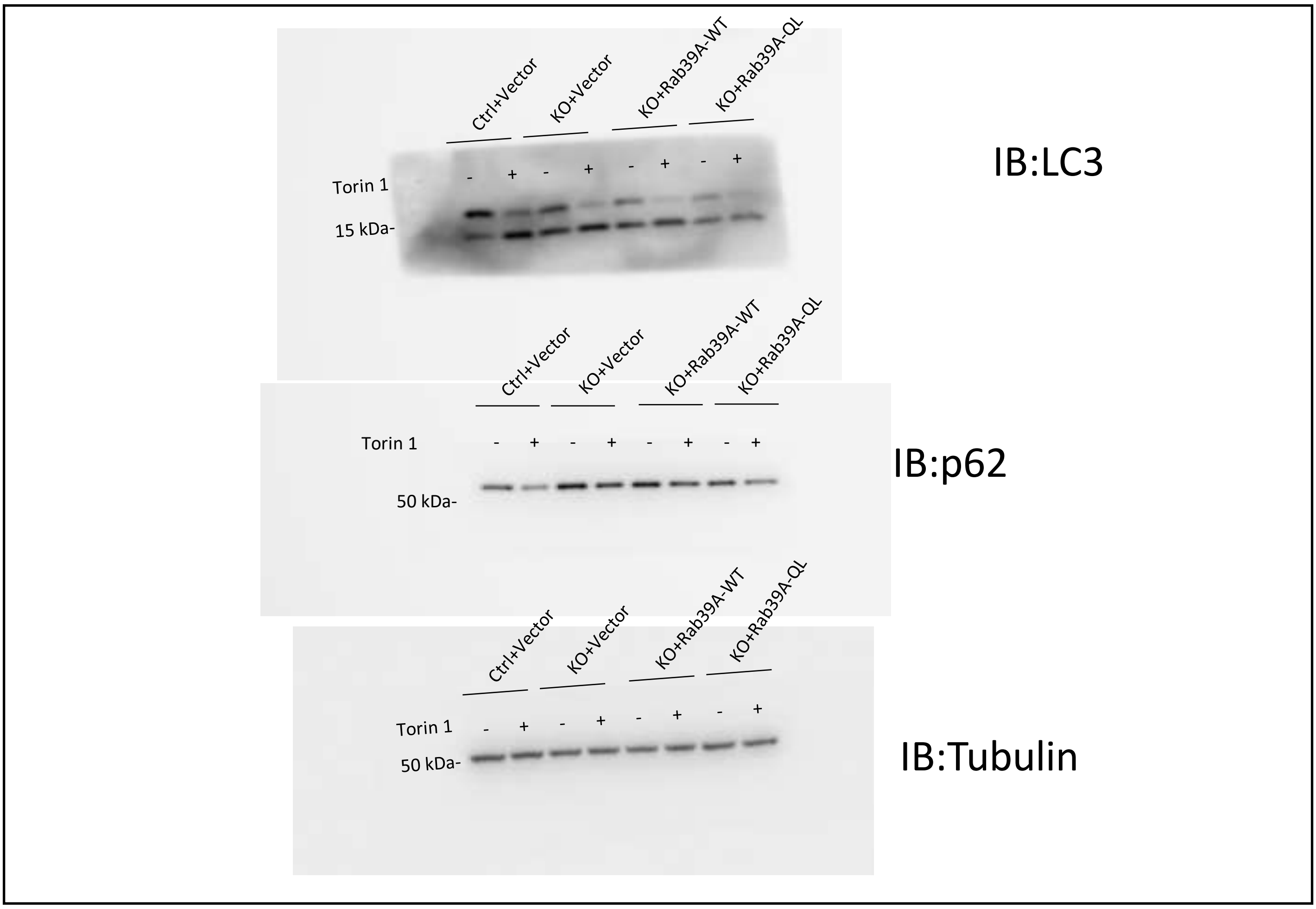

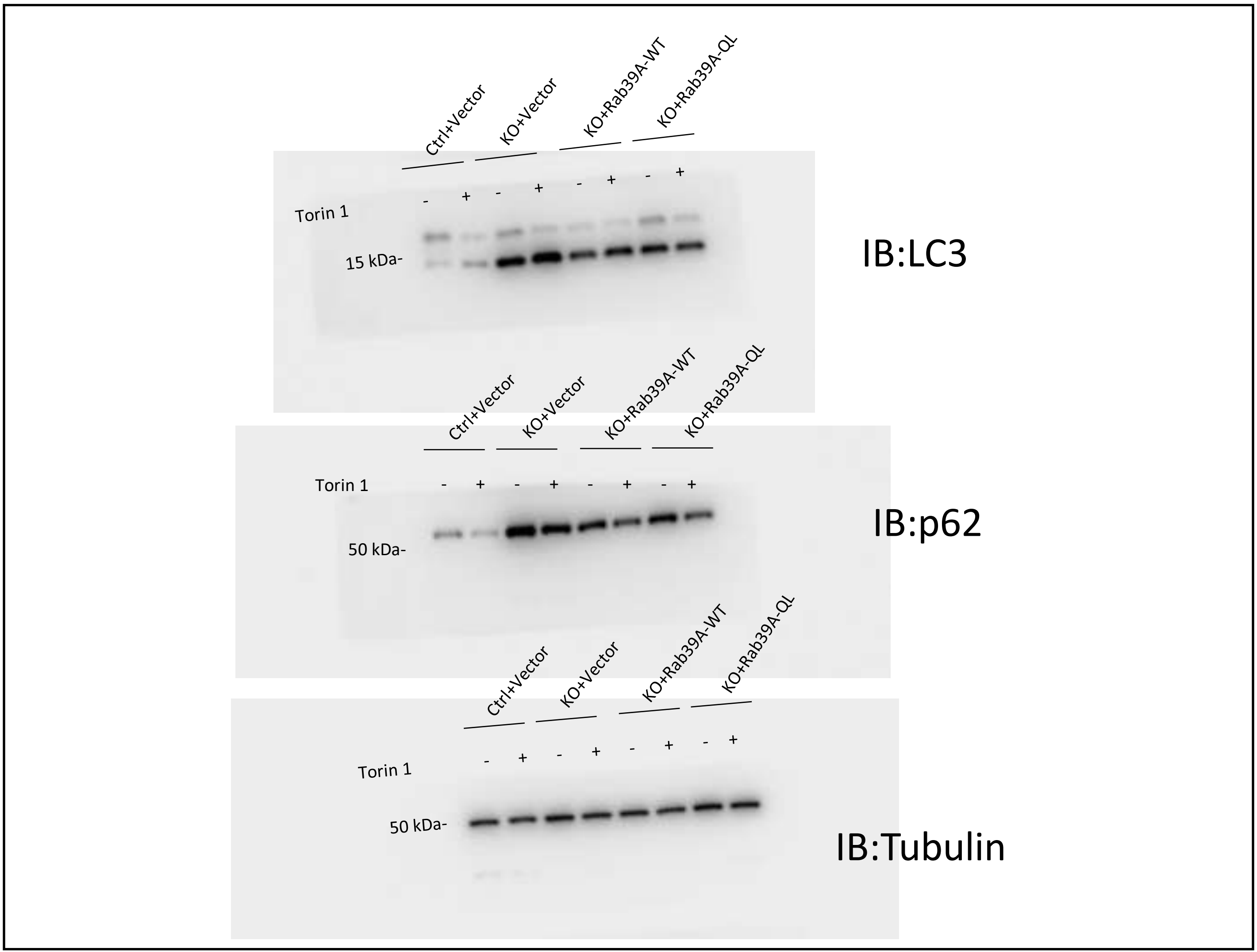

Supplement: Supplementary file 5 — Source Data [file 41467_2023_42003_MOESM5_ESM.zip › NCOMMS-22-37732B_MAIN_GELS.pdf]
